# Orbital Metastases: A Systematic Review of Clinical Characteristics, Management Strategies, and Treatment Outcomes

**DOI:** 10.3390/cancers14010094

**Published:** 2021-12-24

**Authors:** Paolo Palmisciano, Gianluca Ferini, Christian Ogasawara, Waseem Wahood, Othman Bin Alamer, Aditya D. Gupta, Gianluca Scalia, Alexandra M. G. Larsen, Kenny Yu, Giuseppe E. Umana, Aaron A. Cohen-Gadol, Tarek Y. El Ahmadieh, Ali S. Haider

**Affiliations:** 1 Department of Neurosurgery, Trauma Center, Gamma Knife Center, Cannizzaro Hospital, 95126 Catania, Italy; umana.nch@gmail.com; 2 Department of Radiation Oncology, REM Radioterapia srl, 95029 Viagrande, Italy; gianluca.ferini@grupposamed.com; 3 John A. Burns School of Medicine, University of Hawai’i, Honolulu, HI 96813, USA; cogasawa@hawaii.edu; 4 Kiran C. Patel College of Allopathic Medicine, Nova Southeastern University, Davie, FL 33328, USA; ww412@mynsu.nova.edu; 5 Department of Neurosurgery, King Abdullah International Medical Research Center, Riyadh 11451, Saudi Arabia; oabinalamer@gmail.com; 6 College of Medicine, Texas A&M University, Houston, TX 77030, USA; aditya@tamu.edu (A.D.G.); AAlam@mdanderson.org (A.S.H.); 7 Department of Neurosurgery, Highly Specialized Hospital and of National Importance “Garibaldi”, 95126 Catania, Italy; gianluca.scalia@outlook.it; 8 Department of Neurosurgery, Brain Metastasis Center, Memorial Sloan Kettering Cancer Center, New York, NY 10065, USA; giantina@mskcc.org (A.M.G.L.); yuk2@mskcc.org (K.Y.); ElAhmadT@mskcc.org (T.Y.E.A.); 9 Department of Neurological Surgery, Indiana University School of Medicine, Indianapolis, IN 46077, USA; cohen@nsatlas.com; 10 Department of Neurosurgery, Anderson Cancer Center, The University of Texas M.D., Houston, TX 77030, USA

**Keywords:** orbital exenteration, orbital metastases, radiation oncology, skull base oncology, systematic review

## Abstract

**Simple Summary:**

Orbital metastases may significantly worsen the functional status of oncological patients, leading to debilitating visual impairments. Surgical resection, orbital exenteration, and complementary therapies may result in heterogeneous clinical outcomes. In this systematic review, we aimed to comprehensively analyze the current literature on orbital metastases, describing clinical and imaging features, available management, and treatment outcomes. We found that most orbital metastases occur at later stages after primary tumors, frequently showing diffuse location within the orbit and rarely invading intracranial structures. Biopsy-only techniques were more frequently preferred in view of the less invasive approaches, but surgical resection and orbital radiotherapy were related to improved clinical outcomes. Although patients with primary breast cancer and patients undergoing resection showed superior prognoses, overall survival rates were generally poor, suggesting the need to better understand orbital metastases’ microenvironments for devising optimal systemic treatment strategies.

**Abstract:**

Background: Orbital metastases often lead to severe functional impairment. The role of resection, orbital exenteration, and complementary treatments is still debated. We systematically reviewed the literature on orbital metastases. Methods: PubMed, Scopus, Web-of-Science, and Cochrane were searched upon PRISMA guidelines to identify studies on orbital metastases. Clinical characteristics, management strategies, and survival were analyzed. Results: We included 262 studies comprising 873 patients. Median age was 59 years. The most frequent primary tumors were breast (36.3%), melanoma (10.1%), and prostate (8.5%) cancers, with median time interval of 12 months (range, 0–420). The most common symptoms were proptosis (52.3%) and relative-afferent-pupillary-defect (38.7%). Most metastases showed a diffuse location within the orbit (19%), with preferential infiltration of orbital soft tissues (40.2%). In 47 cases (5.4%), tumors extended intracranially. Incisional biopsy (63.7%) was preferred over fine-needle aspiration (10.2%), with partial resection (16.6%) preferred over complete (9.5%). Orbital exenteration was pursued in 26 patients (3%). A total of 305 patients (39.4%) received chemotherapy, and 506 (58%) received orbital radiotherapy. Post-treatment symptom improvement was significantly superior after resection (*p* = 0.005) and orbital radiotherapy (*p* = 0.032). Mean follow-up was 14.3 months, and median overall survival was 6 months. Fifteen cases (1.7%) demonstrated recurrence with median local control of six months. Overall survival was statistically increased in patients with breast cancer (*p* < 0.001) and in patients undergoing resection (*p* = 0.024) but was not correlated with orbital location (*p* = 0.174), intracranial extension (*p* = 0.073), biopsy approach (*p* = 0.344), extent-of-resection (*p* = 0.429), or orbital exenteration (*p* = 0.153). Conclusions: Orbital metastases severely impair patient quality of life. Surgical resection safely provides symptom and survival benefit compared to biopsy, while orbital radiotherapy significantly improves symptoms compared to not receiving radiotherapy.

## 1. Introduction

Orbital metastases represent 1–13% of all orbital neoplasms and affect approximately 2–5% of patients with systemic malignancies [1,2,3]. Breast, melanoma, and prostate cancers comprise the prevalent primary tumors, and their incidence is increasing due to improved surveillance, systemic disease control and management of oncological patients [3,4,5]. Orbital metastases can often be detected in those with no previous history of cancer due to their common presenting of symptoms of visual disturbance, thus preceding the diagnosis of primary tumors [4,6]. Common presenting symptoms include diplopia, ocular pain, and vision loss, coupled with globe displacement and palpable orbital masses [7,8]. Metastases frequently appear on imaging as irregular and contrast-enhancing lesions in the anterior orbit involving bones and extraocular muscles [8,9,10].

Treatment strategies depend on the clinical presentation and primary tumor pathology; however, a gold standard for treatment has yet to be defined [3,11]. In poor surgical candidates, orbital irradiation can be utilized to facilitate reduction in tumor volume and symptom relief [12]. Surgical debulking is effective in decreasing mass-effect and improving symptoms but can also lead to serious complications such as permanent visual deficits [13]. Chemotherapy, hormonal therapy, and/or targeted therapy have the benefit of simultaneous control of primary and metastatic lesions [7,9].

There is a limited number of individual studies on orbital metastases, and feasible therapeutic options are still debated [14,15]. In this systematic review, we evaluate the clinical features and management strategies of patients with orbital metastases, focusing on factors related to symptom improvement and survival.

## 2. Materials and Methods

### 2.1. Literature Search

A systematic review was conducted following the Preferred Reporting Items for Systematic Reviews and Meta-Analyses (PRISMA) guidelines [16] and registered to PROSPERO (ID: 293984). PubMed, Scopus, Web of Science, and Cochrane were searched from database inception to 10 May 2021 using the combination of the Boolean operators “OR” and “AND”, and the search terms “orbital” and “metastases”. Studies were exported to Mendeley; duplicates were removed.

### 2.2. Study Selection

Inclusion and exclusion criteria were set a priori. Studies were included if they (1) involved ≥ 1 patients aged ≥ 18 years with histologically confirmed distant metastases originating from solid tumors and affecting periocular intra-orbital structures; (2) reported clinical, management and survival data; (3) were written in English. Studies were excluded if they (1) were reviews, technical notes, or autopsy reports; (2) contained only patients with intraocular/eyelid metastases and/or craniofacial/ocular tumors directly extending into the orbit; (3) did not clearly differentiate data between patients with orbital metastases and patients with intraocular metastases or primary orbital lesions; (4) contained insufficient clinical and management data.

Two authors (C.S. and P.P.) independently screened titles and abstracts of all collected citations and subsequently assessed full texts of articles that met the inclusion criteria. A third author (A.S.H.) settled any disagreements. Eligible articles were included, and references were searched to retrieve additional relevant studies.

### 2.3. Data Extraction

Data were extracted from one author (P.P.) and then independently verified by two additional authors (A.S.H. and O.B.A.). Missing data were either not reported or not differentiable from other data. Data included author, year, study design, age, gender, primary tumor, time interval between primary tumor and orbital metastasis, laterality, orbital localization, tissue infiltration, intracranial extension, symptoms, imaging features, extent-of-surgery, surgery techniques, complementary treatment strategies, radiation protocols (i.e., type, fractionation, total dose), clinical/radiological treatment responses, orbital metastases recurrence, local control (LC), overall survival (OS), and survival status. Extent-of-surgical resection was defined as “complete resection” for 100% tumor resection and “partial resection” for <100% tumor resection. Clinical and radiological treatment responses were assessed at available time points or at last follow-up. Radiological responses were evaluated following the proposed RANO criteria, describing post-treatment lesions’ volumetric changes in patients undergoing tumor biopsy and chemotherapy and/or radiotherapy: complete response (CR)—complete resolution; partial response (PR)—decreased volume; stable disease (SD)—no volume change; progression (PD)—increased volume [17].

### 2.4. Data Synthesis and Quality Assessment

Primary outcomes of interest were clinical characteristics, management strategies, and survival analysis of orbital metastases. Levels of evidence were assessed upon the 2011 Oxford Centre For Evidence-Based Medicine guidelines [18]. Meta-analysis was precluded because all included articles had levels IV-V of evidence, and hazard ratios could not be deducted. Risk-of-bias was independently appraised by two authors (P.P. and O.B.A.) using the Joanna Briggs Institute checklists for case reports and case series [19,20].

### 2.5. Statistical Analysis

The software SPSS V.25 (IBM Corp, Armonk, New York, NY, USA) was used for all statistical analyses, and a bilateral *p*-value < 0.05 was considered significant for all tests. Continuous variables are reported as medians or means with ranges and categorical variables reported as frequencies and percentages. Clinical and radiologic treatment responses for patients undergoing biopsy and surgical resection were compared using χ2 and Fisher exact tests. Multivariate logistic regression analyses were conducted to ascertain variables associated with clinical and radiological treatment responses, including all the variables deemed of potential relevance—corresponding to a cut-off of *p* < 0.05 at the univariate analysis—and retained in the multivariate model with a statistically significant power (*p* < 0.05). In a similar fashion, a multivariate Cox proportional-hazards model was performed to identify variables correlated with OS. Kaplan–Meier methods and log-rank tests were used to compare survival outcomes based on patient-level clinical and treatment characteristics.

## 3. Results

### 3.1. Study Selection

**Figure 1:**
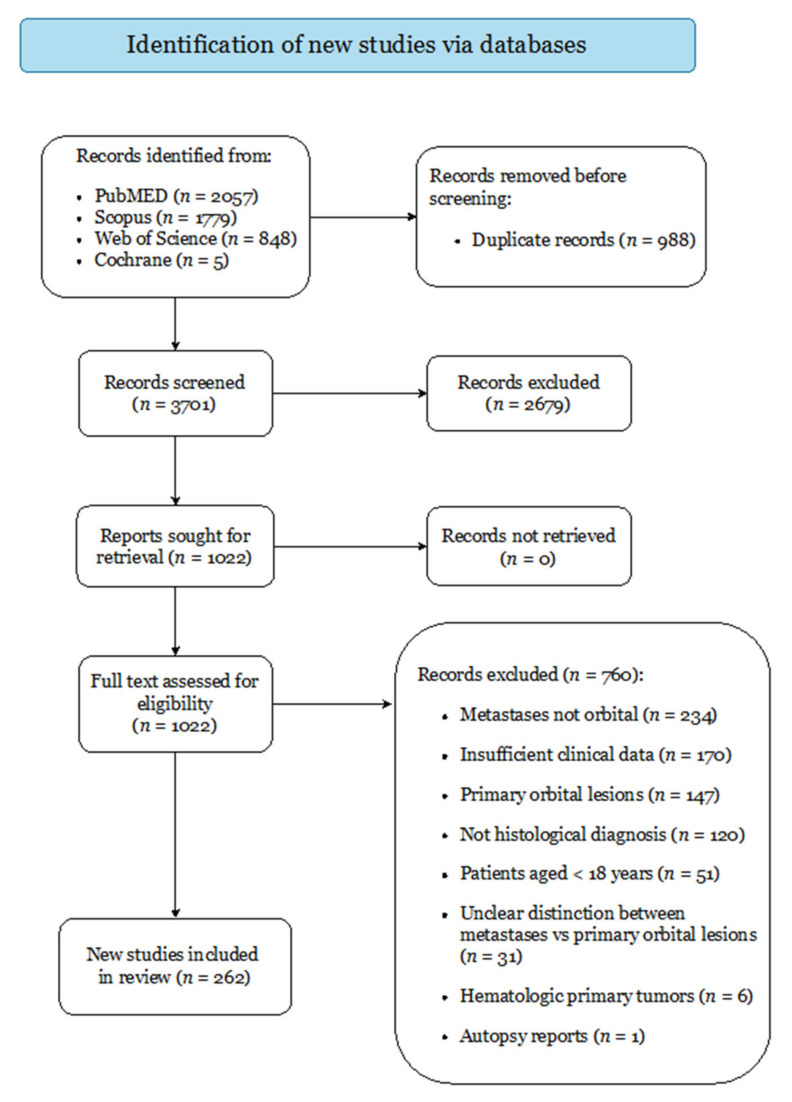
PRISMA 2020 Flow-Diagram.

Figure 1 displays the study selection process. The initial search yielded 4689 articles, of which 262 were finally included in accordance with the pre-determined criteria. Forty were case series (including 651 patients) and 222 were case reports, categorized as evidence level IV and V, respectively (overview and references of all included studies are reported in Table S1). Critical appraisal returned low risk of bias for all included studies (Table S2).

### 3.2. Demographics and Clinical Characteristics

A total of 873 patients were included (Table 1). Patients were predominantly female (56.7%), with a median age of 59 years (range, 18–90). Breast cancers were the most frequent primary lesions (36.3%), followed by melanoma (10.1%) and prostate cancers (8.5%), while 37 patients (4.2%) were diagnosed with carcinomas of unknown primary (CUP). Median time interval between primary tumor diagnosis and orbital metastasis was 12 months (range, 0–420), with orbital metastasis preceding primary tumor diagnosis in 218 cases (30.1%). No prevalence in orbital laterality was noted (left orbit 46.2%, right 46.1%), and 57 patients had bilateral lesions (7.7%). Metastases mostly showed a diffuse location within the orbit (19%), frequently infiltrating orbital soft tissues (40.2%) and extraocular muscles (26.8%). In 47 cases (15.7%), lesions extended intracranially into the anterior/middle fossae. Except for five cases (0.6%), all orbital metastases were symptomatic, frequently presenting with proptosis (52.3%), relative afferent pupillary defect (RAPD; 38.7%), and diplopia of the ipsilateral eye (35.5%). On imaging, orbital metastases uncommonly exhibited osteolytic (14.7%) or osteoblastic (1.3%) appearances.

### 3.3. Management Strategies and Treatment Outcomes

Most patients (556, 63.7%) underwent incisional biopsy, while noninvasive fine-needle aspiration biopsy (FNAB) was pursued in 89 (10.2%) (Table 2). Partial tumor resection (145, 16.6%) was preferred over complete resection (83, 9.5%), while orbital exenteration was performed in 26 patients (26/83, 31.3%) undergoing complete resection. Among 751 patients with available data on complementary treatments, 305 (40.6%) received chemotherapy, and 506 (67.4%) received orbital radiotherapy with a median dose of 35.5 Gy. Additional treatments were reported in 151 cases: hormonal therapy (128, 84.8%), immunotherapy (12, 7.9%), steroids (7, 4.6%), and radioiodine (4, 2.6%). Although included studies comprised a 56-year time period characterized by major advances in management of oncological patients, the lack of granular data on per-patient time-of-treatment precluded a detail analysis on clinical outcomes from a chronological point-of-view.

Data on post-treatment clinical responses were described in 315 patients, of which 203 (64.4%) showed symptom improvement. Among patients receiving complementary chemo/radiotherapy, symptom improvement was significantly higher after tumor resection than biopsy only (*p* = 0.007). There was no statistical difference in symptom improvement with greater extent of resection (*p* = 0.268) or biopsy approach comparing incisional biopsy to FNAB (*p* = 0.768). On multivariate analysis, symptom improvement rates were significantly superior in patients undergoing tumor resection compared to patients undergoing biopsy (OR: 1.985, 95% CI: 1.096–3.100, *p* = 0.005) and in patients receiving radiotherapy (OR: 1.311, 95% CI: 1.066–2.127, *p* = 0.032) (Table 3).

Data on post-treatment radiological responses were available in 287 patients. CR occurred in 52 patients (18.1%), PR in 143 (49.9%), SD in 18 (6.3%), and PD in 74 (25.8%). Among patients receiving complementary chemo/radiotherapy, radiological reduction in tumor volume (CR and PR) was significantly superior after tumor resection rather than biopsy only (*p* = 0.007), but no differences were noted based on biopsy approach (*p* = 0.699) or extent-of-resection (*p* = 0.477). On multivariate analysis, significantly worse rates of tumor volume reduction were found in patients with primary melanoma compared to patients with primary breast cancer (OR: 0.244, 95% CI: 0.076–0.783, *p* = 0.018). Significantly decreased rates of tumor volume reduction were also seen in patients with bilateral lesions compared to patients with unilateral lesions (OR: 0.217, 95% CI: 0.065–0.728, *p* = 0.013) (Table 4).

### 3.4. Survival

**Figure 2:**
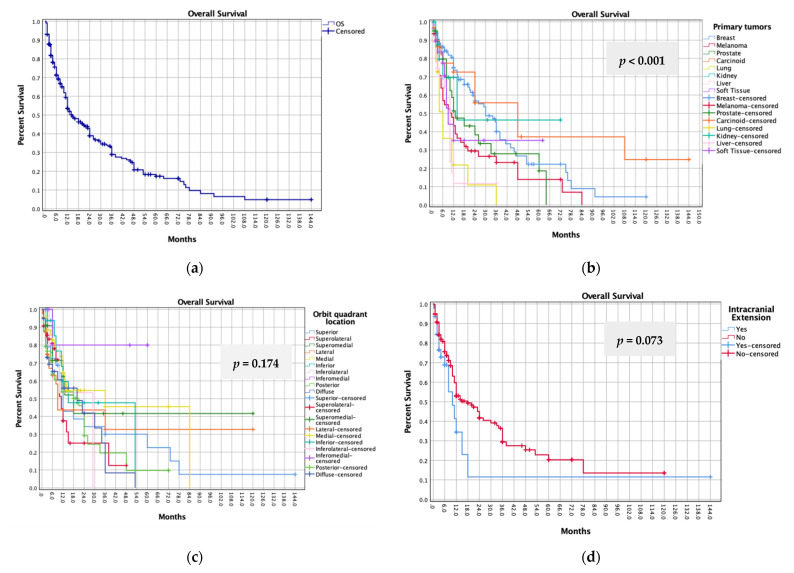

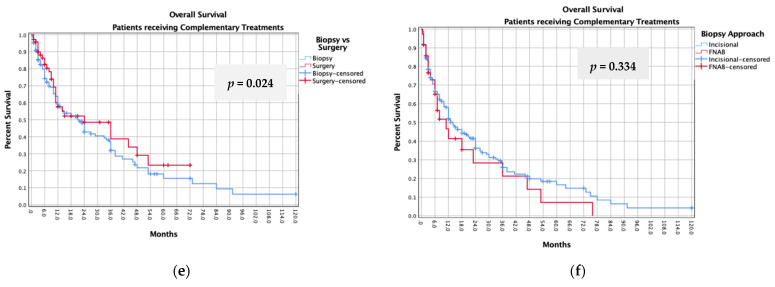

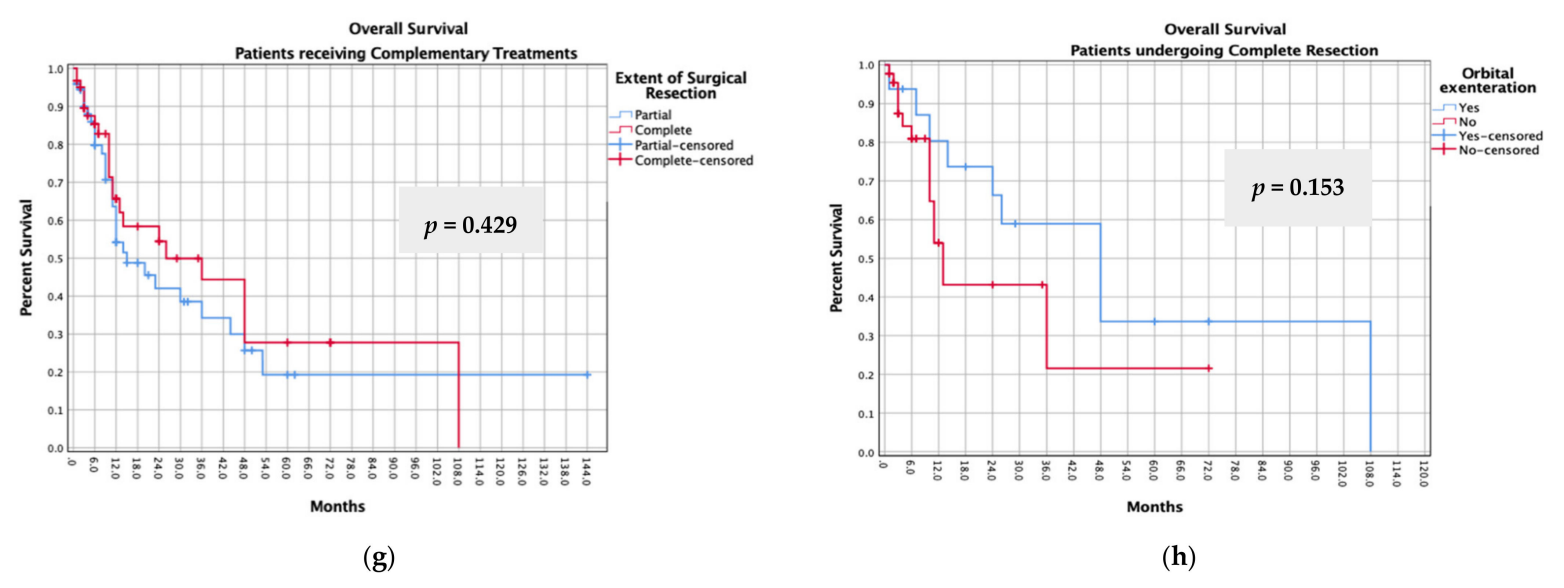
Kaplan–Meier curves of (**a**) overall survival of the pooled cohort; (**b**) overall survival based on the most common primary tumors; (**c**) overall survival based on orbital quadrant location; (**d**) overall survival based on intracranial extension; (**e**) OS (*n* = 377) based on biopsy vs. tumor resection in patients receiving complementary treatments (chemotherapy and/or radiotherapy); (**f**) overall survival based on biopsy approach (incisional vs. FNAB) in patients receiving complementary treatments; (**g**) overall survival based on complete (100%) vs. partial (<100%) tumor resection in patients receiving complementary treatments; (**h**) overall survival based on orbital exenteration in patients undergoing complete surgical resection.

Patients were followed for a mean of 14.3 months (range, 0.2–144). Recurrence of orbital metastases was reported in only 15 cases (1.7%), with a median LC of 6 months (range, 0.2–122). Among 641 patients with available survival data, there were 416 deaths (64.9%), with a median OS of 6 months (range, 0.2–144), a 1-year survival rate of 40%, and a 2-year survival rate of 29%. The log-rank tests showed a significant difference in OS when comparing patients with the 8 most common primary tumors (each with available patient-level data in ≥15 patients) (*p* < 0.001), with 1-year survival rates highest in breast-cancer (57%) and carcinoid (53%), and lowest in lung cancer (14%), liver cancer (27%), and melanoma (29%) (Figure 2). Among patients receiving complementary chemo/radiotherapy, we found statistically higher OS in patients undergoing tumor resection over patients undergoing biopsy (*p* = 0.024). No significant differences in OS were noted based on orbital location (*p* = 0.174), intracranial extension (*p* = 0.073), biopsy approach (*p* = 0.344), extent-of-resection (*p* = 0.429), and orbital exenteration (*p* = 0.153). Cox regression further confirmed the log-rank tests’ findings (Table 5). On multivariate analysis, melanoma (HR: 2.047, 95% CI: 1.353–3.096, *p* = 0.001), lung cancer (HR: 3.859, 95% CI: 2.130–6.993, *p* < 0.001), and liver cancer (HR: 3.249, 95% CI: 1.888–5.590, *p* < 0.001) were associated with poorer survival compared to breast cancer, while tumor resection was associated with improved survival compared to biopsy (HR:0.642, 95% CI:0.475–0.868, *p* = 0.004). We found no statistical difference in mortality with any other clinical or treatment variable.

## 4. Discussion

In this review, we found that surgical tumor resection significantly increased symptom relief and survival compared to biopsy-only. While orbital radiotherapy may effectively prevent tumor progression in the long term, the role of advanced systemic therapies requires further evaluation.

Although some differences in primary tumors have been reported between studies, probably mirroring the underlying geographic variations of cancer rates, we found that breast cancer (36.3%), melanoma (10.1%) and prostate cancer (8.5%) were the most frequent among the 29 different primary neoplasms included in this review [3,4,5]. Similar prevalence rates have been reported for patients with uveal metastases, likely suggesting common routes of tumoral hematogenous spread and organotrophic seeding to both ocular and orbital structures [21,22,23,24]. The true incidence of orbital metastases from primary tumors may be difficult to ascertain only from clinical series of patients with histopathology reports, paving the way to a detection bias in the data collection for this paper. Cancers with more aggressive disease courses may rapidly metastasize to multiple major organs besides the orbit, severely debilitating patient functional status and thus making histological confirmation of suspected orbital metastases unnecessary. This likely explains the lower rates of primary lung cancers (5.6%) compared to other tumors such as carcinoid (6.6%). Indeed, carcinoids are less common malignancies, but they often show better prognoses and slower disease courses, with rare concurrent systemic metastases [5,8]. We also found that the median time interval between primary tumor diagnosis and the onset of orbital metastases was 12 months, further reflecting the long-lasting process of metastatic seeding into the orbit. In line with the literature on patients with choroidal metastases, the detection of orbital lesions preceded the primary tumor diagnosis in 30.1% of patients, with 37 cases of CUPs (4.2%). CUPs represent metastatic carcinomas with no primary neoplasms identified at diagnostic workup and mostly deriving from older case series [3,25,26,27,28,29]. More recently, upfront whole-body PET/CT scans are frequently recommended in patients with no history of cancer and clinical suspicion of orbital or intraocular metastases to expedite management and systemic treatments [6,30,31].

As opposed to benign orbital tumors, orbital metastases frequently manifest clinically with an abrupt onset of rapidly progressive symptoms [1,32]. We found that most metastases occurred unilaterally and diffusely infiltrated intra/extraconal orbital soft tissues, causing globe displacement with proptosis, diplopia and impaired eye motility [3,4,5]. The direct compression of the optic nerve, commonly at the orbital apex, additionally led to RAPD and vision decline, with a serious impact on patients’ functional status [33,34]. Less frequently, paradoxical enophthalmos of the affected eye resulted from the infiltration of neoplastic cells into the extraocular muscles and retro-bulbar stromal tissues causing desmoplasia, fibrosis and globe retraction [35,36,37]. Some ocular symptoms, such as visual impairments and periocular pain, may share similarities between orbital and choroidal metastases, thus requiring further assessment with orbital imaging [7,38]. This is especially the case in patients with no evident orbital or ocular masses. We noted that some primary cancers tend to infiltrate specific orbital tissues, such as breast cancers localizing within the orbital fat pad due to the local hormonal patterns, and melanomas invading and enlarging the extraocular muscles as seen clearly on MRI scans [39,40]. Similarly, prostate and liver cancers commonly infiltrate the orbital bony structures inducing osteoblastic or osteoclastic reactions, and this is better identified on CT scans [7,41].

We found that both incisional and fine-needle aspiration biopsy showed no benefits in clinical and survival outcomes but were chosen over tumor resection in most patients. On par with choroidal metastases, biopsy of suspected orbital metastases is often required for differential diagnosis in patients with no cancer history or for histomolecular characterization when biopsy of other metastatic sites is less viable [6,42]. When surgery is not deemed feasible, biopsy represents a safe alternative advantageous to initiate systemic therapy; however, tumor resection should be preferred in eligible patients. Indeed, we noted that tumor debulking, regardless of the extent-of-resection, led to significant clinical (*p* = 0.005), radiological (*p* = 0.007), and survival (*p* = 0.004) improvement when compared to biopsy. The mechanism for this result may likely be the prompt decompression with relief of mass effect and decrease in tumor burden with increased effectiveness of complementary therapies [14,39,43]. However, we note that our findings may also be related to the selection bias intrinsic in surgical series on orbital metastases because patients planning to receive surgical resection may be characterized by underlying clinical characteristics and tumor features (e.g., satisfactory performance status and single metastasis) not present in patients deemed preferable to receive less invasive and/or nonsurgical treatments.

Regarding orbital exenteration, we found no survival benefit compared to patients undergoing orbital-preserving complete tumor resection (*p* = 0.153). Such a finding substantiates once again that survival is more likely affected by systemic disease control than metastasis-directed local therapy. Orbital exenteration is often considered for treating aggressive craniofacial malignancies with orbital infiltration and perineural invasion, effectively reducing rates of local recurrences and re-operations [44,45]. In orbital metastases, the limited benefit of orbital exenteration is likely due to the underlying systemic spreading of tumor cells and related poor prognoses [46,47]. Hence, orbital exenteration may be unnecessary for treating orbital metastases in patients with systemic malignancies, and thus surgery should be intended to preserve acceptable quality of life while avoiding highly disfiguring procedures. However, orbital exenteration may play a role in providing relief of severe orbital pain in patients with already poor or absent pre-operative vision function, as we found in seven cases [3,4,39].

In some cases, orbital metastases may extend intracranially into the anterior and middle cranial fossa, similarly to other craniofacial malignancies such as nasopharyngeal carcinomas. While intracranial extension relates with poorer OS in patients with nasopharyngeal carcinomas, we found no differences in clinical (*p* = 0.582), radiological (*p* = 0.306), and survival (*p* = 0.073) outcomes between cranio-orbital and intra-orbital metastases [48]. We postulate that, contrary to primary craniofacial malignancies, both metastatic entities share similar histomolecular characteristics with comparable responsiveness to the systemic and/or radiation therapies when surgical procedures are unviable [49,50]. Still, a more in-depth evaluation of specific tumor microenvironments is highly encouraged to identify favorable therapeutic targets and support less-invasive treatment strategies specifically for patients with cranio-orbital metastases.

Radiotherapy is a well-established palliative therapeutic option for benign and malignant orbital lesions, as well as for periocular ones [11,12,32,51,52,53]. In line with previous reports on choroidal metastases, we also found that radiotherapy significantly ameliorates clinical and functional status in patients with orbital metastases (*p* = 0.032), favoring nonsurgical lesion shrinkage and relief of mass effect [22,54]. Although some cases of radiation-induced cataracts have been reported in earlier studies [55], modern image-guided radiotherapy planning allows the delivery of maximal doses to selected targets [56,57], sparing critical orbital structures and preventing the onset of severe adverse events [55,58,59,60]. Thus, radiotherapy appears to be safe and effective in the treatment of orbital metastases similarly to choroidal metastases, but the severity of radiation-induced complications might differ due to the unfortunate proximity of choroidal metastases to the macula and lens [54,61]. The usefulness of particle therapy, a type of radiotherapy characterized by a peculiarly beneficial dose distribution to the target (Bragg peak), to maximize the sparing of critical ocular structures with respect to the photon-based radiotherapy needs to be further investigated to evaluate its cost effectiveness [62,63]. However, particle therapy has not a widespread distribution, and classic radiotherapy could be more easily accessible. In such critical anatomical sites, safely delivering a high ablative dose in those tumor layers more distantly located from organs at risk while gradually underdosing the successive ones might have a radiobiological rationale in addition to a potentially successful effect. This approach with partial tumor irradiation or with spatially fractionation of the radiation dose is just a hypothesis because it has been effectively tested only for treatment of bulky tumors at different body sites from those examined here [64,65].

While chemotherapy, immunotherapy, and hormonal therapies (for breast and prostate cancers) proved to be significantly effective in concurrently treating both primary tumors and choroidal metastases, their impact on orbital metastases requires further analysis [66,67,68,69]. We found no significant correlation between systemic therapies and clinical (*p* = 0.247) or survival (*p* = 0.361) response, perhaps due to the different entry routes of medications into orbital tumors as compared to choroidal tumors [1,28].

The prognosis of patients with orbital metastases is poor with one-year survival rates, (40%) lower than patients with choroidal metastases (52%) and comparable to patients with brain metastases (39–46%), implying that their occurrence is more frequent at the very advanced disease stages, which call for an effective improvement in current systemic therapeutic strategies [22,50,70,71]. In line with previous reports, we found significant survival differences amongst primary tumors (*p* < 0.001), with one-year survival rates highest in breast cancer (57%) and carcinoid (53%), and lowest in lung (14%), liver (27%), and melanoma (29%) cancers, likely attributed to their underlying aggressiveness and responsiveness to treatments [3,5,8,9,39]. Hence, our findings further substantiate that the current treatments for orbital metastases are palliative and should be aimed at improving symptoms while preserving patient quality of life.

### Limitations

Our study has specific limitations. All included articles were retrospective case reports and case series exposed to selection bias, and they comprised a 56-year time-period characterized by major advances in surgical, radiotherapy and systemic oncological treatments, thus likely introducing a chronological bias into our analysis. As mentioned above, our study design involves only histologically confirmed orbital metastases: the exclusion from this data collection of the biopsy-unproven ones could lead to an underestimation of their overall prevalence as well as of their treatment-related outcomes (detection bias). However, our selective inclusion criteria were set to minimize the risks of introducing possible confounding variables related to misdiagnoses of orbital metastases only suspected at clinical and radiological assessments. Indeed, the nature of this systematic review—coupled with the lack of detailed information on criteria used to diagnose suspected orbital metastases among the vast majority of studies found in the literature—prevented us from calculating and adjusting the between-studies inter-observer variability in clinical and/or radiological diagnosis using appropriate statistical tests. The assessment of ocular symptoms was subjective in most studies, based on patient reports. Indeed, we are not able to fully explain why the improvement in symptoms does not correlate with the extent of surgical resection. Presumably, this finding could be affected by an apprehension bias if the risk for disabling and disfiguring consequences discourages patients from submitting themselves to more demolitive surgical procedures. The role of higher aggressive vs. lower palliative radiation doses could not be evaluated due to the lack of granular data across included articles. Due to the commonly missing data in the literature, we could not comprehensively analyze post-surgery adverse events, the impact of tumor size and concurrent systemic metastases on patient survival, or the role of advanced immune/targeted therapies. These confounding variables, coupled with missing information on baseline patients’ performance statuses and the likely selection bias related to our inclusion criteria, may have also limited our survival analysis based on surgery vs. biopsy.

## 5. Conclusions

Orbital metastases are rare, debilitating lesions in oncological patients. Histopathological examination is recommended to guide complementary therapeutic protocols, but surgery is often challenging. Tumor resection, regardless of its extent, showed improved clinical and survival outcomes over biopsy, but the impact of underlying clinical and tumor characteristics should be still considered on a case-by-case basis before planning surgical strategies. Orbital exenteration appears less useful when used in addition to complete tumor resection because no survival benefit was found. Furthermore, the positive clinical impact of orbital radiotherapy may favor its implementation in patients not eligible to undergo surgery or who underwent a subtotal resection. Future prospective studies are required to better understand the role of multimodal systemic therapeutic strategies in the management of orbital metastases based on primary tumor histopathology.

## Figures and Tables

**Table 1:** Summary of clinical and anatomical characteristics of all pooled patients.

Characteristics	Value
Cohort size (no.)	873
Demographics	
Age (years), median (range) (*n* = 683)	59, 18–90
Gender (female) (*n* = 868)	492 (56.7%)
Primary Tumor Sites (*n* = 873)	No. (%)
Breast	317 (36.3%)
Melanoma	88 (10.1%)
Prostate	74 (8.5%)
Carcinoid	58 (6.6%)
Lung	49 (5.6%)
Carcinoma of Unknown Primary (CUP)	37 (4.2%)
Kidney	34 (3.9%)
Liver	30 (3.4%)
Bone	27 (3.1%)
Soft Tissue	20 (2.3%)
Salivary Gland	16 (1.8%)
Small Intestine	16 (1.8%)
Skin (not melanoma)	15 (1.7%)
Colorectal	14 (1.6%)
Thyroid	13 (1.5%)
Bladder	12 (1.4%)
Others	53 (6.1%)
Clinical Presentation (*n* = 705)	
Orbital metastases preceding primary, no. (%)	218 (30.1%)
Time interval from primary (months)	
Median (range)	12 (0–420)
Mean (SD)	40.9 (± 6.4)
Laterality (*n* = 744)	No. (%)
Left	344 (46.2%)
Right	343 (46.1%)
Bilateral	57 (7.7%)
Location (*n* = 443)	No. (%)
Superior	59 (13.3%)
Superolateral	64 (14.4%)
Superomedial	22 (5%)
Lateral	59 (13.3%)
Medial	69 (15.6%)
Inferior	20 (4.5%)
Inferolateral	5 (1.1%)
Inferomedial	7 (1.6%)
Posterior	54 (12.2%)
Posterior	84 (19%)
Tissue Infiltrated (*n* = 545)	No. (%)
Soft Tissue	219 (40.2%)
Muscle	146 (26.8%)
Bone	114 (20.9%)
Fat	66 (12.1%)
Intracranial Extension (*n* = 299)	47 (15.7%)
Presenting Symptoms (*n* = 848)	No. (%)
Proptosis	444 (52.3%)
Relative afferent pupillary defect (RAPD)	328 (38.7%)
Diplopia	301 (35.5%)
Impaired eye motility	247 (29.1%)
Palpable/visible mass	183 (21.6%)
Orbital Pain	163 (19.2%)
Blurred/decreased vision	159 (18.7%)
Ptosis	133 (15.7%)
Swelling	108 (12.7%)
Vision Loss	65 (7.7%)
Enophthalmos	39 (4.6%)
Red eye	38 (4.5%)
No Symptoms	5 (0.6%)
Radiological Appearance (*n* = 759)	No. (%)
Osteolytic	112 (14.7%)
Osteoblastic	10 (1.3%)

**Table 2:** Summary of management strategies of all pooled patients.

Characteristics	Value
Surgery (*n* = 873)	No. (%)
Incisional biopsy	556 (63.7%)
Fine needle aspiration biopsy (FNAB)	89 (10.2%)
Partial Resection (<100%)	145 (16.6%)
Complete Resection (100%)	83 (9.5%)
Orbital exenteration	26/83 (31.3%)
Chemotherapy (*n* = 751)	305 (40.6%)
Radiotherapy (*n* = 751)	506 (67.4%)
Dose (Gy), median (range)	35.5 (10.0–60.0)
Additional Treatments (*n* = 151)	No. (%)
Hormonal therapy	128 (84.8%)
Immunotherapy	12 (7.9%)
Steroids	7 (4.6%)
Radioiodine therapy	4 (2.6%)
Clinical Response (*n* = 315)	No. (%)
Symptom improvement	203 (64.4%)
Radiological Response (*n* = 287)	No. (%)
Complete Response (CR)	52 (18.1%)
Partial Response (PR)	143 (49.9%)
Stable Disease (SD)	18 (6.3%)
Progression (PD)	74 (25.8%)
Follow-up (months), mean (range)	14.3 (0.2–144.0)
Recurrence of Orbital Metastases	15 (1.7%)
Survival (months) (*n* = 873)	No. (%)
Local Control	
Median (range)	6.0 (0.2–120.0)
Mean (SD)	13.8 (± 3.8)
Overall Survival	
Median (range)	6.0 (0.2–144.0)
Mean (SD)	15.3 (± 3.9)
Status (*n* = 641)	No. (%)
Alive	225 (35.1%)
Dead	416 (64.9%)

**Table 3:** Univariate and multivariate logistic regression analyses predicting patients’ post-treatment symptom improvement in patients with orbital metastases.

Characteristics	No. (%)	Univariate Analysis			Multivariate Analysis		
		OR	95% CI	*p*-Value *	OR	95% CI	*p*-Value *
Age		1.012	0.994–1.030	0.200			
Gender							
Male	117 (47.8%)	Ref					
Female	128 (52.2%)	1.115	0.688–1.938	0.586			
Primary Tumor							
Breast	60 (35.7%)	Ref					
Melanoma	8 (4.8%)	0.395	0.089–1.764	0.224			
Prostate	27 (16.1%)	1.740	0.567–5.340	0.333			
Carcinoid	16 (9.5%)	0.659	0.207–2.097	0.480			
Lung	8 (4.8%)	0.395	0.089–1.764	0.224			
Kidney	13 (7.7%)	0.890	0.241–3.280	0.860			
Liver	22 (13.1%)	0.395	0.144–1.082	0.071			
Soft Tissue	14 (8.3%)	0.527	0.159–1.747	0.295			
Laterality							
Right	109 (44.5%)	Ref					
Left	117 (47.8%)	0.998	0.581–1.714	0.993			
Bilateral	19 (7.7%)	0.644	0.241–1.718	0.380			
Orbital Location							
Superior	36 (14.8%)	Ref					
Superolateral	50 (20.6%)	1.837	0.743–4.543	0.188			
Superomedial	17 (7%)	1.020	0.316–3.292	0.973			
Lateral	32 (13.2%)	0.630	0.241–1.646	0.346			
Medial	34 (14%)	1.714	0.636–4.621	0.287			
Inferior	15 (6.2%)	1.429	0.405–5.044	0.579			
Inferolateral	3 (1.2%)	1.429	0.118–7.234	0.779			
Inferomedial	6 (2.5%)	1.341	0.107–6.067	0.890			
Posterior	25 (10.3%)	0.561	0.200–1.573	0.272			
Diffuse	25 (10.3%)	1.518	0.521–4.426	0.445			
Tissue Infiltrated							
Bone	52 (21.6%)	Ref					
Muscle	74 (30.7%)	0.603	0.288–1.263	0.180			
Soft Tissue	93 (38.6%)	0.805	0.394–1.646	0.552			
Fat	22 (9.1%)	1.651	0.521–5.233	0.394			
Intracranial Extension							
Yes	39 (15.7%)	Ref					
No	198 (84.3%)	1.217	0.604–2.453	0.582			
Radiological Features							
Osteolytic	43 (84.3%)	Ref					
Osteoblastic	8 (15.7%)	2.710	0.301–4.423	0.374			
Biopsy vs. Surgery							
Biopsy	152 (62%)	Ref			Ref		
Surgery	93 (38%)	2.144	1.226–3.750	**0.008**	1.985	1.096–3.100	**0.005**
Biopsy Approach							
Incisional	131 (85.6%)	Ref					
FNAB	22 (14.4%)	1.148	0.459–2.872	0.769			
Extent of Resection							
Complete	54 (58.7%)	Ref					
Partial	38 (41.3%)	1.723	0.654–4.538	0.271			
Exenteration							
Yes	7 (26.7%)	Ref					
No	30 (73.3%)	1.600	0.247–10.360	0.622			
Chemotherapy							
No	158 (61%)	Ref					
Yes	85 (39%)	1.386	0.798–2.409	0.247			
Radiotherapy							
No	113 (46.5%)	Ref			Ref		
Yes	130 (53.5%)	1.890	1.232–2.871	**0.047**	1.311	1.066–2.127	**0.032**

**Abbreviations**: OR, Odds ratio; CI, Confidence Interval; Ref, Reference group; FNAB, Fine needle aspiration biopsy. * Two-tailed *p*-value < 0.05 was considered statistically significant for all tests. In **bold** are statistically significant results. For each variable, numbers may not sum up to the total number of patients with available data on clinical improvement (*n* = 315) due to the limited granular data found across included articles.

**Table 4:** Univariate and multivariate logistic regression analyses predicting patients’ post-treatment radiological tumor volume reduction in patients with orbital metastases.

Characteristics	No. (%)	Univariate Analysis			Multivariate Analysis		
		OR	95% CI	*p*-Value *	OR	95% CI	*p*-Value *
Age		0.992	0.973–1.011	0.411			
Gender							
Male	107 (49.3%)	Ref					
Female	110 (50.7%)	0.959	0.545–1.688	0.885			
Primary Tumor							
Breast	61 (38.6%)	Ref			Ref		
Melanoma	17 (10.8%)	0.290	0.095–0.885	**0.030**	0.244	0.076–0.783	**0.018**
Prostate	26 (16.4%)	2.500	0.657–9.517	0.179	1.768	0.443–7.049	0.420
Carcinoid	12 (7.6%)	0.652	0.172–2.476	0.503	0.718	0.179–2.881	0.640
Lung	5 (3.2%)	0.489	0.075–3.211	0.456	0.350	0.051–2.394	0.284
Kidney	10 (6.3%)	0.489	0.121–1.970	0.314	0.402	0.095–1.702	0.216
Liver	16 (10.1%)	0.419	0.133–1.320	0.137	0.323	0.097–1.070	0.064
Soft Tissue	11 (7%)	0.391	0.104–1.468	0.164	0.393	0.100–1.550	0.182
Laterality							
Right	96 (44.2%)	Ref			Ref		
Left	102 (47%)	0.749	0.409–1.371	0.349	0.628	0.284–1.390	0.251
Bilateral	19 (8.8%)	0.352	0.129–0.962	**0.042**	0.217	0.065–0.728	**0.013**
Orbital Location							
Superior	29 (14.8%)	Ref					
Superolateral	40 (20.4%)	1.388	0.494–3.900	0.534			
Superomedial	12 (6.1%)	0.526	0.134–2.064	0.357			
Lateral	27 (13.8%)	0.489	0.167–1.432	0.192			
Medial	28 (14.3%)	1.930	0.591–6.304	0.276			
Inferior	14 (7.1%)	1.930	0.436–8.551	0.387			
Inferolateral	2 (1%)	0.526	0.030–9.335	0.662			
Inferomedial	4 (2%)	1.097	0.204–5.623	0.790			
Posterior	18 (9.2%)	0.827	0.245–2.797	0.760			
Diffuse	22 (11.2%)	0.526	0.169–1.635	0.267			
Tissue Infiltrated							
Bone	42 (21.6%)	Ref					
Muscle	63 (32.3%)	1.469	0.624–3.348	0.379			
Soft Tissue	76 (39%)	0.618	0.282–1.354	0.229			
Fat	14 (7.2%)	1.250	0.332–4.704	0.741			
Intracranial Extension							
Yes	29 (16%)	Ref					
No	152 (84%)	1.529	0.670–3.453	0.306			
Radiological Features							
Osteolytic	34 (82.9%)	Ref					
Osteoblastic	7 (17.1%)	2.160	0.228–20.492	0.502			
Biopsy vs. Surgery							
Biopsy	154 (68.1%)	Ref					
Surgery	72 (31.9%)	1.679	0.872–3.233	0.121			
Biopsy Approach							
Incisional	135 (87.1%)	Ref					
FNAB	20 (12.9%)	0.828	0.316–2.165	0.700			
Extent of Resection							
Complete	38 (61.5%)	Ref					
Partial	24 (38.5%)	1.548	0.462–5.187	0.479			
Exenteration							
Yes	3 (13%)	Ref					
No	20 (87%)	1.742	0.870–4.874	0.369			
Chemotherapy							
No	129 (40.3%)	Ref					
Yes	87 (59.7%)	0.627	0.353–1.115	0.112			
Radiotherapy							
No	77 (35.6%)	Ref					
Yes	139 (64.4%)	1.760	0.982–3.154	0.057			

**Abbreviations**: OR, Odds ratio; CI, Confidence Interval; Ref, Reference group; FNAB, Fine needle aspiration biopsy. * Two-tailed *p*-value < 0.05 was considered statistically significant for all tests. In **bold** are statistically significant results. For each variable, numbers may not sum up to the total number of patients with available data on post-treatment radiological tumor volume (*n* = 287) due to the limited granular data found across included articles.

**Table 5:** Univariate and multivariate adjusted Cox proportional hazards analyses of variables associated with overall survival among patients with orbital metastases.

Characteristics	No. (%)	Univariate Analysis			Multivariate Analysis		
		HR	95% CI	*p*-Value *	HR	95% CI	*p*-Value *
Age		1.004	0.994–1.014	0.426			
Gender							
Male	229 (53.8%)	Ref					
Female	197 (46.2%)	1.286	0.990–1.671	0.060			
Primary Tumor							
Breast	127 (38.7%)	Ref			Ref		
Melanoma	46 (14%)	1.893	1.257–2.851	**0.002**	2.047	1.353–3.096	**0.001**
Prostate	40 (12.2%)	1.462	0.896–2.387	0.128	1.493	0.915–2.438	0.109
Carcinoid	31 (9.4%)	0.722	0.391–1.336	0.300	0.883	0.468–1.666	0.700
Lung	19 (5.8%)	3.593	1.989–6.488	**<0.001**	3.859	2.130–6.993	**<0.001**
Kidney	17 (5.2%)	1.074	0.430–2.682	0.878	1.494	0.571–3.908	0.413
Liver	29 (8.8%)	3.052	1.777–5.240	**<0.001**	3.249	1.888–5.590	**<0.001**
Soft Tissue	19 (5.8%)	1.635	0.808–3.308	0.172	2.065	0.992–4.302	0.053
Laterality							
Right	182 (44.4%)	Ref					
Left	191 (46.6%)	0.983	0.739–1.306	0.904			
Bilateral	37 (9%)	1.136	0.720–1.792	0.584			
Orbital Location							
Superior	44 (13.5%)	Ref					
Superolateral	64 (19.6%)	1.193	0.675–2.108	0.543			
Superomedial	22 (6.7%)	0.711	0.317–1.591	0.406			
Lateral	41 (12.6%)	1.102	0.587–2.069	0.763			
Medial	45 (13.8%)	0.646	0.344–1.212	0.174			
Inferior	17 (5.2%)	0.760	0.324–1.781	0.528			
Inferolateral	5 (1.5%)	1.075	0.321–3.603	0.907			
Inferomedial	7 (2.1%)	0.175	0.024–1.300	0.089			
Posterior	43 (13.1%)	1.249	0.712–2.191	0.438			
Diffuse	39 (11.9%)	1.253	0.688–2.280	0.461			
Tissue Infiltrated							
Bone	77 (22.5%)	Ref					
Muscle	106 (31%)	0.786	0.511–1.210	0.274			
Soft Tissue	127 (37.1%)	0.928	0.674–1.541	0.928			
Fat	32 (9.4%)	0.961	0.531–1.738	0.896			
Intracranial Extension							
Yes	47 (15.7%)	Ref					
No	252 (84.3%)	0.646	0.394–1.059	0.083			
Radiological Features							
Osteolytic	54 (84.4%)	Ref					
Osteoblastic	10 (15.6%)	0.559	0.209–1.496	0.275			
Biopsy vs. Surgery							
Biopsy	297 (68.7%)	Ref			Ref		
Surgery	135 (31.3%)	0.642	0.475–0.868	**0.004**	0.649	0.441–0.955	**0.028**
Biopsy Approach							
Incisional	262 (87.9%)	Ref					
FNAB	36 (12.1%)	1.234	0.789–1.933	0.357			
Extent of Resection							
Complete	61 (45.5%)	Ref					
Partial	73 (54.5%)	1.232	0.725–2.094	0.440			
Exenteration							
Yes	16 (26.7%)	Ref					
No	44 (73.3%)	1.926	0.757–4.904	0.169			
Chemotherapy							
No	229 (61%)	Ref					
Yes	147 (39%)	0.874	0.654–1.167	0.361			
Radiotherapy							
No	176 (46.8%)	Ref					
Yes	200 (53.2%)	0.820	0.618–1.088	0.170			

**Abbreviations**: HR, Hazard ratio; CI, Confidence Interval; Ref, Reference group; FNAB, Fine needle aspiration biopsy. * Two-tailed *p*-value < 0.05 was considered statistically significant for all tests. In **bold** are statistically significant results. For each variable, numbers may not sum up to the total number of patients with available survival data (*n* = 641) due to the limited granular data found across included articles.

## Supplementary Materials

The following are available online at https://www.mdpi.com/article/10.3390/cancers14010094/s1, Table S1: Overview and references of all included studies, Table S2: Risk of bias assessments for all included studies.
