# Supplementary material for: Orbital Metastases: A Systematic Review of Clinical Characteristics, Management Strategies, and Treatment Outcomes
Source: Cancers (Basel). 2021 Dec 24;14(1):94. doi: 10.3390/cancers14010094 (PMC8750198; doi:10.3390/cancers14010094)
Supplement: Supplementary file 1 [file cancers-14-00094-s001.zip › Supplementary/Table S1 - Overview.pdf]

Table S1. Overview of all included studies.

Author – Year	Study design-Level of evidence	Patients No./Female	Age years median (range)	Primary Tumors No. patients (percentage)	Interval between Primary & metastases months, median (range)	Laterality No. patients (percentage)	Localization within the Orbit No. patients (percentage)	Tissue infiltrated No. patients (percentage)	Intracranial extension No. patients (percentage)	Symptoms No. patients (percentage)	Imaging features No. patients (percentage)	Extent of surgery No. patients (percentage)	Surgical technique No. patients (percentage)	Chemo therapy No. patients (percentage)	Orbital Radio therapy No. patients (percentage) median (Gy)	Other therapy No. patients (percentage)	Clinical Improve-ment No. patients (percentage)	RANO response No. patients (percentage)	Recurrence orbital metastases No. patients (percentage)	PFS months, median (range)	OS months, median (range)	Alive No. patients (percentage)
**Arnott et al. 1965**[1]	Case Report – V	1 / 1	64	Breast	0	R	Superior	Soft Tissue	0	Mass, Pt	N/A	Biopsy	N/A	0	1	Hormonal	No	PD	0	2.0	2.0	1
**Chatterjee et al. 1965**[2]	Case Report – V	1 / 0	40	CUP	0	L	Superior	Soft Tissue	0	D, Pro, Pt, RE, S	N/A	Complete	Exenteration	0	0	0	Yes	N/A	0	1.0	1.0	0
**Mortada et al. 1968**[3]	Case Series – IV	10 / 5 (50%)	46.5 (25-55)	3 Breast (30%),<br>2 Skin (20%),<br>1 (10%) Liver, Lung, Muscle, Prostate, Thyroid	12.0 (6-24)	7 R (70%),<br>3 L (30%)	6 Superior (60%)<br>2 Posterior (20%)<br>1 Medial (10%)<br>1 Inferior (10%)	7 Muscle (70%)<br>2 Soft Tissue (20%)<br>1 Bone (10%)	0	10 Pro (100%)	N/A	10 Biopsy (100%)	N/A	N/A	10 (100%)	0	0	10 PR (100%)	0	12.0 (6-36)	12.0 (6-36)	0
**Honrubia et al. 1971**[4]	Case Report – V	1 / 1	74	Carcinoid	48.0	B	Inferolateral	Soft Tissue	0	D, Mass	N/A	Biopsy	N/A	0	0	0	0	N/A	0	24.0	24.0	1
**Ashton et al. 1974**[5]	Case Series – IV	2 / 2	53.5 (49-58)	2 Breast (100%)	0.0	2 R (100%)	1 Posterior (10%)<br>1 Superior (10%)	1 Muscle (10%)<br>1 Soft Tissue (10%)	0	2 Pt (100%),<br>1 S (50%)	N/A	1 Biopsy (50%)<br>1 Partial (50%)	N/A	0	0	0	N/A	N/A	0	54.0 (30-78)	54.0 (30-78)	0
**Huh et al. 1974**[6]	Case Series – IV	70 / 50 (71.4%)	51	28 Breast (40%)<br>23 Bone (32.8%)<br>5 Melanoma (7.1%)<br>3 Lung (4.3%)<br>3 Prostate (4.3%)<br>3 Salivary (4.3%)<br>2 CUP (2.8%)<br>1 Colon (1.4%)<br>1 Thyroid (1.4%)<br>1 Tongue (1.4%)	58.0	9 B (12.8%)	N/A	N/A	N/A	HA, ICH, MI, SpI	12 Osteolytic (17.1%)	70 Biopsy (100%)	N/A	0	70 (100%)	0	50 (71.4%)	N/A	0	6.0 (1-60)	6.0 (1-60)	4 (5.7%)
**Saxena et al. 1975**[7]	Case Report – V	1 / 1	60	Salivary Gland	7.0	R	Posterior	Muscle	0	DV, Pro	N/A	Complete	Exenteration	0	0	0	0	N/A	1	12.0	18.0	1
**Usui et al. 1975**[8]	Case Report – V	1 / 0	79	Prostate	0	L	N/A	N/A	0	D, Pro	N/A	Biopsy	N/A	0	0	Hormonal	1	N/A	0	5.0	5.0	0
**Balestrazzi et al. 1976**[9]	Case Report – V	1 / 0	59	Melanoma	35.0	R	Medial	Soft Tissue	0	Mass	N/A	Complete	N/A	1	0	0	1	CR	0	72.0	72.0	1
**Howard et al. 1978**[10]	Case Report – V	1 / 0	47	Kidney	0	R	Superolateral	Bone	0	D, IM, Mass, Pro, S	Osteolytic	Partial	N/A	0	1, 35 Gy	Immunotherapy	1	CR	0	9.0	9.0	1
**Stewart et al. 1978**[11]	Case Report – V	1 / 0	47	Soft Tissue	144.0	L	Lateral	Soft Tissue	0	D, IM, OP	N/A	Partial	N/A	1	0	0	0	PD	0	6.0	6.0	1
**Bullock et al. 1980**[12]	Case Report – V	1 / 1	69	Breast	84.0	R	Inferomedial	Soft Tissue	0	DV, IM	N/A	Biopsy	N/A	0	1	Hormonal	1	PR	0	3.0	3.0	1
**Lubin et al. 1980**[13]	Case Report – V	1 / 0	69	Liver	0	R	Superolateral	Soft Tissue	0	Pro	N/A	Biopsy	N/A	0	1, 30 Gy	0	1	PR	0	1.0	1.0	1
**Rush et al. 1980**[14]	Case Report – V	1 / 1	58	Colorectal	1.0	L	Lateral	Soft Tissue	0	IM, Pro	N/A	Biopsy	N/A	0	1, 35 Gy	0	1	PR	0	1.0	1.0	1
**Sobol et al. 1980**[15]	Case Report – V	1 / 0	48	Melanoma	72.0	R	Inferolateral	Soft Tissue	0	DV, IM, Pro	N/A	Partial	N/A	0	0	0	1	N/A	0	6.0	6.0	0
**Rush et al. 1981**[16]	Case Report – V	1 / 0	28	Testes	27.0	L	Posterior	Soft Tissue	1	DV, OP, Pro	N/A	Biopsy	Decompression	0	1, 30 Gy	0	1	PR	0	1.0	1.0	1
**Winkler et al. 1981**[17]	Case Report – V	1 / 0	59	Prostate	0	L	Posterior	Soft Tissue	1	IM, Pro, RAPD, VL	Osteolytic	Biopsy	Decompression	0	0	0	1	N/A	0	2.0	2.0	1
**Wolter et al. 1981**[18]	Case Report – V	1 / 0	77	Prostate	60.0	R	Medial	Bone	0	D, DV, IM, Pro	Osteoblastic	Partial	N/A	0	1	Hormonal	1	N/A	0	21.0	21.0	1
**Zubler et al. 1981**[19]	Case Report – V	1 / 0	64	Liver	0	L	Posterior	Soft Tissue	1	Mass, Pro, VL	Osteolytic	Biopsy	N/A	1	1, 40 Gy	0	0	PR	0	3.0	3.0	0

**Carriere et al. 1982**[20]	Case Report – V	1 / 0	79	Prostate	0	B	Diffuse	Soft Tissue	0	DV, IM, Pro, RAPD	N/A	Biopsy	N/A	0	0	Hormonal	0	N/A	0	1.0	1.0	1
**Riddle et al. 1982**[21]	Case Series – IV	7 /<br>4 (57.1%)	66<br>(51-71)	7 Carcinoid (100%)	18.0<br>(12-60)	N/A	N/A	N/A	N/A	5 Pro (71.4%),<br>1 D (14.3%),<br>1 Mass (14.3%)	N/A	3 Biopsy (42.9%)<br>3 Complete (42.9%)<br>1 Partial (14.3%)	3 Exenteration (42.9%)	0	0	0	N/A	N/A	0	48.0<br>(4-108)	48.0<br>(4-108)	1 (14.3%)
**Tertzakian et al. 1982**[22]	Case Series – IV	2 /<br>0	64<br>(62-66)	2 Prostate (100%)	15.5<br>(0-31)	1 L (50%)<br>1 R (50%)	1 Lateral (50%)<br>1 Superior (100%)	2 Bone (100%)	N/A	1 D, Pro (50%)<br>1 No sympt (50%)	N/A	1 FNAB (50%)<br>1 Biopsy (50%)	N/A	1 (50%)	1 (50%)	1 Hormonal (50%)	0	PD	0	6.0<br>(3-9)	6.0<br>(3-9)	1 (50%)
**Weitberg et al. 1983**[23]	Case Report – V	1 / 1	55	CUP	0	L	Medial	Bone	0	D, IM, Mass	N/A	Biopsy	N/A	1	1, 40 Gy	0	1	PR	0	12.0	12.0	1
**Mortada et al. 1984**[24]	Case Series – IV	3 /<br>3 (100%)	55<br>(53-56)	3 Breast (100%)	72.0<br>(60-84)	2 L (66.7%)<br>1 R (33.3%)	1 Medial (33.3%)<br>1 Lateral (33.3%)<br>1 Superior (33.3%)	3 Muscle (100%)	0	3 D (100%)<br>3 DV (100%)	N/A	3 Biopsy (100%)	N/A	3 (100%)	3, 40 Gy	0	N/A	N/A	0	36.0	36.0	3 (100%)
**Reifler et al. 1984**[25]	Case Report – V	1 / 0	74	Prostate	13.0	R	Superior	Muscle	0	DV, IM	N/A	FNAB	N/A	0	1, 36 Gy	Hormonal	1	PR	0	1.0	1.0	1
**Dunaway et al. 1985**[26]	Case Report – V	1 / 0	60	Lung	7.0	L	Superolateral	Bone	0	D, DV, Pt	Osteolytic	Biopsy	N/A	1	1	0	0	PD	0	12.0	12.0	1
**Bond et al. 1986**[27]	Case Series – IV	2 /<br>1 (50%)	42<br>(33-51)	2 Melanoma (100%)	96.0<br>(84-108)	1 L (50%)<br>1 R (50%)	1 Inferolateral (50%)<br>1 Medial (50%)	2 Bone (100%)<br>1 Muscle (50%)	0	2 S (100%)<br>1 Mass, Pro (50%)<br>1 D, OP (50%)	N/A	2 Partial (100%)	2Decompression (100%)	2 (100%)	2 (100%)	0	N/A	1 CR (50%)<br>1 PD (50%)	0	23.0<br>(10-36)	23.0<br>(10-36)	0
**Denby et al. 1986**[28]	Case Report – V	1 / 0	65	Kidney	0	T	Lateral	Bone	0	D, S	Osteolytic	Partial	N/A	0	0	0	1	CR	0	12.0	12.0	1
**Schlaen et al. 1986**[29]	Case Report – V	1 / 0	58	Breast	120.0	L	Diffuse	Soft Tissue	0	D, Mass, Pro, RAPD	N/A	Partial	N/A	1	0	0	0	PD	0	1.0	1.0	0
**Hornblass et al. 1987**[30]	Case Report – V	1 / 1	35	Thyroid	0	R	Superolateral	Bone	0	Mass, S	Osteolytic	Partial	N/A	1	1	0	N/A	N/A	0	1.0	1.0	1
**Kaltreider et al. 1987**[31]	Case Report – V	1 / 1	43	Soft Tissue	83.0	R	Medial	Bone	0	DV, IM, Pro	Osteolytic	Complete	N/A	1	0	0	1	CR	0	7.0	7.0	1
**Noci et al. 1987**[32]	Case Report – V	1 / 1	54	Breast	108.0	L	Superolateral	Soft Tissue	0	Pro	N/A	Biopsy	N/A	0	0	0	0	N/A	0	12.0	12.0	0
**Oosterhuis et al. 1987**[33]	Case Report – V	1 / 0	35	Melanoma	12.0	R	Lateral	Muscle	0	D, DV, Pro	N/A	Partial	N/A	0	0	0	1	PR	1	9.0	9.0	0
**Otto et al. 1987**[34]	Case Report – V	1 / 0	41	Skin	108.0	L	Posterior	Soft Tissue	0	Mass, OP	N/A	Complete	Exenteration	1	1, 50 Gy	0	0	PR	0	48.0	48.0	0
**Shields et al. 1987**[35]	Case Report – V	1 / 1	63	Carcinoid	84.0	R	Superior	Muscle	0	D, OP, Pro	N/A	Complete	N/A	0	0	0	0	N/A	0	1.0	1.0	1
**Stefanyszyn et al. 1987**[36]	Case Series – IV	4 /<br>4 (100%)	50<br>(47-70)	4 Breast (100%)	48<br>(0-60)	2 L (50%)<br>2 R (50%)	2 Superior (50%)<br>1 Inferior (25%)<br>1 Superolateral (25%)	2 Soft Tissue (50%)<br>1 Bone (25%)<br>1 Muscle (25%)	0	3 En (75%)<br>3 Pro (75%)<br>1 IM (25%)	N/A	4 Biopsy (100%)	N/A	1 (25%)	4 (100%)	1 Hormonal (25%)	4 (100%)	4 PR (100%)	0	30.0<br>(3-36)	30.0<br>(3-36)	4 (100%)
**Boldt et al. 1988**[37]	Case Series – IV	8 /<br>0	71.5<br>(58-84)	8 Prostate (100%)	N/A	7 R (87.5%)<br>1 L (12.5%)	5 Superior (62.5%)<br>2 Superolateral (25%)<br>1 Posterior (12.5%)	6 Bone (75%)<br>2 Soft Tissue (25%)	0	6 D (85%),<br>6 Pro (75%),<br>4 OP (50%),<br>3 S (37.5%),<br>2 DV (25%),<br>2 Pt (25%),<br>1 Mass (12.5%)<br>1 RE (12.5%)	4Osteoblastic (50%)<br>1 Osteolytic (12.5%)	7 Biopsy (87.5%),<br>1 Complete (12.5%)	N/A	1 (12.5%)	5 (62.5%), 30 Gy	0	7 (87.5%)	2 CR (25%)<br>5 PR (62.5%)<br>1 PD (12.5%)	1 (12.5%)	6.5<br>(3-60)	6.5<br>(3-60)	2 (25%)
**Orcutt et al. 1988**[38]	Case Series – IV	6 /<br>2 (33.3%)	55<br>(49-87)	6 Melanoma (100%)	36.0<br>(12-60)	4 L (66.7%)<br>2 R (33.3%)	2 Medial (33.3%)<br>1 Diffuse (16.7%)<br>1 Lateral (16.7%)<br>1 Posterior (16.7%)<br>1 Superior (16.7%)	3 Muscle (50%)<br>2 Soft Tissue (33.3%)<br>1 Bone (16.7%)	0	5 Pro (83.3%),<br>3 D (50%),<br>1 IM (16.7%),<br>1 OP (16.7%),<br>1 RE (16.7%),	N/A	3 Complete (50%)<br>1 Biopsy (33.3%)<br>1 FNAB	N/A	1 (16.7%)	4 (66.7%), 36 Gy	0	N/A	N/A	0	3.0<br>(2-6)	3.0<br>(2-6)	0

										1 S (16.7%),<br>1 VL (16.7%)		(16.7%)										
**Jacobs et al. 1988**[39]	Case Series – IV	2 / 0	58.5 (56-61)	2 Breast (100%)	84.0 (72-96)	2 R (100%)	2 Superolateral (100%)	2 Soft Tissue (100%)	0	2 Pro (100%)	N/A	2 Biopsy (100%)	N/A	1 (50%)	1 (50%)	2 Hormonal (100%)	2 (100%)	1 PR (50%)<br>1 SD (50%)	0	14.0 (12-16)	14.0 (12-16)	2 (100%)
**Shields et al. 1988**[40]	Case Series – IV	35 / 21 (60%)	70	18 Breast (51.4%)<br>6 Prostate (17.1%)<br>2 CUP (5.7%)<br>2 Lung (5.7%)<br>2 Melanoma (5.7%)<br>1 Carcinoid (2.9%)<br>1 Kidney (2.9%)	N/A	21 R (60%)<br>12 L (34.3%)<br>2 B (5.7%)	N/A	N/A	0	18 D (51.4%)<br>17 Pro (48.6%)<br>7 Mass (20%)<br>7 OP (20%)<br>6 RE (17.1%)<br>6 VL (17.1%)<br>5 RAPD (14.3%)<br>2 S (5.7%)	N/A	35 Biopsy (100%)	N/A	N/A	N/A	N/A	N/A	N/A	0	13.0	13.0	N/A
**Malviya et al. 1989**[41]	Case Report – V	1 / 1	66	Ovary	0	L	Posterior	Fat	0	VL	0	Complete	N/A	1	0	0	0	PR	0	3.0	3.0	1
**Ruusuvaara et al. 1989**[42]	Case Report – V	1 / 0	63	Melanoma	53.0	R	Posterior	Soft Tissue	0	IM, Pro	N/A	FNAB	N/A	1	0	0	0	PD	0	1.0	1.0	0
**Spaide et al. 1989**[43]	Case Report – V	1 / 0	61	Lung	0	R	Superolateral	Soft Tissue	0	Mass, Pro, RAPD, S	N/A	Biopsy	N/A	1	0	0	1	CR	0	3.0	3.0	1
**Capone et al. 1990**[44]	Case Series – IV	5 / 2 (40%)	63 (37-71)	1 Breast, Gastric, Kidney, Melanoma, Skin (20%)	24 (6-48)	2 B (40%)<br>2 R (40%)<br>1 L (20%)	3 Diffuse (60%)<br>2 Lateral (40%)	5 Muscles (100%)	0	4 DI (80%),<br>3 IM (60%),<br>3 Pro (60%),<br>2 OP (40%),<br>2 RE (40%),<br>1 DV (20%)	N/A	4 FNAB (80%)<br>1 Biopsy (20%)	N/A	5 (100%)	4 (80%), 30 Gy	0	1 (20%)	1 PR (20%)	0	4.0 (3-23)	4.0 (3-23)	1 (20%)
**Friedman et al. 1990**[45]	Case Report – V	1 / 0	72	Thyroid	6.0	R	Medial	Muscle	0	OP, RE, RAPD	N/A	Partial	N/A	0	0	0	1	N/A	0	12.0	12.0	0
**Goldberg et al. 1990**[46]	Case Series – IV	36 / 24 (66.7%)	64 (35-86)	13 Breast (36.1%),<br>5Melanoma (13.9%)<br>4 Prostate (11.1%),<br>3 CUP (8.3%),<br>3Smallbowel (8.3%)<br>2 Lung (5.6%)<br>1 Carcinoid (2.8%),<br>1 Ovary (2.8%),<br>1 Pancreas (2.8%),<br>1 Skin (2.8%),<br>1 Soft Tissue (2.8%)<br>1 Uterus (2.8%)	29.5 (0-240)	17 L (47.2%)<br>16 R (44.4%)<br>3 B (8.3%)	6 Medial (16.7%)<br>4 Lateral (11.1%)<br>4 Superior (11.1%)<br>3 Diffuse (8.3%)<br>3 Inferior (8.3%)	11 Soft Tissue (30.6%)<br>10 Bone (27.8%)<br>4 Muscle (11.1%)	0	21 IM (58.3%)<br>17 D (47.2%)<br>13 Pro (36.1%)<br>13 Pt (36.1%)<br>12 Mass (33.3%)<br>10 OP (27.8%)<br>9 En (25%)<br>8 DV (22.2%)<br>8 S (22.2%)<br>1 RAPD (2.8%)	N/A	N/A	N/A	N/A	N/A	N/A	N/A	N/A	N/A	4.0 (1-76)	40. (1-76)	0
**Shetlar et al. 1990**[47]	Case Series – IV	3 / 2 (66.7%)	51 (38-71)	3 Carcinoid (100%)	46.0 (9-69)	2 L (66.7%)<br>1 R (33.3%)	2 Posterior (66.7%)<br>1 Medial (33.3%)	2 Soft Tissue (66.7%)<br>1 Muscle (33.3%)	0	3 Pro (100%)<br>1 RAPD (33.3%)	N/A	N/A	N/A	0	0	0	0	N/A	0	12.0 (5-24)	12.0 (5-24)	2 (66.7%)
**Wakisaka et al. 1990**[48]	Case Report – V	1 / 0	58	Liver	0	L	Superolateral	Soft Tissue	1	D, Pro, Pt	Osteolytic	Complete	N/A	0	0	0	1	N/A	0	10.0	10.0	0
**Felip et al. 1991**[49]	Case Report – V	1 / 1	64	Breast	0	R	Medial	Muscle	0	D, Pro, VL	N/A	Biopsy	N/A	0	0	0	0	N/A	0	3.0	3.0	1
**Glazer et al. 1991**[50]	Case Report – V	1 / 1	64	Breast	0	R	Medial	Muscle	0	RE, S	N/A	Biopsy	N/A	1	1, 35 Gy	0	1	N/A	0	12.0	12.0	1
**Van der Heijden et al. 1991**[51]	Case Report – V	1 / 1	47	Breast	60.0	R	Inferior	Muscle	0	D, IM, Mass	N/A	Biopsy	N/A	0	1	0	1	PR	0	12.0	12.0	1
**Zucker et al. 1991**[52]	Case Report – V	1 / 1	83	Skin	60.0	R	Inferior	Soft Tissue	0	D, IM, Pro	N/A	Biopsy	N/A	0	1	0	1	PR	0	3.0	3.0	1
**Brackup et al. 1992**[53]	Case Report – V	1 / 0	54	Melanoma	48.0	R	Lateral	Muscle	0	IM, OP, Pro	N/A	Partial	Decompression	0	1, 50 Gy	0	0	PR	0	9.0	9.0	0
**Di Leo et al. 1992**[54]	Case Report – V	1 / 1	77	Prostate	0	L	Medial	Muscle	0	D, OP, Pro	N/A	Biopsy	N/A	0	0	Hormonal	1	PR	0	14.0	14.0	1

**Shetty et al. 1992**[55]	Case Report – V	1 / 1	74	Colorectal	24.0	L	Superior	Bone	0	Mass, Pro	N/A	Biopsy	N/A	0	1	0	0	PD	0	2.0	2.0	0
**Tijl et al. 1992**[56]	Case Series – IV	34 / 27 (79.4%)	65 (32-88)	20 Breast (58.8%)<br>5 Prostate (14.7%)<br>3 Melanoma (8.8%)<br>2 Kidney (5.9%)<br>2 Soft Tissue (5.9%)<br>1 Lung (2.9%)<br>1 Thyroid (2.9%)	21.0 (0-240)	18 R (52.9%)<br>12 L (35.3%)<br>4 B (11.8%)	N/A	N/A	N/A	15 Pro (44.1%)<br>13 D (38.2%)<br>7 Pt (20.6%)<br>6 En (17.6%)<br>5 IM (14.7%)<br>5 Mass (14.7%)<br>3 DV (8.8%)<br>2 OP (5.9%)	N/A	34 Surgery (100%)<br>7 Partial (20.6%)	N/A	7 (20.6%)	20 (58.8%)	13 Hormonal (38.2%)	N/A	24 PR (70.6%)<br>4 PD (11.8%)	0	26.0 (4-75)	26.0 (4-75)	16 (47%)
**Vanderpum et al. 1992**[57]	Case Report – V	1 / 0	61	Thyroid	0	R	Medial	Muscle	0	D, DV, OP, Pro	N/A	Partial	N/A	0	0	0	1	PR	0	12.0	12.0	0
**Bersani et al. 1994**[58]	Case Report – V	1 / 0	50	Kidney	180.0	L	Superolateral	Soft Tissue	0	DV, OP, Pro, RAPD	N/A	Complete	N/A	0	1	0	1	PR	0	12.0	12.0	1
**Ellis et al. 1994**[59]	Case Report – V	1 / 0	86	Melanoma	0	L	Superior	Soft Tissue	0	IM, Mass, Pro, Pt	N/A	Biopsy	N/A	0	1, 31 Gy	0	0	SD	0	18.0	18.0	0
**Hugkulstone et al. 1994**[60]	Case Report – V	1 / 0	45	Bladder	0	B	Medial	Soft Tissue	0	D, IM, Mass	N/A	Biopsy	N/A	1	1	0	0	PD	0	5.0	5.0	0
**Loo et al. 1994**[61]	Case Report – V	1 / 1	71	Liver	0	R	Superior	Muscle	1	DV, IM	Osteolytic	Complete	N/A	0	0	0	0	PD	0	3.0	3.0	0
**Tranfa et al. 1994**[62]	Case Report – V	1 / 0	85	Liver	0	R	Superolateral	Soft Tissue	0	DV, OP, Pro	Osteolytic	Partial	N/A	0	0	0	1	N/A	0	3.0	3.0	1
**Aburn et al. 1995**[63]	Case Report – V	1 / 0	64	Carcinoid	11.0	L	Superior	Bone	0	D, DV, Pro	N/A	Partial	N/A	0	1, 30 Gy	0	1	PR	0	12.0	12.0	0
**Airoldi et al. 1995**[64]	Case Report – V	1 / 0	77	Kidney	77.0	R	Medial	Bone	0	Pro	Osteolytic	Complete	Exenteration	0	1	0	1	N/A	0	14.0	14.0	0
**Day et al. 1995**[65]	Case Report – V	1 / 0	55	Salivary Gland	0	L	Posterior	Soft Tissue	0	D, S	N/A	Complete	Exenteration	0	1, 60 Gy	0	1	CR	0	72.0	72.0	1
**Fan et al. 1995**[66]	Case Series – IV	3 / 2 (66.7%)	58 (51-85)	3 Carcinoid (100%)	0	2 L (66.7%)<br>1 R (33.3%)	N/A	N/A	N/A	2 IM (66.7%)<br>2 Pro (66.7%)<br>1 D (33.3%)<br>1 DV (33.3%)	N/A	3 Biopsy (100%)	N/A	2 (66.7%)	2 (66.7%)	0	1 (33.3%)	2 PR (66.7%)	0	12.0 (0.5-24)	12.0 (0.5-24)	0
**Rhatigan et al. 1995**[67]	Case Report – V	1 / 1	64	Breast	60.0	B	Superolateral	Soft Tissue	0	IM, OP, Pro	N/A	Biopsy	N/A	0	1	0	0	PD	0	0.5	0.5	0
**Thomas et al. 1995**[68]	Case Report – V	1 / 1	58	Salivary Gland	192.0	R	Lateral	Muscle	0	Pro, S	N/A	Partial	Decompressi on	1	0	0	1	CR	0	12.0	12.0	1
**Burnstine et al. 1996**[69]	Case Report – V	1 / 1	46	Breast	24.0	R	Inferior	Muscle	0	D, OP, Pro	N/A	Partial	N/A	0	1, 60 Gy	0	0	N/A	1	4.0	11.0	0
**El-Toukhy et al. 1996**[70]	Case Report – V	1 / 0	45	Carcinoid	0	R	Superior	Soft Tissue	1	Pro, Pt, RAPD	N/A	Partial	Decompressi on	0	0	0	1	N/A	1	36.0	144.0	1
**Char et al. 1997**[71]	Case Series – IV	31 / 16 (51.6%)	57 (37-77)	9 Breast (29%)<br>8 Melanoma (25.8%)<br>6 CUP (19.3%)<br>2 Prostate (6.4%)<br>1 Carcinoid, Lung, Kidney, Salivary, SoftTissue, Stomach (3.2%)	N/A	N/A	N/A	N/A	N/A	15 D (48.4%)<br>8 Pro (25.8%)<br>5 DV (16.1%)<br>1 RE (3.2%)	N/A	16 Partial (51.6%)<br>15 FNAB (43.4%)	N/A	N/A	N/A	N/A	N/A	N/A	0	15.0	15.0	N/A
**Fezza et al. 1997**[72]	Case Report – V	1 / 1	75	Soft Tissue	180.0	L	Medial	Muscle	0	DV, IM, Pro	N/A	Biopsy	N/A	0	1, 46 Gy	0	1	N/A	0	2.0	2.0	1
**Hayashi et al. 1997**[73]	Case Report – V	1 / 1	63	Bone	324.0	R	Posterior	Bone	1	DV	Osteolytic	Complete	N/A	0	1, 50 Gy	0	0	N/A	0	6.0	6.0	1
**Logrono et al. 1997**[74]	Case Report – V	1 / 0	45	Soft Tissue	21.0	L	Superior	Fat	0	Pro, S	0	FNAB	N/A	0	1, 50 Gy	0	1	N/A	0	8.0	8.0	0
**Mezer et al. 1997**[75]	Case Report – V	1 / 1	70	Kidney	84.0	R	Inferolateral	Soft Tissue	0	D, DV, Mass	N/A	Partial	N/A	0	1, 50 Gy	0	1	N/A	1	2.0	4.0	1

**Sekundo et al. 1997**[76]	Case Report – V	1 / 1	54	Breast	0	L	Superomedial	Fat	0	IM, Mass, Pro	N/A	Biopsy	N/A	1	0	0	1	N/A	0	24.0	24.0	1
**Zambarakji et al. 1997**[77]	Case Report – V	1 / 1	49	Breast	6.0	R	Diffuse	Muscle	0	D, IM	N/A	Biopsy	N/A	0	1	0	1	N/A	0	3.0	3.0	1
**Font et al. 1998**[78]	Case Report – V	1 / 1	79	Liver	0	R	Lateral	Bone	0	IM, OP, Pro, RAPD, S	Osteolytic	Biopsy	N/A	0	1	0	1	PR	0	36.0	36.0	1
**Garcia et al. 1998**[79]	Case Report – V	1 / 0	43	Breast	0	B	Diffuse	Soft Tissue	0	IM, Pro	N/A	Biopsy	N/A	0	1, 45 Gy	0	1	PR	0	3.0	3.0	1
**Schaefer-Hirschkind et al. 1998**[80]	Case Report – V	1 / 0	81	Kidney	36.0	L	Posterior	Soft Tissue	0	OP, Pro	N/A	Biopsy	N/A	1	1	0	0	PD	0	3.0	3.0	1
**Toller et al. 1998**[81]	Case Report – V	1 / 1	47	Breast	96.0	B	Diffuse	Soft Tissue	0	D, DV, OP, RAPD	N/A	Biopsy	N/A	0	0	0	0	PD	0	9.0	9.0	0
**Collins et al. 1999**[82]	Case Report – V	1 / 0	47	Esophagus	12.0	L	Superomedial	Soft Tissue	0	Mass	N/A	Biopsy	N/A	N/A	N/A	N/A	N/A	N/A	0	3.0	3.0	1
**Scolyer et al. 1999**[83]	Case Report – V	1 / 0	77	Liver	2.0	R	Superolateral	Bone	0	S	N/A	Complete	N/A	N/A	N/A	N/A	N/A	N/A	0	3.0	3.0	1
**Wein et al. 1999**[84]	Case Report – V	1 / 1	62	Carcinoid	0	L	Posterior	Soft Tissue	0	DV, RAPD	N/A	Biopsy	N/A	1	1	0	0	PD	0	3.0	3.0	1
**Wolstencroft et al. 1999**[85]	Case Report – V	1 / 1	53	Breast	0	L	Diffuse	Soft Tissue	0	DV, En	N/A	Biopsy	N/A	1	1	Hormonal	N/A	SD	0	4.0	4.0	1
**Callejo et al. 2000**[86]	Case Report – V	1 / 0	72	Soft Tissue	0	L	Lateral	Fat	0	D, IM, Mass, Pro, RAPD	N/A	Partial	N/A	0	1, 30 Gy	0	1	PR	0	9.0	9.0	0
**Daumerie et al. 2000**[87]	Case Report – V	1 / 1	59	Thyroid	0	L	Superolateral	Bone	1	DV, Pro, S	Osteolytic	Biopsy	N/A	0	0	Radioiodine	1	PR	0	36.0	36.0	0
**Friedrich et al. 2000**[88]	Case Report – V	1 / 1	61	Salivary Gland	180.0	R	Lateral	Bone	0	IM, Pro	Osteolytic	Partial	N/A	0	1	0	1	PR	0	36.0	36.0	0
**Stuntz et al. 2000**[89]	Case Report – V	1 / 0	40	Breast	0	B	Diffuse	Soft Tissue	0	DV, Mass, OP	N/A	Biopsy	N/A	1	1	0	0	PD	0	34.0	34.0	0
**Bartley et al. 2001**[90]	Case Report – V	1 / 0	24	Adrenal Gland	24.0	R	Superolateral	Bone	1	D, OP, Pt	Osteolytic	Partial	Decompression	0	1, 37.5 Gy	0	N/A	N/A	1	8.0	15.0	0
**Shields et al. 2001**[91]	Case Series – IV	70 / 45 (64.3%)	N/A	40 Breast (57.1%)<br>10 Prostate (14.3%)<br>7 Lung (10%)<br>4 Kidney (5.7%)<br>4 Melanoma (5.7%)<br>3 Carcinoid (4.3%)<br>2Smallbowel (2.8%)	55.0 (6-90)	34 R (48.6%)<br>33 L (47.1%)<br>3 B (4.3%)	41 Diffuse (58.6%)	41 Soft Tissue (58.6%),<br>23 Bone (32.9%)	N/A	38 IM (54.3%)<br>35 Pro (50%)<br>31 Mass (44.3%)<br>16 DV (22.9%)<br>12 OP (17.1%)<br>8 En (11.4%)<br>6 D (8.6%)	23 Osteolytic (32.9%)	52 Biopsy (74.3%)<br>18 Complete (25.7%)	N/A	50 (71.4%)	39 (55.7%), 35 Gy	0	N/A	N/A	0	20.0	20.0	N/A
**Maheshwari et al. 2002**[92]	Case Report – V	1 / 0	56	Tongue	0	L	Diffuse	Fat	0	DV, OP, IM, Pro, S	N/A	Biopsy	N/A	1	1, 45 Gy	0	1	CR	0	13.0	13.0	1
**McCulley et al. 2002**[93]	Case Report – V	1 / 1	29	Cervix	1.0	R	Inferior	Soft Tissue	0	IM, Pro	N/A	Biopsy	N/A	1	1	0	0	PD	0	8.0	8.0	0
**Misra et al. 2002**[94]	Case Report – V	1 / 1	40	Gallbladder	2.0	L	Superomedial	Muscle	0	D, IM	N/A	FNAB	N/A	0	0	0	0	PD	0	2.0	2.0	1
**Baltogiannis et al. 2003**[95]	Case Report – V	1 / 0	79	Prostate	1.0	L	Medial	Bone	0	DV, IM, Pro	N/A	Complete	Exenteration	N/A	N/A	N/A	N/A	N/A	0	10.0	10.0	0
**Baroody et al. 2003**[96]	Case Series – IV	2 / 1	37 (37-38)	2 Melanoma (100%)	19.5 (3-36)	R	1 Posterior (50%)<br>1 Superomedial (50%)	2 Soft Tissue (100%)	0	IM	N/A	1 Biopsy (50%)<br>1 Complete (50%)	1Exenteration (50%)	0	0	2Immunotherapy (100%)	1	N/A	0	36.0 (26-46)	36.0 (26-46)	1 (50%)
**Fynn-Thompson et al. 2003**[97]	Case Report – V	1 / 0	68	Bladder	48.0	L	Diffuse	Soft Tissue	0	D, DV, Pro	N/A	Biopsy	N/A	0	0	0	0	PD	0	1.0	1.0	0
**Holland et al. 2003**[98]	Case Series – IV	20 / 11 (55%)	67 (29-90)	8 Breast (40%)<br>3Smallbowel (15%)<br>2 CUP (10%)<br>2 Lung (10%)<br>2 Melanoma (10%)<br>2 Prostate (10%)	N/A	10 L (50%)<br>7 R (35%)<br>3 B (15%)	N/A	9 Soft Tissue (45%)<br>7 Muscle (35%)<br>4 Bone (20%)	0	11 S (55%)<br>10 D (50%)<br>9 RE (45%)<br>7 Pro (35%)<br>4 OP (20%)<br>3 DV (15%)	N/A	11 Partial (55%)<br>9 Biopsy (45%)	2Decompression (10%)	4 (20%)	7 (35%)	1 Hormonal (5%)	N/A	N/A	0	14.7 (0.5-107)	14.7 (0.5-107)	1 (5%)

				1 Bladder (5%)						1 En (5%)												
**Lekse et al. 2003**[99]	Case Report – V	1 / 1	78	Small bowel	4.0	R	Inferior	Muscle	0	D	N/A	Biopsy	N/A	0	0	0	0	PD	0	3.0	3.0	0
**Saleh et al. 2003**[100]	Case Report – V	1 / 1	54	Salivary Gland	5.0	L	Medial	Fat	0	D	N/A	Partial	N/A	0	1	0	0	PR	0	6.0	6.0	1
**Takemoto et al. 2003**[101]	Case Report – V	1 / 0	39	Carcinoid	120.0	R	Superior	Bone	1	D	N/A	Biopsy	N/A	N/A	N/A	N/A	N/A	N/A	0	2.0	2.0	1
**Tehrani et al. 2003**[102]	Case Report – V	1 / 0	72	Soft Tissue	24.0	L	Medial	Soft Tissue	0	D, Mass, Pro	0	Biopsy	N/A	1	1, 30 Gy	0	0	PD	0	7.0	7.0	0
**Zografos et al. 2003**[103]	Case Series – IV	7 / 4 (57.1%)	54 (34-77)	7 Melanoma (100%)	65.0 (7-106)	N/A	N/A	N/A	N/A	5 IM (71.4%)<br>5 Pro (71.4%)<br>4 Mass (57.1%)<br>4 RE (57.1%)<br>2 DV (28.6%)<br>2 Pt (28.6%)<br>1 OP (14.3%)<br>1 S (14.3%)	N/A	3 Partial (42.8%)<br>1 Complete (14.3%)	1Exenteration (14.3%)	3 (42.8%)	1 (14.3%), 40 Gy	0	N/A	2 CR (28.6%),<br>2 PR (28.6%),<br>2 PD (28.6%),<br>1 SD (14.3%)	0	5.0 (1-48)	5.0 (1-48)	2 (28.6%)
**Chua et al. 2004**[104]	Case Report – V	1 / 1	78	Bladder	48.0	R	Superolateral	Soft Tissue	1	IM, Mass, Pro, Pt	N/A	Biopsy	N/A	1	1	0	0	PD	0	1.0	1.0	0
**Glazer-Hockstein et al. 2004**[105]	Case Report – V	1 / 0	61	Soft Tissue	48.0	R	Lateral	Bone	1	Pro, S	N/A	Complete	N/A	N/A	N/A	N/A	N/A	N/A	0	2.0	2.0	1
**Lell et al. 2004**[106]	Case Report – V	1 / 1	63	Breast	0	B	Diffuse	Soft Tissue	0	Pro, S	N/A	Biopsy	N/A	N/A	N/A	N/A	N/A	N/A	0	3.0	3.0	1
**Van der Zee et al. 2004**[107]	Case Report – V	1 / 1	77	Breast	12.0	L	Superomedial	Bone	0	D	N/A	Complete	N/A	0	1, 30 Gy	Hormonal, LITT	1	PD	1	10.0	35.0	1
**Zdinak et al. 2004**[108]	Case Report – V	1 / 0	39	Kidney	0	L	Medial	Muscle	0	IM, OP, Pro	N/A	Partial	Decompression	0	0	Immunotherapy	0	N/A	0	3.0	3.0	0
**Borota et al. 2005**[109]	Case Report – V	1 / 0	61	Carcinoid	15.0	L	Inferior	Muscle	0	DV, IM, Pro	N/A	Complete	N/A	1	0	Immunotherapy	1	CR	0	3.0	3.0	1
**Challagundla et al. 2005**[110]	Case Report – V	1 / 0	53	Prostate	N/A	R	Diffuse	Muscle	1	D, Pro, RAPD	N/A	Biopsy	N/A	1	1, 40 Gy	0	1	PR	0	6.0	6.0	0
**Fabi et al. 2005**[111]	Case Report – V	1 / 0	53	Soft Tissue	24.0	B	Diffuse	Muscle	0	D, Pro	N/A	Biopsy	N/A	1	1, 30 Gy	0	1	PD	0	7.0	7.0	1
**Gupta et al. 2005**[112]	Case Report – V	1 / 0	45	Liver	0	L	Superolateral	Bone	1	IM, Mass, Pro	N/A	Biopsy	N/A	0	0	0	0	PD	0	1.0	1.0	1
**Hart et al. 2005**[113]	Case Report – V	1 / 0	70	Kidney	18.0	L	Lateral	Muscle	0	D, IM, Pro, S	N/A	Biopsy	N/A	N/A	N/A	N/A	N/A	N/A	0	1.0	1.0	1
**Konuk et al. 2005**[114]	Case Report – V	1 / 0	44	Soft Tissue	36.0	L	Superolateral	Soft Tissue	1	DV, Pro, RAPD	N/A	Partial	Decompression	0	1, 30 Gy	0	0	PR	0	6.0	6.0	1
**Mohadjer et al. 2005**[115]	Case Report – V	1 / 1	50	Breast	0	R	Superolateral	Fat	N/A	OP, Pro	N/A	Biopsy	N/A	1	1, 30 Gy	0	1	PR	0	120.0	120.0	1
**Puglisi et al. 2005**[116]	Case Report – V	1 / 0	53	Gallbladder	1.0	R	Lateral	Muscle	0	Pro	N/A	FNAB	N/A	1	1	0	0	PD	0	6.0	6.0	1
**Aralikatti et al. 2006**[117]	Case Report – V	1 / 0	63	Esophagus	5.0	L	Superolateral	Soft Tissue	1	Mass, S	N/A	Biopsy	N/A	0	1	0	0	PD	0	2.0	2.0	0
**Lee et al. 2006**[118]	Case Report – V	1 / 0	56	Stomach	0	R	Posterior	Soft Tissue	0	D	N/A	Complete	N/A	1	0	0	1	SD	0	12.0	12.0	1
**Mehta et al. 2006**[119]	Case Series – IV	13 / 4 (30.8%)	68 (40-75)	13 Carcinoid (100%)	N/A	N/A	N/A	7 Muscle (53.8%)<br>6 Fat (46.1%)	N/A	9 Pro (69.2%)<br>4 DV (30.8%)<br>4 IM (30.8%)<br>2 D (15.4%)<br>1 Mass (7.7%)<br>1 No sympt (7.7%)	N/A	13 Surgery (100%)<br>4 Complete (30.8%)	4Exenteration (30.8%)	1 (7.7%)	9 (69.2%)	N/A	N/A	N/A	N/A	N/A	N/A	N/A
**Oida et al. 2006**[120]	Case Report – V	1 / 0	72	Liver	120.0	L	Superolateral	Soft Tissue	1	D, DV	Osteolytic	Partial	Decompression	0	0	0	1	PR	0	4.0	4.0	0
**Schick et al. 2006**[121]	Case Series – IV	10 / 4 (40%)	N/A	5 Lung (50%)<br>3 Breast (30%)	3.0 (0-132)	N/A	7 Posterior (70%)<br>3 Diffuse (20%)	6 Soft Tissue (60%)	0	N/A	N/A	3 Complete (30%)	N/A	4 (40%)	4 (40%)	1 Hormonal	N/A	N/A	3 (30%)	11.0 (1-36)	11.5 (1-36)	1 (10%)

				1 CUP (10%)<br>1 Uterus (10%)				4 Bone (40%)				3 Partial (30%)										
**Sharma et al. 2006**[122]	Case Report – V	1 / 0	69	Kidney	0	L	Superolateral	Soft Tissue	1	Mass, Pro, Pt	Osteolytic	Biopsy	N/A	0	1	0	1	PR	0	4.0	4.0	1
**Singh et al. 2006**[123]	Case Report – V	1 / 1	45	Ovary	36.0	L	Posterior	Fat	0	OP, S	N/A	Partial	N/A	0	0	0	0	PD	0	2.0	2.0	0
**Solari et al. 2006**[124]	Case Series – IV	1 / 1	81	Breast	24.0	L	Superolateral	Soft Tissue	0	Mass, Pro, RE	N/A	Biopsy	N/A	0	1	0	1	PR	0	3.0	3.0	1
**Tumuluri et al. 2006**[125]	Case Report – V	1 / 1	75	Esophagus	6.0	L	Medial	Soft Tissue	0	D, OP, Pt	N/A	Biopsy	N/A	0	1	0	1	PR	0	12.0	12.0	1
**Yunker et al. 2006**[126]	Case Report – V	1 / 1	69	Stomach	0	L	Superomedial	Soft Tissue	0	D, IM, Mass, Pt	N/A	Biopsy	N/A	N/A	N/A	N/A	N/A	N/A	0	2.0	2.0	1
**Char et al. 2007**[127]	Case Report – V	1 / 1	87	Uterus	8.0	R	Superior	Fat	0	OP, Pt, S	N/A	Complete	N/A	N/A	N/A	N/A	N/A	N/A	0	2.0	2.0	1
**Gerencer et al. 2007**[128]	Case Report – V	1 / 1	58	Carcinoid	12.0	R	Medial	Muscle	0	D, IM, VL	N/A	Biopsy	N/A	0	1, 44 Gy	0	1	PR	0	24.0	24.0	1
**Isshiki et al. 2007**[129]	Case Report – V	1 / 0	70	Prostate	0	L	Superolateral	Bone	1	DV, IM, Pro	Osteolytic	Complete	N/A	0	0	0	1	CR	0	12.0	12.0	1
**Lin et al. 2007**[130]	Case Report – V	1 / 0	60	Bladder	8.0	R	Superomedial	Soft Tissue	0	D, DV, Pro	N/A	Biopsy	N/A	1	0	0	0	PR	0	6.0	6.0	1
**Mani et al. 2007**[131]	Case Report – V	1 / 1	65	Larynx	5.0	R	Superomedial	Bone	0	D, DV	Osteolytic	Biopsy	N/A	0	0	0	0	PD	0	1.0	1.0	0
**Su et al. 2007**[132]	Case Report – V	1 / 1	55	Uterus	18.0	R	Superolateral	Soft Tissue	1	DV, Pro, RAPD	Osteolytic	Complete	Exenteration	0	1, 50 Gy	0	1	PR	0	12.0	12.0	1
**Talwar et al. 2007**[133]	Case Report – V	1 / 1	30	Breast	15.0	R	Lateral	Muscle	0	Pro, S	N/A	Partial	N/A	0	1	Hormonal	1	PR	0	3.0	3.0	1
**Torres et al. 2007**[134]	Case Series – IV	2 / 0	65.5 (53-78)	2 Breast (100%)	26.0 (4-48)	1 L (50%)<br>1 R (50%)	1 Lateral (50%)<br>1 Superior (50%)	1 Bone (50%)<br>1 Soft Tissue (50%)	0	2 IM (100%)<br>1 D (50%)<br>1 Mass (50%)<br>1 Pro (50%)	N/A	2 Partial	N/A	1 (50%)	0	0	2	N/A	0	17.0 (2-32)	17.0 (2-32)	2 (100%)
**Uluocak et al. 2007**[135]	Case Report – V	1 / 0	73	Prostate	0	L	Superolateral	Soft Tissue	1	OP, Pro, VL	Osteolytic	FNAB	N/A	0	0	Hormonal	1	PR	0	12.0	12.0	0
**Alsuhaibani et al. 2008**[136]	Case Report – V	1 / 0	90	Prostate	12.0	L	Medial	Muscle	0	D, OP	N/A	Biopsy	N/A	0	1	0	1	PR	0	8.0	8.0	0
**Hatton et al. 2008**[137]	Case Report – V	1 / 1	24	Soft Tissue	24.0	B	Doffise	Muscle	0	IM, Pro, RAPD, S	N/A	Biopsy	N/A	0	1	0	0	PD	0	2.0	2.0	0
**Henning et al. 2008**[138]	Case Report – V	1 / 0	60	Lung	0	R	Superomedial	Bone	0	D, Pro, S	N/A	Biopsy	N/A	1	0	0	0	PD	0	6.0	6.0	0
**Hirunwiwatkul et al. 2008**[139]	Case Report – V	1 / 1	74	Liver	0	R	Lateral	Bone	1	DV, IM, OP, Pro, Pt, RAPD	Osteolytic	Biopsy	N/A	1	1	0	0	PD	0	2.0	2.0	0
**Kiratli et al. 2008**[140]	Case Report – V	1 / 0	74	Carcinoid	36.0	R	Inferior	Muscle	0	D, Pro	N/A	Complete	N/A	1	0	0	1	PR	0	24.0	24.0	1
**Kuo et al. 2008**[141]	Case Report – V	1 / 1	66	Breast	£1.0	R	Superior	Fat	0	S	N/A	Biopsy	N/A	1	0	0	1	CR	0	3.0	3.0	1
**Milman et al. 2008**[142]	Case Report – V	1 / 1	83	Breast	336.0	L	Diffuse	Muscle	0	IM, Mass	N/A	Partial	N/A	1	0	Hormonal	1	PR	0	15.0	15.0	1
**Mudiyanselage et al. 2008**[143]	Case Report – V	1 / 1	60	Kidney	0	L	Medial	Muscle	0	D, Pro	N/A	Complete	N/A	1	0	0	1	N/A	0	3.0	3.0	1
**Peng et al. 2008**[144]	Case Report – V	1 / 0	72	Prostate	0	L	Inferomedial	Soft Tissue	0	Mass, Pro	N/A	Complete	Exenteration	N/A	N/A	N/A	N/A	N/A	0	60.0	60.0	1
**Pitts et al. 2008**[145]	Case Series – IV	2 / 1	54 (47-61)	2 Liver (100%)	0	2 L (100%)	2 Lateral (100%)	2 Soft Tissue (100%)	2 (100%)	1 OP, Pro (50%)<br>1 DV, S (50%)<br>1 RAPD (50%)	2 Osteolytic (100%)	2 FNAB (100%)	N/A	0	1 (50%), 10 Gy	0	0	2 PD (100%)	0	1 (0.5-2)	1 (0.5-2)	0
**Polito et al. 2008**[146]	Case Report – V	1 / 0	65	Breast	60.0	L	Inferior	Fat	0	En	0	Biopsy	N/A	0	1, 30 Gy	0	1	PR	0	15.0	15.0	0
**Preechawal et al. 2008**[147]	Case Report – V	1 / 0	48	Kidney	0	L	Superior	Muscle	0	IM, Mass, Pro, Pt	N/A	Biopsy	N/A	0	0	0	0	PD	0	2.0	2.0	0

**Rocha Filho et al. 2008**[148]	Case Report – V	1 / 1	66	Thyroid	0	R	Superolateral	Bone	0	Pro	N/A	Biopsy	N/A	1	1, 37.5 Gy	0	0	SD	0	3.0	3.0	1
**Seiff et al. 2008**[149]	Case Report – V	1 / 1	46	Thyroid	256.0	L	Inferior	Muscle	0	Mass, Pro	N/A	Partial	N/A	0	0	0	1	PR	0	2.0	2.0	1
**Souayah et al. 2008**[150]	Case Report – V	1 / 0	49	Stomach	0	R	Lateral	Muscle	0	D, IM, S	N/A	Biopsy	N/A	0	1	0	0	PD	0	2.5	2.5	0
**Surace et al. 2008**[151]	Case Report – V	1 / 1	73	Breast	420.0	L	Diffuse	Muscle	0	D, En, IM, Pt	N/A	Biopsy	N/A	0	0	Hormonal	1	PR	0	3.0	3.0	1
**Gosslee et al. 2009**[152]	Case Report – V	1 / 1	36	Cervix	30.0	L	Superolateral	Bone	0	IM, Mass, OP, S	N/A	Partial	N/A	0	1	0	1	PR	0	3.0	3.0	1
**Nirmala et al. 2009**[153]	Case Report – V	1 / 0	62	Thymus	19.0	L	Medial	Soft Tissue	0	DV, Pro	N/A	FNAB	N/A	0	1, 40 Gy	0	1	PR	0	3.0	3.0	1
**Slagle et al. 2009**[154]	Case Report – V	1 / 0	61	CUP	0	R	Superior	Muscle	0	D, OP	Osteolytic	FNAB	N/A	0	1, 33 Gy	0	0	PD	0	6.0	6.0	1
**Valenzuela et al. 2009**[155]	Case Series – IV	80 / 36 (45%)	60	23 Breast (28.7%)<br>16 Melanoma (20%)<br>10 Prostate (12.5%)<br>7 Carcinoid (8.8%)<br>6 Skin (7.5%)<br>5 Lung (6.3%)<br>3 Colorectal (3.8%)<br>3 Salivary (3.8%)<br>2 Bone (2.5%)<br>2 Ovary (2.5%)<br>2 Uterus (2.5%)<br>1 Liver (1.2%)	N/A	39 L (48.8%)<br>39 R (48.8%)<br>2 B (2.5%)	18 Medial (22.5%)<br>14 Lateral (17.5%)<br>11 Posterior (13.8%)<br>11 Superior (13.8%)	34 Fat (42.5%)<br>22 Muscle (27.5%)<br>22 Soft Tissue (27.5%)	0	39 D (48.8%)<br>33 OP (41.2%)<br>24 VL (30%)<br>23 Pro (28.8%)<br>20 S (25%)<br>13 Pt (16.3%)<br>9 Mass (11.3%)	6 Osteolytic (7.5%)	58 Biopsy (72.5%)<br>14 FNAB (17.5%)<br>8 Partial (10%)	N/A	19 (23.8%)	54 (67.5%)	18 Hormonal (22.5%)	N/A	N/A	0	18.0	18.0	N/A
**Barahimi et al. 2010**[156]	Case Report – V	1 / 0	68	Prostate	72.0	R	Lateral	Soft Tissue	0	IM. OP, Pt, RE, S	N/A	Biopsy	N/A	0	0	0	0	PD	0	8.0	8.0	0
**Foo et al. 2010**[157]	Case Report – V	1 / 0	71	Pancreas	0	L	Lateral	Soft Tissue	0	D, DV, OP, RAPD	N/A	Partial	Decompression	0	1	0	0	SD	0	1.0	1.0	0
**Francone et al. 2010**[158]	Case Report – V	1 / 1	66	Breast	0	R	Inferior	Soft Tissue	0	S	N/A	Biopsy	N/A	1	1	Hormonal	1	PR	0	12.0	12.0	1
**Hassane et al. 2010**[159]	Case Report – V	1 / 0	61	Esophagus	0	L	Superolateral	Bone	1	OP, Mass	Osteolytic	Biopsy	N/A	0	0	0	0	PD	0	1.0	1.0	`
**Matsuo et al. 2010**[160]	Case Report – V	1 / 1	72	Carcinoid	9.0	R	Medial	Muscle	0	DV, S	N/A	Partial	N/A	0	1, 44 Gy	0	0	SD	0	3.0	3.0	1
**Ng et al. 2010**[161]	Case Report – V	1 / 0	55	Esophagus	12.0	B	Superior	Soft Tissue	0	Pro, Pt	N/A	Biopsy	N/A	1	1, 30 Gy	0	1	PR	0	3.0	3.0	0
**Patel et al. 2010**[162]	Case Report – V	1 / 0	69	Prostate	0	R	Superior	Bone	1	Pro, Pt	Osteoblastic	Biopsy	Decompression	0	0	Hormonal	1	PR	0	6.0	6.0	1
**Eckardt et al. 2011**[163]	Case Report – V	1 / 1	63	Breast	36.0	L	Superolateral	Bone	1	D, OP, Pro	Osteolytic	Biopsy	N/A	1	0	Hormonal	1	N/A	0	3.0	3.0	1
**Galm et al. 2011**[164]	Case Report – V	1 / 0	75	Larynx	10.0	L	Superior	Soft Tissue	0	OP, RAPD, RE	N/A	Biopsy	N/A	0	0	0	0	PD	0	1.0	1.0	0
**Guerriero et al. 2011**[165]	Case Report – V	1 / 0	45	Liver	288.0	L	Superolateral	Soft Tissue	0	D, Mass, OP, Pro	Osteolytic	Biopsy	N/A	0	1	0	1	PR	0	3.0	3.0	1
**Gupta et al. 2011**[166]	Case Report – V	1 / 1	46	Breast	0	L	Inferomedial	Muscle	0	OP, S	N/A	Complete	N/A	0	0	0	1	CR	0	6.0	6.0	1
**Janicijevic-Petrovic et al. 2011**[167]	Case Report – V	1 / 1	45	Breast	0	R	Posterior	Soft Tissue	0	Pro	N/A	Biopsy	N/A	N/A	N/A	N/A	N/A	N/A	0	3.0	3.0	1
**Konoglou et al. 2011**[168]	Case Report – V	1 / 0	69	Lung	0	R	Inferolateral	Fat	0	Pro	N/A	FNAB	N/A	1	0	0	1	CR	0	4.0	4.0	0
**Li et al. 2011**[169]	Case Report – V	1 / 0	26	Small bowel	72.0	L	Superolateral	Bone	1	D, Pro	Osteolytic	Partial	N/A	1	1	0	1	PR	0	3.0	3.0	1
**Murthy et al. 2011**[170]	Case Report – V	1 / 1	61	Breast	24.0	B	Diffuse	Muscle	0	D, IM	N/A	Biopsy	N/A	0	1, 54 Gy	Hormonal	1	PR	0	18.0	18.0	1

**Mustapha et al. 2011**[171]	Case Report – V	1 / 0	25	Liver	0	R	Superolateral	Soft Tissue	0	DV, Pro, S	N/A	FNAB	N/A	0	0	0	0	N/A	0	1.0	1.0	0
**Rosado et al. 2011**[172]	Case Report – V	1 / 0	74	Prostate	60.0	R	Superolateral	Bone	1	D, IM, Pro	Osteolytic	Biopsy	N/A	0	0	Hormonal	1	PR	0	8.0	8.0	0
**Zarogoulidis et al. 2011**[173]	Case Report – V	1 / 1	38	Lung	0	R	Posterio	Soft Tissue	0	D	N/A	FNAB	N/A	1	1, 30 Gy	0	1	N/A	0	3.0	3.0	1
**Charles et al. 2012**[174]	Case Report – V	1 / 0	24	Colorectal	7.0	L	Superior	Soft Tissue	0	Mass, Pro	N/A	Biopsy	N/A	1	1	0	1	N/A	0	3.0	3.0	1
**Chen et al. 2012**[175]	Case Report – V	1 / 1	51	Soft Tissue	72.0	L	Medial	Muscle	0	D, DV, Pro	N/A	Partial	N/A	0	0	0	1	N/A	0	12.0	12.0	1
**Dmuchowska et al. 2012**[176]	Case Report – V	1 / 1	53	Breast	0	R	Superior	Bone	1	IM, Pt	N/A	Partial	N/A	0	1	0	1	N/A	0	3.0	3.0	1
**Jiang et al. 2012**[177]	Case Report – V	1 / 0	44	Liver	17.0	B	Diffuse	Muscle	0	D, IM, Pro	N/A	Biopsy	N/A	0	1	0	1	N/A	0	3.0	3.0	1
**Jung et al. 2012**[178]	Case Report – V	1 / 0	50	Kidney	72.0	L	Medial	Soft Tissue	0	IM, Pro	Osteolytic	Complete	Exenteration	0	0	Immunothera py	1	CR	0	72.0	72.0	1
**Kim et al. 2012**[179]	Case Report – V	1 / 1	50	Breast	0	R	Diffuse	Soft Tissue	0	D, Pro, S	N/A	Biopsy	Decompressi on	1	0	Hormonal	1	N/A	0	3.0	3.0	1
**Pecen et al. 2012**[180]	Case Report – V	1 / 0	59	Liver	0	R	Posterior	Fat	0	D, IM, Pro, Pt, S	N/A	Complete	Exenteration	1	0	0	1	N/A	0	7.0	7.0	0
**SooHoo et al. 2012**[181]	Case Report – V	1 / 1	53	Bladder	1.0	R	Superolateral	Soft Tissue	0	Pt	N/A	Biopsy	N/A	0	0	0	N/A	N/A	0	3.0	3.0	0
**Tomizawa et al. 2012**[182]	Case Report – V	1 / 1	70	Breast	0	R	Posterior	Fat	0	DV, RE, S	N/A	Biopsy	N/A	0	0	0	1	N/A	0	3.0	3.0	1
**Wladis et al. 2012**[183]	Case Report – V	1 / 1	18	Soft Tissue	18.0	R	Superomedial	Fat	0	Mass, Pro	N/A	Biopsy	N/A	1	1, 30 Gy	0	1	PR	0	2.0	6.0	0
**Woo et al. 2012**[184]	Case Report – V	1 / 1	65	Small bowel	72.0	L	Lateral	Fat	0	Mass	N/A	Partial	N/A	0	0	0	1	N/A	0	6.0	6.0	1
**Dobson et al. 2013**[185]	Case Report – V	1 / 0	65	Carcinoid	9.0	L	Inferomedial	Fat	0	Mass	N/A	Partial	N/A	0	0	Radioiodine	1	PR	1	41.0	50.0	1
**Fini et al. 2013**[186]	Case Report – V	1 / 1	70	Breast	96.0	B	Diffuse	Soft Tissue	0	OP, Pt	N/A	Partial	N/A	0	0	0	1	N/A	0	3.0	3.0	1
**Fujimoto et al. 2013**[187]	Case Report – V	1 / 1	56	Gallbladder	24.0	L	Lateral	Bone	0	OP, S	Osteolytic	Biopsy	N/A	0	SRS	0	1	PR	0	12.0	12.0	1
**Patel et al. 2013**[188]	Case Report – V	1 / 0	57	Soft Tissue	24.0	L	Lateral	Soft Tissue	0	Pro, RE	N/A	Partial	N/A	1	SRS	0	0	PD	0	3.0	3.0	0
**Patel et al. 2013**[189]	Case Report – V	1 / 1	47	Breast	18.0	R	Medial	Fat	0	DV	N/A	Biopsy	N/A	N/A	N/A	N/A	N/A	N/A	0	3.0	3.0	1
**Coloma-Gonzalez et al. 2014**[190]	Case Report – V	1 / 0	56	Kidney	0	L	Lateral	Bone	0	Mass, Pt	Osteolytic	Partial	N/A	0	0	Immunothera py	0	PD	0	3.0	3.0	1
**Eldesouky et al. 2014**[191]	Case Series – IV	6 / 0	62<br>(55-70)	6 Liver (100%)	6.0<br>(0-16)	4 L (66.7%)<br>2 R (33.3%)	6 Superolateral (100%)	3 Soft Tissue (50%)<br>3 Bone (50%)	1 (16.7%)	5 Pro (83.3%)<br>2 Mass (33.3%)<br>1 IM (16.7%)<br>1 Pt (16.7%)<br>1 OP, RE, S (16.7%)	5 Osteolytic (83.3%)	N/A	N/A	6 (100%)	6 (100%)	0	N/A	N/A	0	10.0<br>(6-10)	10.0<br>(6-10)	1 (16.7%)
**Greene et al. 2014**[192]	Case Series – IV	15 / 3 (20%)	66<br>(25-84)	15 Melanoma (100%)	N/A	11 L (73.3%)<br>2 B (13.3%)<br>2 R (13.3%)	4 Medial (26.7%)<br>3 Posterior (20%)<br>3 Superior (20%)<br>2 Superomedial (13.3%)<br>1 Diffuse (6.7%)<br>1 Superolateral (6.7%)	11 Soft Tissue (73.3%)<br>4 Muscle (26.7%)	N/A	9 Pro (60%)<br>7 IM (46.7%)<br>3 Pt (20%)<br>2 DV (13.3%)<br>2 OP (13.3%)<br>1 S (6.7%)<br>1 No sympt (6.7%)	N/A	N/A	N/A	8 (53.3%)	7 (46.7%), 33 Gy	5Immunother apy (33.3%)	N/A	6 SD (40%)<br>4 PR (26.7%)<br>1 PD (6.7%)	0	15.5<br>(1-84)	15.5<br>(1-84)	3 (20%)
**Kapur et al. 2014**[193]	Case Report – V	1 / 0	79	Prostate	0	R	Superolateral	Soft Tissue	0	D, DV, Pro	N/A	Biopsy	N/A	1	0	Hormonal	1	PR	0	8.0	8.0	1

**Ludmir et al. 2014**[194]	Case Report – V	1 / 0	82	Colorectal	0	R	Superomedial	Fat	0	OP	N/A	Biopsy	N/A	0	1, 30 Gy	0	0	PD	0	6.0	6.0	0
**Saffra et al. 2014**[195]	Case Report – V	1 / 1	46	Breast	0	L	Medial	Muscle	0	D, OP, Pt, RAPD	N/A	Biopsy	N/A	1	0	Hormonal	1	PR	0	12.0	12.0	1
**Gupta et al. 2015**[196]	Case Report – V	1 / 1	52	Thyroid	0	R	Lateral	Muscle	0	D, DV, OP	N/A	Partial	N/A	0	GK, 20 Gy	0	0	PR	0	18.0	18.0	1
**Magliozzi et al. 2015**[197]	Case Series – IV	91 / 51 (56%)	N/A	36 Breast (39.6%)<br>16 CUP (<br>10 Lung<br>10 Kidney<br>4 Pharynx<br>4 Salivary<br>3 Melanoma<br>3 Pharynx<br>3 Skin<br>2 Smallbowel<br>1 Bladder<br>1 Prostate<br>1 Thyroid	45.0	50 R (54.9%)<br>41 L (45%)	N/A	N/A	N/A	68 Pro (74.7%)<br>51 IM (56%)<br>43 Pt (47.3%)<br>40 DV (44%)<br>34 Mass (37.4%)<br>30 D (33%)<br>11 OP (12.1%)<br>4 En (4.4%)	17 Osteolytic (18.7%)	30 Biopsy (33%)<br>24 FNAB (26.4%)<br>32 Partial (35.2%)<br>5 Complete (5.5%)	5Exenteration (5.5%)	57 (62.6%)	24 (26.4%)	0	N/A	N/A	0	13.5	13.5	2 (2.2%)
**Magrath et al. 2015**[198]	Case Series – IV	2 / 1 (50%)	54 (51-57)	1 Bladder (50%)<br>1 Esophagus (50%)	0.5 (0-1)	1 L (50%)<br>1 R (50%)	1 Posterior (50%)<br>1 Superior (50%)	1 Fat (50%)<br>1 Soft Tissue (50%)	1 (50%)	2 IM (100%)<br>2 Pro (100%)<br>1 DV (50%)<br>1 OP (50%)	N/A	2 Biopsy (100%)	N/A	0	1 (50%)	0	0	SD	0	3.0 (2-4)	3.0 (2-4)	1 (50%)
**Pagsisihan et al. 2015**[199]	Case Report – V	1 / 1	49	Thyroid	0	R	Superolateral	Bone	0	D, DV, Mass, Pt	Osteolytic	Biopsy	N/A	0	0	Radioiodine	1	PR	0	6.0	6.0	1
**Pearlman et al. 2015**[200]	Case Report – V	1 / 0	33	Colorectal	0	R	Superior	Soft Tissue	1	D, DV, OP, Pro	Osteolytic	Partial	Decompression	1	1	0	1	PD	1	11.0	11.0	0
**Rajabi et al. 2015**[201]	Case Report – V	1 / 0	19	Bone	20.0	L	Lateral	Soft Tissue	0	IM, Pro	Osteolytic	Complete	N/A	1	1	0	1	N/A	0	3.0	3.0	1
**Arthur et al. 2016**[202]	Case Report – V	1 / 1	27	Cervix	2.0	L	Medial	Bone	0	D, DV, Pro, RAPD	N/A	Biopsy	N/A	1	1, 30 Gy	0	1	PR	0	12.0	12.0	1
**Foster et al. 2016**[203]	Case Report – V	1 / 1	67	Colorectal	0	L	Diffuse	Muscle	0	D, Pro, RAPD	N/A	Biopsy	N/A	1	0	0	0	N/A	0	2.0	2.0	0
**MacLean et al. 2016**[204]	Case Report – V	1 / 0	39	Thymus	0	L	Medial	Soft Tissue	0	DV, IM, RAPD, S	N/A	Partial	Decompression	1	1	0	1	PR	0	2.0	2.0	1
**Pokharel et al. 2016**[205]	Case Report – V	1 / 0	66	Small bowel	0	R	Superior	Fat	0	Mass, Pro	N/A	Partial	N/A	0	0	0	1	N/A	0	2.0	2.0	1
**Tabai et al. 2016**[206]	Case Report – V	1 / 1	32	Breast	0	L	Medial	Bone	1	D, OP, Pro	N/A	Biopsy	N/A	1	0	0	0	N/A	0	2.0	2.0	0
**Tun et al. 2016**[207]	Case Report – V	1 / 0	73	Prostate	60.0	L	Lateral	Bone	1	DV, OP, Pro	Osteolytic	Complete	N/A	N/A	N/A	N/A	N/A	N/A	0	2.0	2.0	1
**AufderHeide et al. 2017**[208]	Case Report – V	1 / 0	52	Prostate	0	L	Lateral	Bone	1	Mass, OP	Osteolytic	Complete	N/A	N/A	N/A	N/A	N/A	N/A	0	2.0	2.0	1
**Geske et al. 2017**[209]	Case Series – IV	3 / 1 (33.3%)	59 (56-63)	3 Liver (100%)	0 (0-1)	2 L (66.7%)<br>1 R (33.3%)	3 Superolateral (100%)	2 Bone (66.7%)<br>1 Soft Tissue (33.3%)	1 (33.3%)	2 Mass (66.7%)<br>2 RAPD (66.7%)<br>1 OP (33.3%)<br>1 VL (33.3%)	2 Osteolytic (66.7%)	2 FNAB (66.7%)<br>1 Biopsy (33.3%)<br>1 Partial (33.3%)	1Decompression (33.3%)	0	3 (100%)	0	1 (33.3%)	2 PD (66.7%)<br>1 PR (33.3%)	0	8.0 (1-11)	8.0 (1-11)	0
**Huang et al. 2017**[210]	Case Report – V	1 / 0	64	Carcinoid	0	L	Posterior	Soft Tissue	0	DV, OP, Pt	N/A	Complete	N/A	0	0	0	1	N/A	0	6.0	6.0	0
**Marginean et al. 2017**[211]	Case Report – V	1 / 1	56	Breast	0	L	Superolateral	Muscle	0	D, DV, IM	N/A	Partial	N/A	N/A	N/A	N/A	N/A	N/A	0	1.0	1.0	1
**Nabeel et al. 2017**[212]	Case Report – V	1 / 1	48	Colorectal	0	R	Superolateral	Fat	0	Pro, Pt	N/A	Biopsy	N/A	1	1	0	1	N/A	0	7.0	7.0	0
**Nash et al. 2017**[213]	Case Report – V	1 / 1	55	Cervix	0	L	Superomedial	Soft Tissue	0	OP, Pro, RE, S	N/A	Partial	N/A	0	0	0	0	N/A	0	1.0	1.0	0

**Rakul Nambiar et al. 2017**[214]	Case Report – V	1 / 1	56	Breast	0	L	Superolateral	Soft Tissue	0	IM, Pro	N/A	Complete	N/A	0	0	Hormonal	1	N/A	0	7.0	7.0	1
**Rasool et al. 2017**[215]	Case Report – V	1 / 1	48	Soft Tissue	0	L	Posterior	Muscle	0	D, DV, OP, Pro	N/A	Complete	N/A	1	1	0	1	CR	0	18.0	18.0	1
**Wang et al. 2017**[216]	Case Report – V	1 / 0	66	Breast	72.0	R	Superomedial	Muscle	0	D, Pro	N/A	Biopsy	N/A	0	0	Hormonal	1	N/A	0	8.0	8.0	1
**Coutinho et al. 2018**[217]	Case Report – V	1 / 1	68	Breast	0	L	Superolateral	Muscle	0	D, DV Pro	N/A	Biopsy	N/A	1	0	0	1	PR	0	38.0	38.0	0
**Das et al. 2018**[218]	Case Series – IV	5 / 3 (60%)	68 (61-72)	5 Carcinoid (100%)	60 (12-244)	3 B (60%)<br>1 L (20%)<br>1 R (20%)	3 Diffuse (60%)<br>1 Lateral (20%)<br>1 Medial (20%)	5 Muscle (100%)	0	3 D (60%)<br>1 OP (20%)<br>1 Pro (20%)<br>1 S (20%)<br>1 No sympt (20%)	N/A	N/A	N/A	2 (40%)	4 (80%)	0	N/A	2 PR (40%)<br>2 SD (40%)	0	6.0 (2-120)	2.0 (2-120)	4 (80%)
**Dutton et al. 2018**[219]	Case Report – V	1 / 0	33	Testes	22.0	R	Superolateral	Soft Tissue	0	VL	Osteolytic	Partial	N/A	1	1	0	1	CR	1	9.0	48.0	1
**Gulmez-Sevim et al. 2018**[220]	Case Report – V	1 / 1	69	Thyroid	0	R	Superolateral	Bone	1	D, DV, RAPD	Osteolytic	Complete	N/A	0	0	Radioiodine	N/A	N/A	0	3.0	3.0	1
**Nifosi et al. 2018**[221]	Case Report – V	1 / 1	46	Breast	34.0	L	Medial	Muscle	0	D, IM, OP, Pro	N/A	FNAB	N/A	1	SRS, 30 Gy	0	1	PR	0	3.0	3.0	1
**Pinto-Proenca et al. 2018**[222]	Case Report – V	1 / 1	66	Breast	0	L	Inferomedial	Soft Tissue	0	Mass	N/A	Partial	N/A	1	0	Hormonal	1	PR	0	60.0	60.0	1
**Ramsey et al. 2018**[223]	Case Report – V	1 / 0	71	Soft Tissue	30.0	L	Superomedial	Soft Tissue	0	D, Pt	N/A	Complete	Exenteration	1	1, 64 Gy	Immunotherapy	N/A	N/A	0	29.0	29.0	1
**Roelofs et al. 2018**[224]	Case Report – V	1 / 1	66	Small bowel	84.0	L	Superior	Muscle	0	DV, Pro	N/A	Complete	N/A	1	0	0	1	PR	1	8.0	9.0	1
**Rong et al. 2018**[225]	Case Report – V	1 / 0	51	Pancreas	0	L	Lateral	Muscle	0	D, DV, RAPD	N/A	Partial	Decompression	N/A	N/A	N/A	N/A	N/A	0	3.0	3.0	1
**Shaikh et al. 2018**[226]	Case Report – V	1 / 0	58	Breast	0	R	Diffuse	Sift Tissue	0	DV, Mass, OP	N/A	Biopsy	N/A	1	0	0	N/A	N/A	0	3.0	3.0	1
**Chumdermpadetsuk et al. 2019**[227]	Case Report – V	1 / 0	22	Kidney	0	R	Superolateral	Bone	1	D, DV, OP	Osteolytic	Complete	N/A	1	SRS	0	1	CR	0	6.0	6.0	1
**Crisostomo et al. 2019**[228]	Case Report – V	1 / 0	56	Lung	10.0	L	Medial	Muscle	0	D, Pro, RAPD, RE	N/A	Biopsy	N/A	0	0	0	0	N/A	0	3.0	3.0	0
**Cruzado-Sanchez et al. 2019**[229]	Case Report – V	1 / 1	60	Lung	0	R	Superior	Fat	0	IM, Mass, Pro	N/A	FNAB	N/A	N/A	N/A	N/A	N/A	N/A	0	1.0	1.0	1
**Espinosa-Barberi et al. 2019**[230]	Case Series – IV	2 / 1	66 (63-69)	1 Breast (50%)<br>1 Lung (50%)	6.0 (0-12)	1 B (50%)<br>1 R (50%)	1 Lateral (50%)<br>1 Superolateral (50%)	1 Bone (50%)<br>1 Muscle (50%)	0	1 D, Pro, VL (50%)<br>1 D, IM, Pro (50%)	1 Osteolytic (50%)	2 FNAB (100%)	N/A	1 (50%)	1 (50%)	1 Hormonal (50%)	1 (50%)	1 PD (50%)	0	2.5 (2-3)	2.5 (2-3)	1 (50%)
**Framarino-dei-Malatesta et al. 2019**[231]	Case Report – V	1 / 1	44	Breast	84.0	R	Inferior	Muscle	0	D, DV, IM	N/A	Biopsy	N/A	1	SRS, 40 Gy	Hormonal	1	PR	0	20.0	20.0	1
**Hernandez Pardines et al. 2019**[232]	Case Report – V	1 / 1	58	Breast	0	L	Lateral	Muscle	0	D, IM	N/A	Biopsy	N/A	1	0	0	0	SD	0	2.0	2.0	1
**Kagusa et al. 2019**[233]	Case Report – V	1 / 0	64	Cervix	7.0	R	Medial	Soft Tissue	0	D, IM, Pro	N/A	Complete	N/A	1	1, 60 Gy	0	1	PR	0	2.0	2.0	0
**Kiziloglu et al. 2019**[234]	Case Report – V	1 / 1	63	Breast	12.0	R	Medial	Muscle	0	D, IM	N/A	Biopsy	N/A	1	1, 45 Gy	0	1	PR	0	15.0	15.0	1
**Kluska et al. 2019**[235]	Case Report – V	1 / 1	63	Breast	132.0	L	Lateral	Muscle	0	VL	N/A	Partial	N/A	0	0	Hormonal	0	N/A	0	2.0	2.0	1
**Kozubowska et al. 2019**[236]	Case Report – V	1 / 0	73	Carcinoid	0	R	Inferomedial	Soft Tissue	0	D, IM, OP, RE, S	N/A	Partial	Decompression	0	0	Hormonal	1	N/A	0	4.0	4.0	1
**Rider et al. 2019**[237]	Case Series – IV	2 / 1	68 (49-87)	Adrenal Gland	12.5 (1-24)	2 R (100%)	2 Diffuse (100%)	2 Soft Tissue (100%)	2 (100%)	2 Mass (100%)<br>1 Pro (50%)<br>1 S (50%)	2 Osteolytic (100%)	1 Complete (50%)<br>1 Partial (50%)	1Decompression (50%)	0	1 (50%)	0	2 (100%)	1 PR (50%)	0	2.0 (1-3)	2.0 (1-3)	1 (50%)
**Sochat et al. 2019**[238]	Case Report – V	1 / 0	60	Liver	0	R	Lateral	Bone	0	D, Mass, Pro	Oncolytic	Partial	N/A	1	1, 37.5 Gy	0	1	CR	0	18.0	18.0	1

**Tao et al. 2019**[239]	Case Report – V	1 / 1	63	Breast	6.0	L	Superolateral	Soft Tissue	1	D	N/A	Complete	N/A	0	1, 52.5 Gy	Hormonal	1	CR	0	6.0	6.0	1
**Yadav et al. 2019**[240]	Case Report – V	1 / 0	60	Bladder	0	L	Superomedial	Muscle	0	DV, OP, Pro, RE	N/A	Complete	N/A	0	0	0	1	N/A	0	6.0	6.0	1
**Yu et al. 2019**[241]	Case Report – V	1 / 1	43	Small bowel	48.0	L	Superolateral	Soft Tissue	0	Pro, VL	N/A	Complete	N/A	0	0	0	1	N/A	0	6.0	6.0	1
**Bacorn et al. 2020**[242]	Case Report – V	1 / 1	51	Colorectal	8.0	R	Superior	Fat	0	Pt, S	N/A	Biopsy	N/A	1	0	0	0	SD	0	6.0	6.0	0
**Blohmer et al. 2020**[243]	Case Series – IV	15 / 15 (100%)	N/A	15 Breast (100%)	26.0 (0-153.8)	7 R (46.7%)<br>5 L (33.3%)<br>3 B (20%)	N/A	5 Bone (33.3%)<br>5 Muscle (33.3%)<br>5 Fat (33.3%)	N/A	N/A	N/A	15 Surgery (100%)	N/A	14 (93.3%)	9 (60%)	0	N/A	N/A	0	2.0 (0.5-91.3)	2.0 (0.5-91.3)	2 (13.3%)
**El Bakraoui et al. 2020**[244]	Case Report – V	1 / 1	43	Breast	3.0	L	Superolateral	Soft Tissue	0	DV, Mass	Osteolytic	Biopsy	N/A	1	1, 60 Gy	0	1	PR	0	14.0	14.0	0
**Halliday et al. 2020**[245]	Case Report – V	1 / 0	79	Carcinoid	36.0	B	Diffuse	Muscle	0	D, Pro	N/A	Biopsy	N/A	1	1, 30 Gy	Hormonal	1	PR	0	12.0	12.0	1
**Hoenck et al. 2020**[246]	Case Report – V	1 / 1	58	Carcinoid	0	B	Diffuse	Muscle	0	DV, IM, Pt, RAPD, S	N/A	Biopsy	N/A	0	0	Hormonal	1	N/A	0	2.0	2.0	0
**Long et al. 2020**[247]	Case Report – V	1 / 0	84	Colorectal	0	R	Superolateral	Bone	1	Pro, Pt, RAPD	Osteolytic	Partial	N/A	1	1	0	N/A	N/A	0	3.0	3.0	1
**Madabhavi et al. 2020**[248]	Case Report – V	1 / 0	72	Liver	0	L	Superolateral	Bone	1	Mass, OP, Pro	Osteolytic	FNAB	N/A	1	0	0	1	PR	0	7.0	7.0	1
**Mahuvakar et al. 2020**[249]	Case Report – V	1 / 1	72	Kidney	96.0	R	Medial	Soft Tissue	0	D, IM, Pro	N/A	Complete	N/A	0	0	0	1	CR	0	6.0	6.0	1
**Marotta et al. 2020**[250]	Case Report – V	1 / 1	58	Breast	240.0	B	Superomedial	Muscle	0	D, Pt	N/A	Biopsy	N/A	1	1, 45 Gy	0	1	PR	0	3.0	3.0	1
**Mian et al. 2020**[251]	Case Report – V	1 / 0	74	Mesothelium	18.0	L	Inferior	Muscle	0	Pro, RAPD	N/A	Partial	N/A	1	1, 25 Gy	0	1	PR	0	5.0	5.0	1
**Montejano-Milner et al. 2020**[252]	Case Series – IV	10 / 5 (50%)	62.5 (43-82)	4 Breast (40%)<br>2 Bladder (20%)<br>2 Lung (20%)<br>1 Ovary (10%)<br>1 Pharynx (10%)	N/A	5 L (50%)<br>4 R (40%)<br>1 B (10%)	5 Posterior (50%)<br>3 Diffuse (30%)<br>1 Inferolateral (10%)<br>1 Superior (10%)	4 Soft Tissue (40%)<br>3 Muscle (30%)<br>2 Fat (20%)<br>1 Bone (10%)	N/A	5 D (50%)<br>3 DV (30%)<br>3 Mass (30%)<br>3 OP (30%)<br>3 Pro (30%)<br>1 En (10%)<br>1 RE (10%)<br>1 S (10%)	N/A	8 Surgery (100%)<br>1 Complete (10%)<br>2 Biopsy (20%)	N/A	3 (30%)	3 (30%)	1 Hormonal (10%)	N/A	N/A	0	5.5 (1-54)	5.5 (1-54)	1 (10%)
**Narayanan et al. 2020**[253]	Case Report – V	1 / 1	52	Melanoma	0	R	Lateral	Soft Tissue	0	IM, Pro	N/A	Biopsy	N/A	1	0	0	0	N/A	0	3.0	3.0	0
**Pastore et al. 2020**[254]	Case Report – V	1 / 0	72	Prostate	0	L	Superomedial	Soft Tissue	1	DV, Pro	Osteolytic	FNAB	N/A	0	0	Hormonal	1	N/A	0	18.0	18.0	0
**Protopapa et al. 2020**[255]	Case Report – V	1 / 0	53	Liver	0	L	Superomedial	Bone	1	Mass, VL	Osteolytic	Biopsy	N/A	0	1	0	0	N/A	0	3.0	3.0	1
**Sindoni et al. 2020**[256]	Case Series – IV	28 / 28 (100%)	56.3	28 Breast (100%)	60.0	18 L (64.3%)<br>10 R (35.7%)	N/A	N/A	N/A	14 Pro (50%)<br>4 D (14.3%)<br>4 Mass (14.3%)<br>2 En (7.1%)<br>2 Pt (7.1%)<br>2 S (7.1%)	N/A	28 Biopsy (100%)	N/A	26 (92.8%)	12 (42.8%)	0	N/A	N/A	0	26.4	26.4	18 (64.3%)
**Tsuruta et al. 2020**[257]	Case Report – V	1 / 1	60	Stomach	168.0	L	Superomedial	Soft Tissue	0	D, OP, VL	N/A	Biopsy	N/A	0	0	0	1	N/A	0	12.0	12.0	1
**Wada et al. 2020**[258]	Case Report – V	1 / 0	79	Prostate	0	L	Superomedial	Soft Tissue	0	DV, Pt	N/A	Biopsy	N/A	0	0	Hormonal	1	PR	0	3.0	3.0	1
**El-Khazen Dupuis et al. 2021**[259]	Case Series – IV	2 / 2	75 (74-76)	2 Breast (100%)	0	1 B (50%)<br>1 L (50%)	1 Diffuse (50%)<br>1 Posterior (50%)	1 Muscle (50%)<br>1 Soft Tissue (50%9	0	2 D (100%)<br>2 En (100%)<br>1 DV, IM, Mass, Pro (50%)	N/A	1 Biopsy (50%)	N/A	1 (50%)	0	1 Hormonal (50%)	1 (50%)	1 PR (50%)	0	37.5 (3-72)	37.5 (3-72)	1 (50%)

**Mendia et al. 2021**[260]	Case Report – V	1 / 1	72	Cervix	1.0	L	Lateral	Fat	0	D, OP	N/A	Biopsy	N/A	1	1, 30 Gy	0	1	PD	0	4.0	4.0	0
**Oprean et al. 2021**[261]	Case Report – V	1 / 1	57	Breast	0	L	Diffuse	Muscle	0	D, DV, Pro, Pt	N/A	Partial	N/A	0	1, 25 Gy	Hormonal	1	CR	1	46.0	53.0	0
**Razem et al. 2021**[262]	Case Report – V	1 / 1	33	Breast	1.0	L	Diffuse	Muscle	0	D, IM	N/A	Biopsy	N/A	0	0	Hormonal	1	CR	0	12.0	12.0	1

Abbreviations: **M**, Male; **F**, Female; **B**, bilateral; **L**, left; **Mid**, midline; **R**, right; **CUP**, carcinoma of unknown primary; **D**, diplopia; **DV**, decreased vision; **En**, enophthalmos; **IM**, impaired eye motility; **OP**, orbital pain; **Pro**, proptosis; **Pt**, ptosis; **RAPD**, relative afferent pupillary defect (cranial nerve III deficit); **RE**, red eye; **S**, swelling; **VL**, vision loss; **FNAB**, fine needle aspiration biopsy; **Complete**, resection 100% tumor; **Partial**, resection <100% tumor; **CK**, cyber-knife; **GK**, gamma-knife; **SRS**, stereotactic radiosurgery; **WBRT**, whole brain radiation therapy; **LITT**, laser interstitial thermal therapy; **CR**, Complete Response, **PR**, Partial Response, **SD**, Stable Disease; **PD**, Progression Disease; **PFS**, Progression-Free Survival; **OS**, Overall Survival; **N/A**, Not Available.

References

1. Arnott EJ, Greaves DP. METASTASES IN THE ORBIT. *Br J Ophthalmol*. 1965;49(1):43-45. doi:10.1136/bjo.49.1.43
2. Chatterjee BM, Deb M. Metastatic Carcinoma of the Orbit. *Am J Ophthalmol*. 1965;59(1):103-105. doi:10.1016/0002-9394(65)95028-2
3. Mortada A. Roentgenography in Orbital Metastases by Exophthalmos. *Am J Ophthalmol*. 1968;65(1):48-53. doi:10.1016/0002-9394(68)91027-1
4. Honrubia FM, Davis WH, Moore MK, Elliott JH. Carcinoid Syndrome with Bilateral Orbital Metastases. *Am J Ophthalmol*. 1971;72(6):1118-1121. doi:10.1016/0002-9394(71)91218-9
5. Ashton N, Morgan G. Discrete carcinomatous metastases in the extraocular muscles. *Br J Ophthalmol*. 1974;58(2):112-117. doi:10.1136/bjo.58.2.112
6. HUH SH, NISCE LZ, SIMPSON LD, CHU FCH. VALUE OF RADIATION THERAPY IN THE TREATMENT OF ORBITAL METASTASIS. *Am J Roentgenol*. 1974;120(3):589-594. doi:10.2214/ajr.120.3.589
7. Saxena RB, Mathur RN, Sonani SZ. Orbital metastasis of mixed parotid tumour. *Indian J Ophthalmol*. 1975;23(2):23-24. http://www.ncbi.nlm.nih.gov/pubmed/1236448
8. Usui T, Ishibe T, Nihira H. Orbital Metastasis from Prostatic Carcinoma. *Br J Urol*. 1975;47(4):458-458. doi:10.1111/j.1464-410X.1975.tb04007.x
9. Balestrazzi E. An Unusual Case of Metastatic Malignant Melanoma in the Orbit. *Ophthalmologica*. 1976;172(1):70-74. doi:10.1159/000307587
10. Howard GM, Jakobiec FA, Trokel SL, Iwamoto T, Jones IS. Pulsating metastatic tumor of the orbit. *Am J Ophthalmol*. 1978;85(6):767-771. doi:10.1016/s0002-9394(14)78103-6
11. Stewart WB, Newman NM, Cavender JC, Spencer WH. Fibrous Histiocytoma Metastatic to the Orbit. *Arch Ophthalmol*. 1978;96(5):871-873. doi:10.1001/archopht.1978.03910050473016
12. Bullock JD, Yanes B. Ophthalmic Manifestations of Metastatic Breast Cancer. *Ophthalmology*. 1980;87(10):961-973. doi:10.1016/S0161-6420(80)35124-5
13. Lubin JR, Grove AS, Zakov ZN, Albert DM. Hepatoma Metastatic to the Orbit. *Am J Ophthalmol*. 1980;89(2):268-273. doi:10.1016/0002-9394(80)90123-3
14. Rush JA, Waller RR, Campbell RJ. Orbital Carcinoid Tumor Metastatic from the Colon. *Am J Ophthalmol*. 1980;89(5):636-640. doi:10.1016/0002-9394(80)90279-2
15. SOBOL S, DRUCK NS, WOLF M. PALLIATIVE ORBITAL DECOMPRESSION FOR METASTATIC MELANOMA TO THE ORBIT. *Laryngoscope*. 1980;90(2):329???333. doi:10.1288/00005537-198002000-00021
16. Rush JA, Older JJ, Richman A V. Testicular Seminoma Metastatic to the Orbit. *Am J Ophthalmol*. 1981;91(2):258-260. doi:10.1016/0002-9394(81)90185-9
17. Winkler CF. Orbital Metastasis From Prostatic Carcinoma. *Arch Ophthalmol*. 1981;99(8):1406. doi:10.1001/archopht.1981.03930020280016
18. Wolter JR, Hendrix RC. Osteoblastic Prostate Carcinoma Metastatic to the Orbit. *Am J Ophthalmol*. 1981;91(5):648-651. doi:10.1016/0002-9394(81)90068-4
19. Zubler MA, Rivera R, Lane M. Hepatoma presenting as a retro-orbital metastasis. *Cancer*. 1981;48(8):1883-1885. doi:10.1002/1097-0142(19811015)48:8<1883::AID-CNCR2820480828>3.0.CO;2-J
20. Carriere VM, Karcioglu ZA, Apple DJ, Insler MS. A Case of Prostate Carcinoma with Bilateral Orbital Metastases and the Review of the Literature. *Ophthalmology*. 1982;89(4):402-406. doi:10.1016/S0161-6420(82)34779-X
21. Riddle PJ, Font RL, Zimmerman LE. Carcinoid tumors of the eyeand orbit: A clinicopathologic study of 15 cases, with histochemical and electron microscopic observations. *Hum Pathol*. 1982;13(5):459-469. doi:10.1016/S0046-8177(82)80029-4
22. Tertzakian GM, Herr HW, Mehta MB. Orbital metastases from prostatic carcinoma. *Urology*. 1982;19(4):427-429. doi:10.1016/0090-4295(82)90205-9
23. Weitberg AB, Weitzman SA. Metastatic Islet Cell Carcinoma: A Potentially Treatable Cause of "Carcinoma of Unknown Origin." *CA Cancer J Clin*. 1983;33(3):167-171. doi:10.3322/canjclin.33.3.167
24. Mortada A. Binocular diplopia due to metastasis in orbital muscle from female breast cancer years after radical mastectomy. *Orbit*. 1984;3(1):71-73. doi:10.3109/01676838409028657
25. Reifler DM, Kini SR, Liu D, Littleton RH. Orbital metastasis from prostatic carcinoma. Identification by immunocytology. *Arch Ophthalmol (Chicago, Ill 1960)*. 1984;102(2):292-295. doi:10.1001/archopht.1984.01040030236033
26. DUNAWAY RL. Ptosis as the Presenting Sign of Orbital Involvement from Metastatic Bronchogenic Carcinoma. *Optom Vis Sci*. 1985;62(12):908-912. doi:10.1097/00006324-198512000-00013
27. Bond J, Wesley RE, Reynolds VH, Elliott JH, Glick AD. Orbital Metastasis From Cutaneous Melanoma. *South Med J*. 1986;79(11):1439-1442. doi:10.1097/00007611-198611000-00029
28. Denby P, Harvey L, English MG. Solitary metastasis from an occult renal cell carcinoma presenting as a primary lacrimal gland tumour. *Orbit*. 1986;5(1):21-24. doi:10.3109/01676838609034231
29. Schlaen ND, Naves AE. Orbital and Choroidal Metastases From Carcinoma of the Male Breast. *Arch Ophthalmol*. 1986;104(9):1344-1346. doi:10.1001/archopht.1986.01050210098033
30. Hornblass A, Kass LG, Reich R. Thyroid Carcinoma Metastatic to the Orbit. *Ophthalmology*. 1987;94(8):1004-1007. doi:10.1016/S0161-6420(87)33353-6
31. Kaltreider SA, Destro M, Lemke BN. Leiomyosarcoma of the Orbit. *Ophthalmic Plast Reconstr Surg*. 1987;3(1):35-42. doi:10.1097/00002341-198701000-00008
32. Noci ND, Mininni F, Pomes L. A clinical case of breast adenocarcinoma metastatic to the orbital plate of the frontal bone. *Orbit*. 1987;6(2):149-150. doi:10.3109/01676838709036093
33. Oosterhuis JA, de Keizer RJW, de Wolff-Rouendaal D, Kakebeeke-Kemme HM, de Graaff ML. Ocular and orbital metastasis of cutaneous melanomas. *Int Ophthalmol*. 1987;10(3):175-184. doi:10.1007/BF00139345
34. Otto RA, Templer JW, Renner G, Hurt M. Secondary and metastatic tumors of the orbit. *Otolaryngol Head Neck Surg*. 1987;97(3):328-334. doi:10.1177/019459988709700315
35. Shields CL, Shields JA, Eagle RC, Peyster RG, Conner BE, Green HA. Orbital Metastasis From a Carcinoid Tumor: Computed Tomography, Magnetic Resonance Imaging, and Electron Microscopic Findings. *Arch Ophthalmol*. 1987;105(7):968-971. doi:10.1001/archopht.1987.01060070104038
36. Stefanyszyn MA, DeVita EG, Flanagan JC. Breast Carcinoma Metastatic to the Orbit. *Ophthalmic Plast Reconstr Surg*. 1987;3(1):43-48. doi:10.1097/00002341-198701000-00009
37. Boldt HC, Nerad JA. Orbital Metastases From Prostate Carcinoma. *Arch Ophthalmol*. 1988;106(10):1403-1408. doi:10.1001/archopht.1988.01060140567024
38. Orcutt JC, Char DH. Melanoma Metastatic to the Orbit. *Ophthalmology*. 1988;95(8):1033-1037. doi:10.1016/S0161-6420(88)33061-7
39. Jacobs M, Benger R. Metastatic breast carcinoma of the orbit. *Aust N Z J Ophthalmol*. 1989;17(4):357-361. doi:10.1111/j.1442-9071.1989.tb00554.x
40. Shields CL, Shields JA, Peggs M. Tumors Metastatic to the Orbit. *Ophthalmic Plast Reconstr Surg*. 1988;4(2):73-80. doi:10.1097/00002341-198804020-00003
41. Malviya VK, Blessed W, Lawrence WD, Deppe G. Retroorbital metastases in ovarian cancer. *Gynecol Oncol*. 1989;35(1):120-123. doi:10.1016/0090-8258(89)90025-5
42. Ruusuvaara P, Setälä K, Tarkkanen A. Orbital metastasis from cutaneous malignant melanoma. *Acta Ophthalmol*. 2009;67(3):325-328. doi:10.1111/j.1755-3768.1989.tb01881.x
43. Spaide RF, Granger E, Hammer BD, Negron FJ, Paglen PG. Rapidly expanding exophthalmos: an unusual presentation of small cell lung cancer. *Br J Ophthalmol*. 1989;73(6):461-462. doi:10.1136/bjo.73.6.461
44. Capone A. Discrete Metastasis of Solid Tumors to Extraocular Muscles. *Arch Ophthalmol*. 1990;108(2):237. doi:10.1001/archopht.1990.01070040089037
45. Friedman J, Karesh J, Rodrigues M, Sun C-CJ. Thyroid Carcinoma Metastatic to the Medial Rectus Muscle. *Ophthalmic Plast Reconstr Surg*. 1990;6(2):122-125. doi:10.1097/00002341-199006000-00008
46. Goldberg RA, Rootman J. Clinical Characteristics of Metastatic Orbital Tumors. *Ophthalmology*. 1990;97(5):620-624. doi:10.1016/S0161-6420(90)32534-4
47. Shetlar DJ, Font RL, Ordóñez N, El-Naggar A, Boniuk M. A Clinicopathologic Study of Three Carcinoid Tumors Metastatic to the Orbit. *Ophthalmology*. 1990;97(2):257-264. doi:10.1016/S0161-6420(90)32617-9
48. Wakisaka S, Tashiro M, Nakano S, Kita T, Kisanuki H, Kinoshita K. Intracranial and orbital metastasis of hepatocellular carcinoma. *Neurosurgery*. Published online May 1990:863. doi:10.1097/00006123-199005000-00021
49. Felip E, Rovirosa MA, Salud A, Capdevila F, Bellmunt J, Giralt J. Orbital metastases from transitional-cell cancer of the urinary bladder. *Urol Int*. 1991;46(1):82-84. doi:10.1159/000281784
50. Glazer LC, Harris GJ, Simons KB. Orbital Metastasis as the Presenting Sign of Adenocarcinoma of the Breast. *Ophthalmic Plast Reconstr Surg*. 1991;7(4):252-255. doi:10.1097/00002341-199112000-00003
51. van der Heijden A, Twijnstra A, Lamers WPMA, Hupperets PSGJ, Freling G. An unusual cause of diplopia in a cancer patient. *Eur J Cancer Clin Oncol*. 1991;27(10):1315-1316. doi:10.1016/0277-5379(91)90104-L
52. Zucker JL. Mycosis Fungoides Metastatic to the Orbit. *Arch Ophthalmol*. 1991;109(5):688. doi:10.1001/archopht.1991.01080050102038
53. Brackup AB, Nerad JA, Carter KD. Orbital metastasis from a malignant melanoma arising in a giant congenital melanocytic nevus. *Orbit*. 1992;11(3):123-130. doi:10.3109/01676839209074008
54. Di Leo A, Bajetta E. Adenocarcinoma of Unknown Primary Site with Bone Marrow and Orbital Metastases Suggestive of a Prostatic Origin. *Tumori J*. 1992;78(1):63-64. doi:10.1177/030089169207800116
55. Shetty B, Tyers AG. Metastatic carcinoma in the orbit. A case report. *Indian J Ophthalmol*. 1992;40(1):29-30. http://www.ncbi.nlm.nih.gov/pubmed/1464453
56. Tijl J, Koornneef L, Eijpe A, Thomas L, Gonzalez Gonzalez D, Veenhof C. Metastatic tumors to the orbit — management and prognosis. *Graefe's Arch Clin Exp Ophthalmol*. 1992;230(6):527-530. doi:10.1007/BF00181773
57. Vanderpump MPJ, Tunbridge WMG. Hürthle cell carcinoma presenting with retroorbital metastasis. *J R Soc Med*. 1992;85(8):493-494. http://www.ncbi.nlm.nih.gov/pubmed/1404203
58. Bersani TA, Costello JJ, Mango CA, Streeten BW. Benign Approach to a Malignant Orbital Tumor. *Ophthalmic Plast Reconstr Surg*. 1994;10(1):42-44. doi:10.1097/00002341-199403000-00008
59. Ellis M, Drewe R, O'Day J. Desmoplastic malignant melanoma presenting with orbital involvement. *Aust N Z J Ophthalmol*. 1994;22(2):119-123. doi:10.1111/j.1442-9071.1994.tb00778.x

60. Hugkulstone CE, Winder S, Sokal M. Bilateral orbital metastases from transitional cell carcinoma of the bladder. *Eye (Lond)*. 1994;8 (Pt 5):580-582. doi:10.1038/eye.1994.141
61. Loo KT, Tsui WMS, Ho LC, Tang SK, Tse CH, Chung KH. Hepatocellular carcinoma metastasizing to the brain and orbit: report of three cases. *Pathology*. 1994;26(2):119-122. doi:10.1080/00313029400169321
62. Tranfa F, Cennamo G, Rosa N, de Rosa G, Bonavolonià G. An Unusual Orbital Lesion: Hepatoma Metastatic to the Orbit. *Ophthalmologica*. 1994;208(6):329-332. doi:10.1159/000310532
63. Aburn NS, Whitehead K, Sullivan TJ. Bronchopulmonary atypical carcinoid tumour metastatic to the orbit. *Aust N Z J Ophthalmol*. 1995;23(3):241-244. doi:10.1111/j.1442-9071.1995.tb00166.x
64. Airoldi M, Succo G, Valente G, Cavalot A, Gabriele P, Bumma C. Head and Neck Metastases of Renal Cancer after Nephrectomy: A Report of 2 Cases. *Tumori J*. 1995;81(3):213-214. doi:10.1177/030089169508100313
65. Day TA, Hoasjoe DK, Hebert RL, et al. Head and Neck Squamous Cell Carcinoma Metastatic to the Orbital Apex. *Skull Base*. 1995;5(02):123-129. doi:10.1055/s-2008-1058943
66. FAN JT, BUETTNER H, BARTLEY GB, BOLLING JP. Clinical Features and Treatment of Seven Patients With Carcinoid Tumor Metastatic to the Eye and Orbit. *Am J Ophthalmol*. 1995;119(2):211-218. doi:10.1016/S0002-9394(14)73875-9
67. Rhatigan MC, Ashworth JL, Shah S, Bonshek RE, Leatherbarrow B. Bilateral orbital metastases from breast carcinoma masquerading as thyroid eye disease. *Eye*. 1995;9(5):653-655. doi:10.1038/eye.1995.161
68. Thomas KM, Cumberworth VL, McEwan J. Orbital and skin metastases in a polymorphous low grade adenocarcinoma of the salivary gland. *J Laryngol Otol*. 1995;109(12):1222-1225. doi:10.1017/S0022215100132517
69. Burnstine MA, Frueh BR, Elner VM. Angiosarcoma metastatic to the orbit. *Arch Ophthalmol (Chicago, Ill 1960)*. 1996;114(1):93-96. doi:10.1001/archopht.1996.01100130089017
70. El-Toukhy E, Levine MR, Abdul-Karim FW, Larson DW. Carcinoid Tumors of the Orbit. *Ophthalmic Plast Reconstr Surg*. 1996;12(4):279-283. doi:10.1097/00002341-199612000-00011
71. Char DH, Miller T, Kroll S. Orbital metastases: diagnosis and course. *Br J Ophthalmol*. 1997;81(5):386-390. doi:10.1136/bjo.81.5.386
72. Fezza J, Sinard J. Metastatic Liposarcoma to the Orbit. *Am J Ophthalmol*. 1997;123(2):271-272. doi:10.1016/S0002-9394(14)71055-4
73. Hayashi N, Iwata J, Masaoka N, Ueno H, Ohtsuki Y, Moriki T. Ameloblastoma of the mandible metastasizing to the orbit with malignant transformation. A histopathological and immunohistochemical study. *Virchows Arch*. 1997;430(6):501-507. doi:10.1007/s004280050061
74. Logrono R, Inhorn SL, Dortzbach RK, Kurtycz DF. Leiomyosarcoma metastatic to the orbit: Diagnosis by fine-needle aspiration. *Diagn Cytopathol*. 1997;17(5):369-373. doi:10.1002/(SICI)1097-0339(199711)17:5<369::AID-DC10>3.0.CO;2-L
75. Mezer E, Gdal-On M, Miller B. Orbital metastasis of renal cell carcinoma masquerading as Amaurosis fugax. *Eur J Ophthalmol*. 2008;7(3):301-304. doi:10.1080/01676830802316688
76. Sekundo W, Vogel J. Orbital tumour as a presenting symptom of breast carcinoma: Value of detecting hormone receptors. *Eye*. 1997;11(4):560-563. doi:10.1038/eye.1997.143
77. Zambarakji HJ, Simcock PR, Kinnear PE. Bilateral Orbital Metastases in a Woman with Breast Carcinoma. *J R Soc Med*. 1997;90(12):684-684. doi:10.1177/014107689709001214
78. Font RL. Hepatocellular Carcinoma Metastatic to the Orbit. *Arch Ophthalmol*. 1998;116(7):942. doi:10.1001/archopht.116.7.942
79. Garcia GH, Weinberg DA, Glasgow BJ, Hunt KE, Venegas R, Goldberg RA. Carcinoma of the Male Breast Metastatic to Both Orbits. *Ophthalmic Plast Reconstr Surg*. 1998;14(2):130-133. doi:10.1097/00002341-199803000-00010
80. Schaefer-Hirschkind E, Madonna RJ. Renal cell carcinoma metastatic to the orbit. *Clin Eye Vis Care*. 1998;10(2):99-101. doi:10.1016/S0953-4431(98)00009-5
81. Toller KK, Gigantelli JW, Spalding MJ. Bilateral orbital metastases from breast carcinoma. *Ophthalmology*. 1998;105(10):1897-1901. doi:10.1016/S0161-6420(98)91037-5
82. Collins MJ, Wojno TH, Grossniklaus HE. Metastatic esophageal carcinoma to the orbit. *Am J Ophthalmol*. 1999;127(2):228-229. doi:10.1016/S0002-9394(98)00307-9
83. Scolyer RA, Painter DM, Harper CG, Soon Lee C. Hepatocellular carcinoma metastasizing to the orbit diagnosed by fine needle aspiration cytology. *Pathology*. 1999;31(4):350-353. doi:10.1080/003130299104710
84. Wein FB, Perry JD, Miller NR, Brinker DA, Leopold DA. Pulmonary carcinoid tumor presenting with simultaneous orbital and intracranial metastases: value of transnasal endoscopic orbital biopsy and decompression. *Orbit*. 1999;18(4):267-272. doi:10.1076/orbi.18.4.267.2687
85. Wolstencroft SJ. Orbital Metastasis Due to Interval Lobular Carcinoma of the Breast. *Arch Ophthalmol*. 1999;117(10):1419. doi:10.1001/archopht.117.10.1419
86. Callejo SA, Kronish JW, Decker SJ, Cohen GR, Rosa RH. Malignant granular cell tumor metastatic to the orbit. *Ophthalmology*. 2000;107(3):550-554. doi:10.1016/S0161-6420(99)00135-9
87. Daumerie C, De Potter P, Godfraind C, Rahier J, Jamar F, Squifflet JP. Orbital Metastasis as Primary Manifestation of Thyroid Carcinoma. *Thyroid*. 2000;10(2):189-192. doi:10.1089/thy.2000.10.189
88. Friedrich RE, Donath K. Epithelial-myoepithelial carcinoma of the parotid gland with multiple distant metastases: A case report. *J Oral Maxillofac Surg*. 2000;58(6):ajoms0580690. doi:10.1053/joms.2000.6223
89. Stuntz M, Yamini D, Moss J, Klein S, Khalkhali I. Bilateral Orbital Metastases as the Presenting Finding in a Male Patient with Breast Cancer: A Case Report and Review of the Literature. *Breast J*. 2000;6(3):204-208. doi:10.1046/j.1524-4741.2000.97090.x
90. Bartley GB, Campbell RJ, Salomão DR, Bradley EA, Marsh WR, Bite U. Adrenocortical Carcinoma Metastatic to the Orbit. *Ophthal Plast Reconstr Surg*. 2001;17(3):215-220. doi:10.1097/00002341-200105000-00012
91. Shields JA, Shields CL, Brotman HK, Carvalho C, Perez N, Eagle RC. Cancer Metastatic to the Orbit. *Ophthal Plast Reconstr Surg*. 2001;17(5):346-354. doi:10.1097/00002341-200109000-00009
92. Maheshwari GK, Baboo HA, Gopal U, Wadhwa MK. Carcinoma of the tongue presenting as orbital metastasis. *Turkish J Cancer*. 2002;32(4):172-176.
93. McCulley TJ, Yip C-C, Bullock JD, Warwar RE, Hood DL. Cervical Carcinoma Metastatic to the Orbit. *Ophthalmic Plast Reconstr Surg*. 2002;18(5):385-387. doi:10.1097/00002341-200209000-00013
94. Misra A, Misra S, Chaturvedi A, Srivastava PK. Orbital metastasis from gall bladder carcinoma. *Br J Radiol*. 2002;75(889):72-73. doi:10.1259/bjr.75.889.750072
95. Baltogiannis D, Kalogeropoulos C, Ioachim E, Agnantis N, Psilas K, Giannakopoulos X. Orbital Metastasis from Prostatic Carcinoma. *Urol Int*. 2003;70(3):219-222. doi:10.1159/000068753
96. Baroody M, Hartstein ME, Holds JB. Immunotherapy for Melanoma Metastatic to the Orbit. *Ophthalmic Plast Reconstr Surg*. 2003;19(4):270-274. doi:10.1097/01.IOP.0000075015.65210.B4
97. Fynn-Thompson N, McKiernan JM, Fay A. Transitional Cell Carcinoma of the Urinary Bladder Metastatic to the Orbit. *Ophthalmic Plast Reconstr Surg*. 2003;19(2):165-167. doi:10.1097/01.IOP.0000056026.84209.1B
98. Holland D, Maune S, Kovács G, Behrendt S. Metastatic tumors of the orbit: A retrospective study. *Orbit*. 2003;22(1):15-24. doi:10.1076/orbi.22.1.15.14007
99. Lekse JM, Zhang J, Mawn LA. Metastatic gastroesophageal junction adenocarcinoma to the extraocular muscles. *Ophthalmology*. 2003;110(2):318-321. doi:10.1016/S0161-6420(02)01559-2
100. Saleh TA, Hakin KN, Davidson MJ. Metastasis of acinic cell carcinoma of the parotid gland to the contralateral orbit. *Arch Ophthalmol (Chicago, Ill 1960)*. 2003;121(12):1783-1786. doi:10.1001/archopht.121.12.1783
101. TAKEMOTO Y, NISHIDA N, KOJIRO S, JIMI A, KOJIRO M. Metastatic Carcinoid Tumor in the Orbit. *Kurume Med J*. 2003;50(3/4):165-167. doi:10.2739/kurumemedj.50.165
102. Tehrani A-H, Heegaard S, Prause J, Fledelius H, Daugaard S. Liposarcoma Metastatic to the Orbit. *Eur J Ophthalmol*. 2003;13(1):108-112. doi:10.1177/112067210301300121
103. Zografos L, Ducrey N, Beati D, et al. Metastatic melanoma in the eye and orbit. *Ophthalmology*. 2003;110(11):2245-2256. doi:10.1016/j.ophtha.2003.05.004
104. Chua WCT, Martin PA, Kourt G. Clinical Case Notes. Orbital metastasis from transitional cell carcinoma of the bladder. *Clin Exp Ophthalmol*. 2004;32(4):447-449. doi:10.1111/j.1442-9071.2004.00856.x
105. Glazer-Hockstein C, Syed NA, Warhol M, Gausas RE. Malignant Solitary Fibrous Tumor Metastatic to the Orbit. *Ophthalmic Plast Reconstr Surg*. 2004;20(6):471-473. doi:10.1097/01.IOP.0000144786.56696.98
106. Lell M, Schulz-Wendtland R, Hafner A, Magener A, Bautz WA, Tomandl BF. Bilateral orbital tumour as the presentation of mammographically occult breast cancer. *Neuroradiology*. 2004;46(8):682-685. doi:10.1007/s00234-003-1106-x
107. Van Der Zee J, Koper PCM, Jansen RFM, De Winter KAJ, Van Rhoon GC. Re-irradiation and hyperthermia for recurrent breast cancer in the orbital region: a case report. *Int J Hyperth*. 2004;20(1):1-6. doi:10.1080/02656730310001609344
108. Zdinak LA, Nik NA, Hidayat AA, Hargett NA. Renal Medullary Carcinoma Metastatic to the Orbit: A Clinicopathologic Report. *Ophthalmic Plast Reconstr Surg*. 2004;20(4):322-325. doi:10.1097/01.IOP.0000129530.75840.49
109. BOROTA OC, KLOSTER R, LINDAL S. Carcinoid tumour metastatic to the orbit with infiltration to the extraocular orbital muscle. Case report. *APMIS*. 2005;113(2):135-139. doi:10.1111/j.1600-0463.2005.apm1130207.x
110. Challagundla S, Gokden M, Viswamitra S, Kohli M. Orbital Metastasis from Prostate Cancer: An Atypical Case of Neuroendocrine Dedifferentiation During Progression from Hormone-Sensitive to Refractory Stage. *Clin Prostate Cancer*. 2005;4(2):134-137. doi:10.3816/CGC.2005.n.023
111. Fabi A, Salesi N, Vidiri A, Mirri A, Ferraresi V, Cognetti F. Retroperitoneal liposarcoma with metastasis to both orbits: an unusual metastatic site. *Anticancer Res*. 2005;25(6C):4769-4771. http://www.ncbi.nlm.nih.gov/pubmed/16334175
112. Gupta R, Honavar SG, Vemuganti GK. Orbital Metastasis from Hepatocellular Carcinoma. *Surv Ophthalmol*. 2005;50(5):485-489. doi:10.1016/j.survophthal.2005.06.014
113. Hart RH, Luthert PJ, Rose GE. Renal Cell Carcinoma Metastasis Masquerading as Recurrent Orbital Haematoma. *Orbit*. 2005;24(4):281-284. doi:10.1080/01676830500263794
114. Konuk O, Pehlivanli Z, Yirmibesoglu E, Erkal HS, Erekul S, Unal M. Compressive Optic Neuropathy Due to Orbital Metastasis of a Sacral Chordoma: Case Report. *Ophthalmic Plast Reconstr Surg*. 2005;21(3):245-247. doi:10.1097/01.IOP.0000159175.89287.1F
115. Mohadjer Y, Holds JB. Orbital Metastasis as the Initial Finding of Breast Carcinoma: A Ten-Year Survival. *Ophthalmic Plast Reconstr Surg*. 2005;21(1):65-66. doi:10.1097/01.IOP.0000150350.35376.11
116. Puglisi F, Capuano P, Gentile A, et al. Retrobulbar Metastasis from Gallbladder Carcinoma after Laparoscopic Cholecystectomy. A Case Report. *Tumori J*. 2005;91(5):428-431. doi:10.1177/030089160509100510
117. Aralikatti DA, Nylander AGE. Orbital Metastasis from Squamous Cell Carcinoma of the Esophagus. *Eur J Ophthalmol*. 2006;16(3):458-460. doi:10.1177/112067210601600316
118. Lee KYC, Jap A, Cheah E, Looi A. Orbital metastatic tumour as initial manifestation of asymptomatic gastric adenocarcinoma. *Ann Acad Med Singapore*. 2006;35(10):719-722. http://www.ncbi.nlm.nih.gov/pubmed/17102896

119. Mehta JS, Abou-Rayyah Y, Rose GE. Orbital Carcinoid Metastases. *Ophthalmology*. 2006;113(3):466-472. doi:10.1016/j.ophtha.2005.10.051
120. Oida Y, Ohtani Y, Dowaki S, et al. Hepatocellular carcinoma metastatic to the orbit: a case report. *Tokai J Exp Clin Med*. 2006;31(1):7-10. http://www.ncbi.nlm.nih.gov/pubmed/21302214
121. Schick U, Lermen O, Hassler W. Management of Orbital Metastases. *Zentralbl Neurochir*. 2006;67(01):1-7. doi:10.1055/s-2005-836922
122. Sharma K, Verma A, Rathi B, Kumar R, Kanaujia V. Metastatic Tumor of Orbit Presenting as Pulsatile Proptosis. *Ann Ophthalmol*. 2006;38(1):69-72. doi:10.1385/AO:38:1:69
123. Singh RP, Tullis S, Hatton M, Rubin PAD. Orbital Metastasis From Ovarian Carcinoma in a Patient With BRCA-2 Mutation. *Ophthalmic Plast Reconstr Surg*. 2006;22(4):298-299. doi:10.1097/01.iop.0000222355.09670.d7
124. Solari HP, Ventura MP, Cheema DP, Odashiro AN, Burnier MN. Orbital metastasis from breast carcinoma presenting as neurotrophic keratitis. *Can J Ophthalmol*. 2006;41(1):93-96. doi:10.1016/S0008-4182(06)80075-X
125. Tumuluri K, Sharkawi E, Bindra M, Olver JM. Esophageal Adenocarcinoma Metastatic to the Orbit. *Ophthalmic Plast Reconstr Surg*. 2006;22(2):151-152. doi:10.1097/01.iop.0000200920.30405.c1
126. Yunker JJ, Vicinanzo MG, Braswell RA, Read RW, Goldin GF, Long JA. Unusual Presentation of Gastric Adenocarcinoma Metastatic to the Orbit. *Ophthalmic Plast Reconstr Surg*. 2006;22(6):490-491. doi:10.1097/01.iop.0000245484.65470.51
127. Char DH, Moretto JC, Barakos JA. Cystic Orbital Metastasis from Endometrial Carcinoma. *Orbit*. 2007;26(1):75-77. doi:10.1080/01676830600675400
128. Gerencer RZ, Patel U, Hunter C, Heffernan JT. The Role of Endoscopic Sinus Surgery in the Diagnosis and Treatment of Metastatic Orbital Carcinoid Tumors. *Ear, Nose Throat J*. 2007;86(3):157-161. doi:10.1177/014556130708600312
129. Isshiki S, Cho S, Matsuno D, Sato N, Furuya Y. A case of orbital metastasis from prostatic carcinoma as an initial symptom. *Hinyokika Kiyo*. 2007;53(3):193-195. http://www.ncbi.nlm.nih.gov/pubmed/17447492
130. Lin H-C, Chang C-H, Li W-M, et al. Orbital Metastasis from Urothelial Carcinoma of the Urinary Bladder. *Kaohsiung J Med Sci*. 2007;23(2):84-88. doi:10.1016/S1607-551X(09)70380-8
131. Mani N, Lowe D, Pope L, El-Daly H, Pfleiderer A. An unusual case of laryngeal spindle cell carcinoma metastasising to the orbit and heart. *J Laryngol Otol*. 2007;121(09):e19. doi:10.1017/S0022215107009498
132. Su GW, Hong SH. Leiomyosarcoma of the Uterus With Sphenoid Bone and Orbital Metastases. *Ophthalmic Plast Reconstr Surg*. 2007;23(5):428-430. doi:10.1097/IOP.0b013e3181469ba4
133. Talwar V, Vaid AK, Doval DC, Bhatia K, Jena A, Anand AK. Isolated intraorbital metastasis in breast carcinoma. *J Assoc Physicians India*. 2007;55(JUNE):451-452. http://www.ncbi.nlm.nih.gov/pubmed/17879503
134. Torres JJ, Medel R, Alonso T, et al. Orbital Metastases From Male Breast Cancer in Two Cases. *Ophthalmic Plast Reconstr Surg*. 2007;23(2):154-156. doi:10.1097/IOP.0b013e31803316cb
135. Uluocak N, Suha Parlaktas B, Ersay Deniz F, Erdemir F, Dogan Koseoglu R, Gedar MO. Orbital Metastasis of Prostate Cancer: A Case Report. *Kaohsiung J Med Sci*. 2007;23(4):199-202. doi:10.1016/S1607-551X(09)70398-5
136. Alsuhaibani AH, Carter KD, Nerad JA, Lee AG. Prostate Carcinoma Metastasis to Extraocular Muscles. *Ophthalmic Plast Reconstr Surg*. 2008;24(3):233-235. doi:10.1097/IOP.0b013e3181706d08
137. Hatton MP, Green L, Boulos PR, Rubin PAD. Rhabdomyosarcoma Metastases to All Extraocular Muscles. *Ophthalmic Plast Reconstr Surg*. 2008;24(4):336-338. doi:10.1097/IOP.0b013e31817894c3
138. Henning M, Hu Q, Siegelmann-Danieli N. Orbital metastasis as the presenting symptom of extensive stage small cell lung cancer. *Eur J Intern Med*. 2008;19(1):65-66. doi:10.1016/j.ejim.2007.03.011
139. Hirunwiwatkul P, Tirakunwichcha S, Meesuaypong P, Shuangshoti S. Orbital Metastasis of Hepatocellular Carcinoma. *J Neuro-Ophthalmology*. 2008;28(1):47-50. doi:10.1097/WNO.0b013e31816754e7
140. Kiratli H, Yilmaz PT, Yildiz ZI. Metastatic Atypical Carcinoid Tumor of the Inferior Rectus Muscle. *Ophthalmic Plast Reconstr Surg*. 2008;24(6):482-484. doi:10.1097/IOP.0b013e31818b6d02
141. Kuo S-C, Hsiao S-C, Chiou C-C, Chen F-F, Huang K-C. Metastatic carcinoma of the breast: A case with the unusual presentation of unilateral periorbital edema. *Jpn J Ophthalmol*. 2008;52(4):305-307. doi:10.1007/s10384-008-0562-6
142. Milman T, Pliner L, Langer PD. Breast Carcinoma Metastatic to the Orbit: An Unusually Late Presentation. *Ophthalmic Plast Reconstr Surg*. 2008;24(6):480-482. doi:10.1097/IOP.0b013e31818b6adc
143. Mudiyanselage SY, Prabhakaran VC, Davis GJ, Selva D. Metastatic Renal Cell Carcinoma Presenting as a Circumscribed Orbital Mass. *Eur J Ophthalmol*. 2008;18(3):483-485. doi:10.1177/112067210801800332
144. Peng K-L, Kao S-C, Yang C-F, Kau H-C, Tsai C-C, Hsu W-M. Metastatic prostatic adenocarcinoma to the orbit diagnosed by prostate-specific antigen staining. *Eye (Lond)*. 2008;22(2):320-322. doi:10.1038/sj.eye.6703065
145. Pitts J, Chang C-H, Mavrikakis I, Shaikh A, Rootman J. Hepatocellular carcinoma presenting as orbital bone metastasis. *Ophthal Plast Reconstr Surg*. 2008;24(6):477-479. doi:10.1097/IOP.0b013e31818c9558
146. Polito E, Pichierri P, Occhini R, Loffredo A, Moramarco A, Balestrazzi A. Orbital Metastasis Associated with Primary Breast Carcinoma in a Man Detected during Peribulbar Anesthesia for Cataract Surgery. *Eur J Ophthalmol*. 2008;18(6):1031-1033. doi:10.1177/112067210801800633
147. Preechawai P, Amrith S, Yip CC, Goh KY. Orbital Metastasis of Renal Cell Carcinoma Masquerading as Cysticercosis. *Orbit*. 2008;27(5):370-373. doi:10.1080/01676830802316688
148. Rocha Filho FD, Lima GG, Ferreira FV de A, Lima MG, Hissa MN. Orbital metastasis as primary clinical manifestation of thyroid carcinoma: case report and literature review. *Arq Bras Endocrinol Metabol*. 2008;52(9):1497-1500. doi:10.1590/S0004-27302008000900014
149. Seiff BD, Seiff SR. Orbital Metastasis From Medullary Thyroid Carcinoma. *Ophthalmic Plast Reconstr Surg*. 2008;24(6):484-485. doi:10.1097/IOP.0b013e31818b751d
150. Souayah N, Krivitskaya N, Lee H-J. Lateral Rectus Muscle Metastasis As the Initial Manifestation of Gastric Cancer. *J Neuro-Ophthalmology*. 2008;28(3):240-241. doi:10.1097/WNO.0b013e318177253a
151. Surace D, Piscioli I, Morelli L, Valduga F, Licci S. Orbital metastasis as the first sign of “Dormant” breast cancer dissemination 25 years after mastectomy. *Jpn J Ophthalmol*. 2008;52(5):423-425. doi:10.1007/s10384-008-0555-5
152. Gosslee JM, Misra RP, Langford MP, Vekovius B, Byrd WA, Flynn SB. Orbital metastasis of keratinizing squamous cell cervical carcinoma with giant cells. A case report. *Int Ophthalmol*. 2009;29(1):39-44. doi:10.1007/s10792-007-9162-6
153. Nirmala S, Janaki M, Malavika K, Rishi K. Invasive thymoma with intraorbital metastases. *J Cancer Res Ther*. 2009;5(1):41. doi:10.4103/0973-1482.48768
154. Slagle WS, Eckermann DR, Musick AN, Slagle AM. Adenocarcinoma metastasis causing discrete extraocular muscle enlargement. *Optom - J Am Optom Assoc*. 2009;80(7):367-374. doi:10.1016/j.optm.2008.12.010
155. Valenzuela AA, Archibald CW, Fleming B, et al. Orbital Metastasis: Clinical Features, Management and Outcome. *Orbit*. 2009;28(2-3):153-159. doi:10.1080/01676830902897470
156. Barahimi B, Patel A, Bilyk JR. Orbital Metastasis Mimicking Subperiosteal Abscess. *Orbit*. 2010;29(3):166-168. doi:10.3109/01676830903537120
157. Foo FY, Lee M, Looi A. Asymptomatic Pancreatic Adenocarcinoma Presenting as an Orbital Metastatic Tumor. *Orbit*. 2010;29(5):262-265. doi:10.3109/01676830.2010.485719
158. Francone E, Murelli F, Paroldi A, Margarino C, Friedman D. Orbital swelling as a first symptom in breast carcinoma diagnosis: a case report. *J Med Case Rep*. 2010;4(1):211. doi:10.1186/1752-1947-4-211
159. Hassane S, Fouad E, Said I, et al. Orbital Metastatic Angiosarcoma. *Korean J Ophthalmol*. 2010;24(6):364. doi:10.3341/kjo.2010.24.6.364
160. Matsuo T, Ichimura K, Tanaka T, Takenaka T, Nakayama T. Neuroendocrine Tumor (Carcinoid) Metastatic to Orbital Extraocular Muscle: Case Report and Literature Review. *Strabismus*. 2010;18(4):123-128. doi:10.3109/09273972.2010.525779
161. Ng E, Ilsen PF. Orbital Metastases. *Optom - J Am Optom Assoc*. 2010;81(12):647-657. doi:10.1016/j.optm.2010.07.026
162. Patel V, Castell FA, Akinwunmi J, Francis I, Chandrasekharan L, Malhotra R. Prostatic Adenocarcinoma Presenting with Metastatic Frontal Bone Involvement and Orbital Invasion. *Orbit*. 2010;29(4):213-215. doi:10.3109/01676831003664301
163. Eckardt AM, Rana M, Essig H, Gellrich N-C. Orbital metastases as first sign of metastatic spread in breast cancer: Case report and review of the literature. *Head Neck Oncol*. 2011;3(1):37. doi:10.1186/1758-3284-3-37
164. Galm T, Kulkarni A, Ahmad I. Laryngeal carcinoma metastasis to the orbit: case report. *J Laryngol Otol*. 2011;125(5):533-535. doi:10.1017/S0022215110002860
165. Guerriero S, Infante G, Giancipoli E, et al. Hepatocellular Carcinoma Metastasis to the Orbit in a Coinfected HIV+ HBV+ Patient Previously Treated with Orthotopic Liver Transplantation: A Case Report. *Case Rep Ophthalmol Med*. 2011;2011:1-4. doi:10.1155/2011/549270
166. Gupta S, Bhatt VR, Varma S. Unilateral orbital pain and eyelid swelling in a 46-year-old woman: orbital metastasis of occult invasive lobular carcinoma of breast masquerading orbital pseudotumour. *Case Reports*. 2011;2011(mar10 1):bcr1220103580-bcr1220103580. doi:10.1136/bcr.12.2010.3580
167. Janicijevic-Petrovic M, Sarenac T, Sreckovic S, Vulovic D, Janicijevic K. Orbital metastases from breast cancer: a case report. *Bosn J Basic Med Sci*. 2011;11(4):253. doi:10.17305/bjbms.2011.2561
168. Konoglou M, Zarogoulidis P, Porpodis K, et al. Exophthalmos as a First Manifestation of Small Cell Lung Cancer: A Long-Term Follow-Up. *Case Rep Ophthalmol*. 2011;2(3):360-366. doi:10.1159/000334784
169. Li L-F, Tse Y-H, Ho S-L, Yan K-W, Lui W-M. Duodenal GIST metastasized to skull and orbit managed by surgery – A case report. *Asian J Surg*. 2011;34(4):181-184. doi:10.1016/j.asjsur.2011.11.001
170. Murthy R, Hegde S, Gupta A, Honavar S. Bilateral multiple extraocular muscle metastasis from breast carcinoma. *Indian J Ophthalmol*. 2011;59(5):381. doi:10.4103/0301-4738.83616
171. Mustapha SK, Madachi DA. Orbital metastasis of hepatocellular carcinoma: a case report. *West Afr J Med*. 2000;30(4):305-307. doi:10.7887/jcns.9.707
172. Rosado P, de Vicente JC, Vivanco B, de Villalaín L, Santamarta T. Clinical and Immunohistochemical Analysis of Orbital Metastasis From Prostate Carcinoma. *J Craniofac Surg*. 2011;22(6):2141-2143. doi:10.1097/SCS.0b013e3182323d29
173. Zarogoulidis P, Terzi E, Kouliatsis G, et al. Orbital Metastases as the First Manifestation of Lung Adenocarcinoma. *Case Rep Ophthalmol*. 2011;2(1):34-38. doi:10.1159/000323945
174. Charles NC, Ng DD, Zoumalan CI. Signet Cell Adenocarcinoma of the Rectum Metastatic to the Orbit. *Ophthalmic Plast Reconstr Surg*. 2012;28(1):e1-e2. doi:10.1097/IOP.0b013e31820d18c0
175. Chen J, Wei R, Ma X. Orbital metastasis of retroperitoneal leiomyosarcoma. *Med Oncol*. 2012;29(1):392-395. doi:10.1007/s12032-010-9809-4
176. Dmuchowska DA, Krasnicki P, Obuchowska I, Kochanowicz J, Syta-Krzyżanowska A, Mariak Z. Ophthalmic manifestation of skull base metastasis from breast cancer. *Med Sci Monit*. 2012;18(11):CS105-CS108. doi:10.12659/MSM.883532

177. Jiang H, Wang Z, Xian J, Ai L. Bilateral Multiple Extraocular Muscle Metastasis from Hepatocellular Carcinoma. *Acta Radiol Short Reports*. 2012;1(1):1-3. doi:10.1258/arsr.2011.110002
178. Jung JW, Yoon SC, Han DH, Chi M. Metastatic Renal Cell Carcinoma to the Orbit and the Ethmoid Sinus. *J Craniofac Surg*. 2012;23(2):e136-e138. doi:10.1097/SCS.0b013e31824cdb31
179. Kim HJ, Wojno TH, Grossniklaus H. Atypical Bilateral Orbital Metastases of Lobular Breast Carcinoma. *Ophthalmic Plast Reconstr Surg*. 2012;28(6):e142-e143. doi:10.1097/IOP.0b013e318249d5c0
180. Pecen P, Pecen P, Ramey, Richard, Bhatti. Metastatic pancreatic carcinoma to the orbital apex presenting as a superior divisional third cranial nerve palsy. *Clin Ophthalmol*. 2012;6(1):1941. doi:10.2147/OPTH.S30208
181. SooHoo JR, Gonzalez MO, Siomos VJ, Durairaj VD. Urothelial Carcinoma With Orbital Metastasis. *Urology*. 2012;80(4):e45-e46. doi:10.1016/j.urology.2012.06.002
182. Tomizawa Y, Ocque R, Ohori NP. Orbital Metastasis as the Initial Presentation of Invasive Lobular Carcinoma of Breast. *Intern Med*. 2012;51(12):1635-1638. doi:10.2169/internalmedicine.51.7641
183. Wladis EJ, Farber MG, Nepo AG. Metastatic Synovial Sarcoma to the Orbit. *Ophthalmic Plast Reconstr Surg*. 2012;28(6):e131-e132. doi:10.1097/IOP.0b013e3182467e11
184. Woo D, Leong J, Waring D, Sharma A, Martin P. Orbital Gastrointestinal Stromal Tumor Metastasis. *Orbit*. 2012;31(2):129-131. doi:10.3109/01676830.2011.638100
185. Dobson R, Vinjamuri S, Hsuan J, et al. Treatment of Orbital Metastases From a Primary Midgut Neuroendocrine Tumor With Peptide-Receptor Radiolabeled Therapy Using 177 Lutetium-DOTATATE. *J Clin Oncol*. 2013;31(17):e272-e275. doi:10.1200/JCO.2012.45.8612
186. Fini G, Grippaudo FR, Fenicia V, et al. Orbital Metastases in a Female Patient with Breast Cancer. *Eur J Inflamm*. 2013;11(2):547-552. doi:10.1177/1721727X1301100226
187. FUJIMOTO K, KURODA J, MAKINO K, HASEGAWA Y, KURATSU J. Skull Metastasis From Intrahepatic Cholangiocarcinoma: Report of 3 Cases and Review of the Literature. *Neurol Med Chir (Tokyo)*. 2013;53(10):717-721. doi:10.2176/nmc.cr2012-0237
188. Patel MM, Jakobiec FA, Zakka FR, et al. Intraorbital Metastasis From Solitary Fibrous Tumor. *Ophthalmic Plast Reconstr Surg*. 2013;29(3):e76-e79. doi:10.1097/IOP.0b013e318272f311
189. Patel MM, Lefebvre DR, Lee NG, Brachtel E, Rizzo J, Freitag SK. Gaze-Evoked Amaurosis From Orbital Breast Carcinoma Metastasis. *Ophthalmic Plast Reconstr Surg*. 2013;29(4):e98-e101. doi:10.1097/IOP.0b013e31827defc7
190. Coloma-González I, Ceriotto A, Amezquita-García E, Flores-Preciado J, Salcedo-Casillas G. Orbital pulsatile metastasis as initial presenting sign of metastatic clear cell renal carcinoma. *Arch la Soc Española Oftalmol (English Ed*. 2014;89(12):500-503. doi:10.1016/j.oftale.2014.11.008
191. Eldesouky MA, Elbakary MA, Shalaby OE, Shareef MM. Orbital Metastasis From Hepatocellular Carcinoma. *Ophthalmic Plast Reconstr Surg*. 2014;30(4):e78-e82. doi:10.1097/IOP.0b013e31829f3a57
192. Greene DP, Shield DR, Shields CL, et al. Cutaneous Melanoma Metastatic to the Orbit. *Ophthalmic Plast Reconstr Surg*. 2014;30(3):233-237. doi:10.1097/IOP.0000000000000075
193. Kapur. Extraconal Orbital Soft Tissue Metastasis Secondary to Prostate Cancer: An Unusual Presentation. *World J Oncol*. 2014;5(3):139-143. doi:10.14740/wjon807w
194. Ludmir EB, McCall SJ, Czito BG, Palta M. Radiosensitive orbital metastasis as presentation of occult colonic adenocarcinoma. *Case Reports*. 2014;2014(sep19 2):bcr2014206407-bcr2014206407. doi:10.1136/bcr-2014-206407
195. Saffra N, Rakhamimov A, Wrzolek MA, Solomon WB, Cooper J, Borgen P. Orbital Metastasis as the Initial Presentation in Bilateral Lobular Invasive Carcinoma of the Breast. *Ophthalmic Plast Reconstr Surg*. 2014;30(2):e30-e32. doi:10.1097/IOP.0b013e3182916537
196. Gupta D, Chappell M, Tailor TD, et al. Orbital Metastasis of Undifferentiated/Anaplastic Thyroid Carcinoma. *Ophthalmic Plast Reconstr Surg*. 2015;31(5):e120-e123. doi:10.1097/IOP.0000000000000145
197. Magliozzi P, Strianese D, Bonavolontà P, et al. Orbital metastases in Italy. *Int J Ophthalmol*. 2015;8(5):1018-1023. doi:10.3980/j.issn.2222-3959.2015.05.30
198. Magrath GN, Proctor CM, Reardon WA, Patel KG, Lentsch EJ, Eiseman AS. Esophageal Adenocarcinoma and Urothelial Carcinoma Orbital Metastases Masquerading as Infection. *Orbit*. 2015;34(1):51-55. doi:10.3109/01676830.2014.963879
199. Pagsisihan DA, Aguilar AHI, Maningat MPDD. Orbital metastasis as initial manifestation of a widespread papillary thyroid microcarcinoma. *Case Reports*. 2015;2015(mar27 1):bcr2014208870-bcr2014208870. doi:10.1136/bcr-2014-208870
200. Pearlman M, Kwong WT. A long and distant journey: a case of rectal cancer with metastasis to the orbit. *Ann Gastroenterol*. 2015;28(1):151-152. http://www.ncbi.nlm.nih.gov/pubmed/25608827
201. Rajabi MT, Saeedi-Anari G, Ramezani F, Tabatabaie S-Z, Rajabi MB, Asadi Amoli F. Orbital metastatic osteosarcoma. *Arch Iran Med*. 2015;18(2):123-126. doi:015182/AIM.0012
202. Arthur A. Orbital Metastasis of Cervical Carcinoma – Case Report and Review of Literature. *J Clin DIAGNOSTIC Res*. 2016;10(1):ND01-ND02. doi:10.7860/JCDR/2016/14400.7085
203. Foster CR, Cohen AJ. Signet ring adenocarcinoma of the colon presenting as an orbital metastasis. *Eur J Plast Surg*. 2016;39(6):459-462. doi:10.1007/s00238-016-1222-6
204. MacLean KD, Cole SC, Ford JR, Owen L, Mamalis N, Patel BCK. Thymic Origin Neuroendocrine Carcinoma Metastasizing to the Orbit in an Otherwise Asymptomatic Patient. *Ophthalmic Plast Reconstr Surg*. 2016;32(1):e21-e23. doi:10.1097/IOP.0000000000000375
205. Pokharel S, Kabbach G, Richter SJ, Chiu L. Metastatic Esophageal Cancer Presenting as an Orbital Mass. *ACG Case Reports J*. 2016;3(1):e154. doi:10.14309/crj.2016.127
206. Tabai M, Hazboun IM, Sakuma ETI, Sampaio MH, Sakano E. Orbital Metastasis of Breast Cancer Mimicking Invasive Fungal Rhinosinusitis. *Case Rep Otolaryngol*. 2016;2016:1-4. doi:10.1155/2016/2913241
207. Tun K, Bulut T. Triple orbital metastases from prostate cancer. *Neurol Neurochir Pol*. 2016;50(5):392-394. doi:10.1016/j.pjnns.2016.06.007
208. AufderHeide AC, Bernard BJ, Mollman RA, et al. Metastatic prostate carcinoma to the orbit as the first presentation of disease. *Orbit*. 2017;36(4):234-236. doi:10.1080/01676830.2017.1310257
209. Geske MJ, Bloomer MM, Kersten RC, Vagefi MR. Diagnostic Approaches to Metastatic Hepatocellular Carcinoma of the Orbit. *Ophthalmic Plast Reconstr Surg*. 2017;33(3S):S129-S131. doi:10.1097/IOP.0000000000000641
210. Huang Y-Y, Chang A, Chou Y-Y, Hsu W-C. Metastatic neuroendocrine tumor with initial presentation of orbital apex syndrome. *Interdiscip Neurosurg*. 2017;7:9-11. doi:10.1016/j.inat.2016.10.009
211. Mărginean A, Gheorghe C, Margan MM, et al. Intraorbital tumor as the first and sole manifestation of breast cancer. *Gineco.eu*. 2017;13(3):109-110. doi:10.18643/gieu.2017.109
212. Nabeel M, Farooqi R, Mohebtash M, et al. Rectal Cancer in the Eye: A Case Report of Orbital Metastasis. *Cureus*. 2017;9(8). doi:10.7759/cureus.1589
213. Nash S, Bartels H, Pemberton J. Metastatic cervical adenocarcinoma to the orbital subperiosteal space. *Can J Ophthalmol*. 2017;52(2):e60-e62. doi:10.1016/j.jcjo.2016.10.008
214. Rakul Nambiar K, Ajith PS, Arjunan A. Unilateral proptosis as the initial manifestation of malignancy. *J Egypt Natl Canc Inst*. 2017;29(3):159-161. doi:10.1016/j.jnci.2017.05.004
215. Rasool N. Orbital leiomyosarcoma metastasis presenting prior to diagnosis of the primary tumor. *Digit J Ophthalmol*. 2017;23(4):22. doi:10.5693/djo.02.2017.02.004
216. Wang Y, Mettu P, Maltry A, Harrison A, Mokhtarzadeh A. Metastatic Breast Carcinoma to the Superior Oblique in a Male. *Ophthalmol Ther*. 2017;6(2):355-359. doi:10.1007/s40123-017-0093-7
217. Coutinho I, Marques M, Almeida R, Custódio S, Simões Silva T, Águas F. Extraocular Muscles Involvement as the Initial Presentation in Metastatic Breast Cancer. *J Breast Cancer*. 2018;21(3):339. doi:10.4048/jbc.2018.21.e46
218. Das S, Pineda G, Goff L, Sobel R, Berlin J, Fisher G. The eye of the beholder: orbital metastases from midgut neuroendocrine tumors, a two institution experience. *Cancer Imaging*. 2018;18(1):47. doi:10.1186/s40644-018-0181-5
219. Dutton JJ, Proia AD. Seminoma Metastatic to the Orbit. *Ophthalmic Plast Reconstr Surg*. 2018;34(4):309-312. doi:10.1097/IOP.0000000000001031
220. Gulmez Sevim D, Unlu M, Kontas O, Selcuklu A. Unilateral Proptosis as the Presenting Feature of Papillary Thyroid Carcinoma. *Erciyes Tıp Dergisi/Erciyes Med J*. 2018;40(1):50-53. doi:10.5152/etd.2018.17111
221. Nifosí G, Zuccarello M. Unilateral localized extraocular muscle metastasis by lobular breast carcinoma. *BMJ Case Rep*. 2018;2018:bcr-2018-224726. doi:10.1136/bcr-2018-224726
222. Pinto Proença R, Fernandes J, Burnier MN, Proença R. Orbital metastasis from an occult breast carcinoma (T0, N1, M1). *BMJ Case Rep*. 2018;2018(April):bcr-2017-223542. doi:10.1136/bcr-2017-223542
223. Ramsey JK, Chen JL, Schoenfield L, Cho RI. Undifferentiated Pleomorphic Sarcoma Metastatic to the Orbit. *Ophthalmic Plast Reconstr Surg*. 2018;34(6):e193-e195. doi:10.1097/IOP.0000000000001240
224. Roelofs KA, Medlicott S, Henning J-W, Weis E. Gastrointestinal Stromal Tumor Metastasis to the Orbit. *Ophthalmic Plast Reconstr Surg*. 2018;34(4):e131-e133. doi:10.1097/IOP.0000000000001144
225. Rong AJ, Choi CJ, Ghassibi MP, Dubovy SR, Johnson TE. Orbital metastatic small cell carcinoma of the pancreas with optic nerve compression. *Can J Ophthalmol*. 2018;53(1):e22-e24. doi:10.1016/j.jcjo.2017.06.017
226. Shaikh R, Tariq K, Sharma S, Tang S-C. Metastatic Breast Cancer Presenting As Orbital Mass: A Case Report With Literature Review. *J Glob Oncol*. 2018;2018(4):1-4. doi:10.1200/JGO.2017.009282
227. Chumdermpadetsuk R, Tooley AA, Godfrey KJ, Krawitz B, Feldstein N, Kazim M. Renal Medullary Carcinoma With Metastasis to the Temporal Fossa and Orbit. *Ophthalmic Plast Reconstr Surg*. 2019;35(6):e149-e151. doi:10.1097/IOP.0000000000001478
228. Crisostomo S, Cardigos J, Fernandes DH, et al. Bilateral metastases to the extraocular muscles from small cell lung carcinoma. *Arq Bras Oftalmol*. 2019;82(5):422-424. doi:10.5935/0004-2749.20190081
229. Cruzado-Sánchez D, Sánchez-Ortiz J, Peralta CI, Tellez WA, Maquera-Torres G, Serpa-Frías S. Orbital metastasis diagnosed by ultrasound-guided fine-needle aspiration biopsy: case report of unknown primary site. *Arch la Soc Española Oftalmol (English Ed*. 2019;94(11):566-570. doi:10.1016/j.oftale.2019.07.004
230. Espinosa-Barberi G, Alba Linero C, Galván González FJ, Álvarez González E, Rey López A, Medina Rivero F. Study of aggressive carcinomas through orbital metastasis. *Arch la Soc Española Oftalmol (English Ed*. 2019;94(5):252-256. doi:10.1016/j.oftale.2018.10.013
231. Framarino-dei-Malatesta M, Chiarito A, Bianciardi F, et al. Metastases to extraocular muscles from breast cancer: case report and up-to-date review of the literature. *BMC Cancer*. 2019;19(1):36. doi:10.1186/s12885-018-5253-1
232. Hernández Pardines F, Serra Verdú MC, Bernal Vidal A, Mayol Belda JM, Mengual Verdú E. Lateral rectus muscle biopsy as diagnosis of unknown metastatic breast cancer. *Arch la Soc Española Oftalmol (English Ed*. 2019;94(4):192-195. doi:10.1016/j.oftale.2018.09.008
233. Kagusa H, Mizobuchi Y, Nakajima K, Fujihara T, Bando Y, Takagi Y. Metastatic tumor to the orbital cavity from a primary carcinoma of the uterine cervix : a case report. *J Med Investig*. 2019;66(3.4):355-357. doi:10.2152/jmi.66.355

234. Kızıloğlu ÖY, Türköz FP, Gedar ÖMT, Mestanoğlu M, Yapıcıer Ö. Breast carcinoma metastasis to the medial rectus muscle: Case report. *Turkish J Ophthalmol*. 2019;49(3):168-170. doi:10.4274/tjo.galenos.2018.39018
235. Kluska A, Papis-Ubych A, Fijuth J, Loga K, Spych M, Gottwald L. Intraorbital extraocular metastasis of breast cancer 11 years after mastectomy – case report and review of the literature. *J Obstet Gynaecol (Lahore)*. 2019;39(1):126-128. doi:10.1080/01443615.2018.1455078
236. Kozubowska K, Skorek A, Pęksa R. Neuroendocrine tumour metastasis to the orbit. *Endokrynol Pol*. 2019;70(5):455-456. doi:10.5603/EP.a2019.0024
237. Rider AJ, Walsh A, Sollenberger EL, et al. Orbital Pheochromocytoma Metastasis in 2 Patients With Known Pheochromocytoma. *Ophthalmic Plast Reconstr Surg*. 2019;35(6):e131-e134. doi:10.1097/IOP.0000000000001460
238. Sochat MM, Piao J, Poddar N. Hepatocellular Carcinoma with Orbital Metastasis: a Unique Multidisciplinary Case Report. *J Gastrointest Cancer*. 2019;50(4):978-982. doi:10.1007/s12029-018-0159-3
239. Tao Y, Yagi K, Nishimura H, Hara K, Matsubara S, Uno M. Orbitotemporal Bone Cyst of Metastatic Breast Cancer: Case Report and Literature Review. *World Neurosurg*. 2019;130:267-270. doi:10.1016/j.wneu.2019.07.076
240. Yadav D, Singh M, Gupta K, Kaur M, Gupta P. A report of orbital metastasis from a urinary bladder adenocarcinoma. *Indian J Ophthalmol*. 2019;67(8):1377. doi:10.4103/ijo.IJO_1699_18
241. Yu Y, Ji X, Li W, Wu C. Orbital metastasis from a gastrointestinal stromal tumor: A case report. *Am J Ophthalmol Case Reports*. 2019;16(January):100528. doi:10.1016/j.ajoc.2019.100528
242. Bacorn C, Lin LK. Rare Signet Ring Cell Adenocarcinoma of the Colon Metastasis to the Orbit. *Case Rep Ophthalmol Med*. 2020;2020(Figure 1):1-3. doi:10.1155/2020/2940579
243. Blohmer M, Zhu L, Atkinson JM, et al. Patient treatment and outcome after breast cancer orbital and periorbital metastases: a comprehensive case series including analysis of lobular versus ductal tumor histology. *Breast Cancer Res*. 2020;22(1):70. doi:10.1186/s13058-020-01309-3
244. El Bakraoui K, El Morabit B. Orbital Metastasis from Triple-Negative Breast Cancer: Case Report and Literature Review. *Case Rep Oncol*. 2020;13(2):1042-1046. doi:10.1159/000509348
245. Halliday LA, Curragh D, Sia PI, Selva D. Pseudocystic appearance of an orbital carcinoid metastasis. *Orbit*. 2020;39(1):41-44. doi:10.1080/01676830.2019.1576739
246. Hoenck H, Conradie W, Conradie M, Coetzee A. Midgut neuroendocrine tumour presenting as orbital metastases. *J Endocrinol Metab Diabetes South Africa*. 2020;25(2):24-27. doi:10.1080/16089677.2020.1722402
247. Long CP, Lu T, Ediriwickrema LS, et al. Colorectal carcinoma presenting in the orbit: mass effect from an uncommon cause. *Orbit*. 2021;40(4):338-341. doi:10.1080/01676830.2020.1787466
248. Madabhavi I, KS S, Dharmarajan Lethika R, et al. Intraconal Metastasis Leading to Diagnosis of Hepatocellular Carcinoma. *Middle East J Dig Dis*. 2019;12(1):48-51. doi:10.15171/mejdd.2020.164
249. Mahuvakar AD, Nair D, Pai PS. Transnasal Endoscopic Resection of the Intraconal Metastases From Renal Cell Carcinoma: a Case Report and Review of Literature. *Indian J Surg Oncol*. 2020;11(S2):318-322. doi:10.1007/s13193-020-01237-w
250. Marotta DA, Jabaay MJ, Zadourian A, Kesserwani H. Bilateral Orbital Metastases Masquerading as Ocular Myasthenia Gravis: A Case Report and Review of the Literature. *Cureus*. 2020;12(7). doi:10.7759/cureus.9105
251. Mian I, Padiernos E, Hassan R, Ghafoor A. Orbital metastases from malignant mesothelioma. *Lancet Oncol*. 2020;21(2):e117. doi:10.1016/S1470-2045(19)30822-8
252. Montejano-Milner R, López-Gaona A, Fernández-Pérez P, Sánchez-Orgaz M, Romero-Martín R, Arbizu-Duralde A. Orbital metastasis: Clinical presentation and survival in a series of 11 cases. *Arch la Soc Española Oftalmol (English Ed*. 2020;(x x). doi:10.1016/j.oftale.2020.07.014
253. Narayanan N, Padwal U, Gopinathan I, Pathak R, Nair A. Malignant melanoma of the rectum presenting as orbital metastasis. *Indian J Ophthalmol*. 2020;68(11):2620. doi:10.4103/ijo.IJO_847_20
254. Pastore MR, D'Aloisio R, Cirigliano G, De Giacinto C, Tognetto D. Orbital metastasis as presenting symptom from a prostatic adenocarcinoma. *Eur J Ophthalmol*. 2020;30(1):NP29-NP32. doi:10.1177/1120672119832182
255. Protopapa M-N, Lagadinou M, Papagiannis T, Gogos CA, Solomou EE. Hepatocellular Carcinoma: An Uncommon Metastasis in the Orbit. *Case Rep Oncol Med*. 2020;2020:1-3. doi:10.1155/2020/7526042
256. Sindoni A, Fama' F, Vinciguerra P, et al. Orbital metastases from breast cancer: A single institution case series. *J Surg Oncol*. 2020;122(2):170-175. doi:10.1002/jso.25927
257. Tsuruta Y, Maeda Y, Kitaguchi Y, et al. A Case of Endonasal Endoscopic Surgery for Intraorbital Metastasis of Gastric Ring Cell Carcinoma. *Ear, Nose Throat J*. Published online July 22, 2020:014556132094337. doi:10.1177/0145561320943372
258. Wada K, Tsuda T, Hanada Y, Maeda Y, Mori K, Nishimura H. A Rare Case of Prostate Carcinoma Metastasis in the Orbital Apex. *Ear, Nose Throat J*. Published online November 20, 2020:014556132097378. doi:10.1177/0145561320973783
259. El-Khazen Dupuis J, Marchand M, Javidi S, Nguyen TQT. Enophthalmos as the Initial Systemic Finding of Undiagnosed Metastatic Breast Carcinoma. *Int Med Case Rep J*. 2021;Volume 14:25-31. doi:10.2147/IMCRJ.S282113
260. Mendia A, Shah CP, Grajo JR, et al. Orbital metastasis of squamous cell cervical cancer: A case report and review of literature. *Gynecol Oncol Reports*. 2021;35(December 2020):100689. doi:10.1016/j.gore.2020.100689
261. Oprean CM, Badau LM, Segarceanu NA, et al. Unilateral Orbital Metastasis as the Unique Symptom in the Onset of Breast Cancer in a Postmenopausal Woman: Case Report and Review of the Literature. *Diagnostics*. 2021;11(4):725. doi:10.3390/diagnostics11040725
262. Razem B, Slimani F. An early orbital metastasis from breast cancer: A case report. *Int J Surg Case Rep*. 2021;78:300-302. doi:10.1016/j.ijscr.2020.12.049
