# Supplementary material for: Orbital Metastases: A Systematic Review of Clinical Characteristics, Management Strategies, and Treatment Outcomes
Source: Cancers (Basel). 2021 Dec 24;14(1):94. doi: 10.3390/cancers14010094 (PMC8750198; doi:10.3390/cancers14010094)
Supplement: Supplementary file 1 [file cancers-14-00094-s001.zip › Supplementary/Table S2 - Risk of bias.pdf]

Table S2. Risk of bias assessments for all included studies.

**Joanna Briggs Institute Checklist for Case Reports – Criteria**
1. Were patient's demographic characteristics clearly described?
2. Was the patient's history clearly described and presented as a timeline?
3. Was the current clinical condition of the patient on presentation clearly described?
4. Were diagnostic tests or assessment methods and the results clearly described?
5. Was the intervention(s) or treatment procedure(s) clearly described?
6. Was the post-intervention clinical condition clearly described?
7. Were adverse events (harms) or unanticipated events identified and described?
8. Does the case report provide takeaway lessons?
**Responses Options**: Yes, No, Unclear, Not Applicable (NA)
**Quality Rating**: Poor 0 – 2; Fair 3 – 5; Good 6 – 8

**Study (Case Reports)**	**1**	**2**	**3**	**4**	**5**	**6**	**7**	**8**	**Rating**
Arnott et al. – 1965[1]	Yes	Yes	Yes	Yes	Yes	Yes	Yes	No	7 – Good
Chatterjee et al. – 1965[2]	Yes	Yes	Yes	Yes	Yes	Yes	Yes	No	7 – Good
Honrubia et al. – 1971[4]	Yes	Yes	Yes	Yes	Yes	No	Yes	Yes	7 – Good
Saxena et al. – 1975[7]	Yes	Yes	Yes	Yes	Yes	Yes	Yes	No	7 – Good
Usui et al. – 1975[8]	Yes	Yes	Yes	Yes	Yes	Yes	Yes	No	7 – Good
Balestrazzi et al. – 1976[9]	Yes	Yes	Yes	Yes	Yes	Yes	No	Yes	7 – Good
Howard et al. – 1978[10]	Yes	Yes	Yes	Yes	Yes	Yes	Yes	Yes	8 – Good
Stewart et al. – 1978[11]	Yes	Yes	Yes	Yes	Yes	No	Yes	Yes	7 – Good
Bullock et al. – 1980[12]	Yes	Yes	Yes	Yes	Yes	Yes	Yes	Yes	8 – Good
Lubin et al. – 1980[13]	Yes	Yes	Yes	Yes	Yes	Yes	No	Yes	7 – Good
Rush et al. – 1980[14]	Yes	Yes	Yes	Yes	Yes	Yes	Yes	Yes	8 – Good
Sobol et al. – 1980[15]	Yes	Yes	Yes	Yes	Yes	No	Yes	Yes	7 – Good
Rush et al. – 1981[16]	Yes	Yes	Yes	Yes	Yes	No	No	Yes	6 – Good
Winkler et al. – 1981[17]	Yes	Yes	Yes	Yes	Yes	Yes	No	Yes	7 – Good
Wolter et al. – 1981[18]	Yes	Yes	Yes	Yes	Yes	Yes	No	Yes	7 – Good
Zubler et al. – 1981[19]	Yes	Yes	Yes	Yes	Yes	Yes	Yes	Yes	8 – Good
Carriere et al. – 1982[20]	Yes	Yes	Yes	Yes	Yes	No	No	Yes	6 – Good
Weitberg et al. – 1983[23]	Yes	Yes	Yes	Yes	Yes	Yes	Yes	Yes	8 – Good
Reifler et al. – 1984[25]	Yes	Yes	Yes	Yes	Yes	Yes	Yes	Yes	8 – Good
Dunaway et al. – 1985[26]	Yes	Yes	Yes	Yes	Yes	Yes	No	Yes	7 – Good
Denby et al. – 1986[28]	Yes	Yes	Yes	Yes	Yes	Yes	Yes	Yes	8 – Good
Schlaen et al. – 1986[29]	Yes	Yes	Yes	Yes	Yes	Yes	No	Yes	7 – Good
Hornblass et al. – 1987[30]	Yes	Yes	Yes	Yes	Yes	No	Yes	Yes	7 – Good
Kaltreider et al. – 1987[31]	Yes	Yes	Yes	Yes	Yes	Yes	Yes	Yes	8 – Good
Noci et al. – 1987[32]	Yes	Yes	Yes	Yes	Yes	Yes	Yes	No	7 – Good

Oosterhuis et al. – 1987[33]	Yes	Yes	Yes	Yes	Yes	No	Yes	Yes	7 – Good
Otto et al. – 1987[34]	Yes	Yes	Yes	Yes	Yes	Yes	Yes	Yes	8 – Good
Shields et al. – 1987[35]	Yes	Yes	Yes	Yes	Yes	No	Yes	Yes	7 – Good
Malviya et al. – 1989[41]	Yes	Yes	Yes	Yes	Yes	Yes	Yes	Yes	8 – Good
Ruusuvaara et al. – 1989[42]	Yes	Yes	Yes	Yes	Yes	Yes	No	Yes	7 – Good
Spaide et al. – 1989[43]	Yes	No	Yes	Yes	Yes	Yes	Yes	Yes	7 – Good
Friedman et al. – 1990[45]	Yes	Yes	Yes	Yes	Yes	Yes	Yes	Yes	8 – Good
Wakisaka et al. – 1990[48]	Yes	Yes	Yes	Yes	Yes	Yes	No	Yes	7 – Good
Felip et al. – 1991[49]	Yes	Yes	Yes	Yes	Yes	No	Yes	Yes	7 – Good
Glazer et al. – 1991[50]	Yes	Yes	Yes	Yes	Yes	Yes	Yes	Yes	8 – Good
Van der Heijden et al. – 1991[51]	Yes	Yes	Yes	Yes	Yes	Yes	Yes	No	7 – Good
Zucker et al. – 1991[52]	Yes	Yes	Yes	Yes	Yes	Yes	Yes	Yes	8 – Good
Brackup et al. – 1992[53]	Yes	Yes	Yes	Yes	Yes	Yes	Yes	Yes	8 – Good
Di Leo et al. – 1992[54]	Yes	Yes	Yes	Yes	Yes	Yes	Yes	Yes	8 – Good
Shetty et al. – 1992[55]	Yes	Yes	Yes	Yes	Yes	Yes	Yes	Yes	8 – Good
Bersani et al. – 1994[58]	Yes	Yes	Yes	Yes	Yes	Yes	Yes	Yes	8 – Good
Ellis et al. – 1994[59]	Yes	Yes	Yes	Yes	Yes	Yes	Yes	Yes	8 – Good
Hogkulstone et al. – 1994[60]	Yes	Yes	Yes	Yes	Yes	Yes	Yes	Yes	8 – Good
Loo et al. – 1994[61]	Yes	Yes	Yes	Yes	Yes	Yes	No	Yes	7 – Good
Tranfa et al. – 1994[62]	Yes	Yes	Yes	Yes	Yes	No	Yes	Yes	7 – Good
Aburn et al. – 1995[63]	Yes	Yes	Yes	Yes	Yes	Yes	Yes	Yes	8 – Good
Airoldi et al. – 1995[64]	Yes	Yes	Yes	Yes	Yes	Yes	Yes	No	7 – Good
Day et al. – 1995[65]	Yes	Yes	Yes	Yes	Yes	Yes	Yes	Yes	8 – Good
Rhatigan et al. – 1995[67]	Yes	Yes	Yes	Yes	Yes	Yes	Yes	Yes	8 – Good
Thomas et al. – 1995[68]	Yes	Yes	Yes	Yes	Yes	Yes	Yes	Yes	8 – Good
Burnstine et al. – 1996[69]	Yes	Yes	Yes	Yes	Yes	Yes	Yes	No	7 – Good
El-Toukhy et al. – 1996[70]	Yes	Yes	Yes	Yes	Yes	Yes	Yes	Yes	8 – Good
Fezza et al. – 1997[72]	Yes	Yes	Yes	Yes	Yes	No	Yes	Yes	7 – Good
Hayashi et al. – 1997[73]	Yes	Yes	Yes	Yes	Yes	Yes	Yes	Yes	8 – Good
Logrono et al. – 1997[74]	Yes	Yes	Yes	Yes	Yes	Yes	No	Yes	7 – Good
Mezer et al. – 1997[75]	Yes	Yes	Yes	Yes	Yes	Yes	Yes	Yes	8 – Good
Sekundo et al. – 1997[76]	Yes	Yes	Yes	Yes	Yes	Yes	Yes	Yes	8 – Good
Zambarakji et al. – 1997[77]	Yes	Yes	Yes	Yes	Yes	No	Yes	Yes	7 – Good
Font et al. – 1998[78]	Yes	Yes	Yes	Yes	Yes	Yes	Yes	Yes	8 – Good
Garcia et al. – 1998[79]	Yes	Yes	Yes	Yes	Yes	Yes	Yes	Yes	8 – Good
Schaefer-Hirschkind et al. – 1998[80]	Yes	Yes	Yes	Yes	Yes	Yes	Yes	Yes	8 – Good
Toller et al. – 1998[81]	Yes	Yes	Yes	Yes	Yes	Yes	Yes	Yes	8 – Good
Collins et al. – 1999[82]	Yes	Yes	Yes	Yes	Yes	No	No	Yes	6 – Good
Scoyler et al. – 1999[83]	Yes	Yes	Yes	Yes	Yes	No	No	Yes	6 – Good
Wein et al. – 1999[84]	Yes	Yes	Yes	Yes	Yes	Yes	Yes	Yes	8 – Good
Wolstencroft et al. – 1999[85]	Yes	Yes	Yes	Yes	Yes	Yes	Yes	Yes	8 – Good
Callejo et al. – 2000[86]	Yes	Yes	Yes	Yes	Yes	Yes	Yes	Yes	8 – Good
Daumerie et al. – 2000[87]	Yes	Yes	Yes	Yes	Yes	Yes	Yes	Yes	8 – Good
Friedrich et al. – 2000[88]	Yes	Yes	Yes	Yes	Yes	Yes	Yes	Yes	8 – Good
Stuntz et al. – 2000[89]	Yes	Yes	Yes	Yes	Yes	Yes	Yes	Yes	8 – Good
Bartley et al. – 2001[90]	Yes	Yes	Yes	Yes	Yes	Yes	Yes	Yes	8 – Good

Maheshwari et al. – 2002[92]	Yes	Yes	Yes	Yes	Yes	Yes	Yes	Yes	8 – Good
McCulley et al. – 2002[93]	Yes	Yes	Yes	Yes	Yes	No	No	Yes	6 – Good
Misra et al. – 2002[94]	Yes	Yes	Yes	Yes	Yes	No	Yes	Yes	7 – Good
Baltogiannis et al. – 2003[95]	Yes	Yes	Yes	Yes	Yes	No	No	Yes	6 – Good
Fynn-Thompson et al. – 2003[97]	Yes	Yes	Yes	Yes	Yes	Yes	No	Yes	7 – Good
Lekse et al. – 2003[98]	Yes	Yes	Yes	Yes	Yes	Yes	Yes	Yes	8 – Good
Saleh et al. – 2003[100]	Yes	Yes	Yes	Yes	Yes	Yes	No	Yes	7 – Good
Takemoto et al. – 2003[101]	Yes	Yes	Yes	Yes	Yes	No	No	Yes	6 – Good
Tehrani et al. – 2003[102]	Yes	Yes	Yes	Yes	Yes	Yes	Yes	Yes	8 – Good
Chua et al. – 2004[104]	Yes	Yes	Yes	Yes	Yes	Yes	Yes	No	7 – Good
Glazer-Hockstein et al. – 2004[105]	Yes	Yes	Yes	Yes	Yes	No	No	Yes	6 – Good
Lell et al. – 2004[106]	Yes	Yes	Yes	Yes	Yes	No	No	Yes	6 – Good
Van der Zee et al. – 2004[107]	Yes	Yes	Yes	Yes	Yes	Yes	Yes	Yes	8 – Good
Zdinak et al. – 2004[108]	Yes	Yes	Yes	Yes	Yes	Yes	Yes	Yes	8 – Good
Borota et al. – 2005[109]	Yes	Yes	Yes	Yes	Yes	Yes	Yes	Yes	8 – Good
Challangudia et al. – 2005[110]	Yes	No	Yes	Yes	Yes	Yes	Yes	Yes	7 – Good
Fabi et al. – 2005[111]	Yes	Yes	Yes	Yes	Yes	Yes	Yes	Yes	8 – Good
Gupta et al. – 2005[112]	Yes	Yes	Yes	Yes	Yes	Yes	Yes	Yes	8 – Good
Hart et al. – 2005[113]	Yes	Yes	Yes	Yes	Yes	No	No	Yes	6 – Good
Konuk et al. – 2005[114]	Yes	Yes	Yes	Yes	Yes	Yes	Yes	Yes	8 – Good
Mohadjer et al. – 2005[115]	Yes	Yes	Yes	Yes	Yes	Yes	Yes	Yes	8 – Good
Puglisi et al. – 2005[116]	Yes	Yes	Yes	Yes	Yes	Yes	No	Yes	7 – Good
Aralikatti et al. – 2006[117]	Yes	Yes	Yes	Yes	Yes	No	Yes	Yes	7 – Good
Lee et al. – 2006[118]	Yes	Yes	Yes	Yes	Yes	Yes	Yes	Yes	8 – Good
Oida et al. – 2006[120]	Yes	Yes	Yes	Yes	Yes	Yes	Yes	Yes	8 – Good
Sharma et al. – 2006[122]	Yes	Yes	Yes	Yes	Yes	Yes	No	Yes	7 – Good
Singh et al. – 2006[123]	Yes	Yes	Yes	Yes	Yes	Yes	No	Yes	7 – Good
Solari et al. – 2006[124]	Yes	Yes	Yes	Yes	Yes	Yes	Yes	Yes	8 – Good
Tumuluri et al. – 2006[125]	Yes	Yes	Yes	Yes	Yes	Yes	Yes	Yes	8 – Good
Yunker et al. – 2006[126]	Yes	Yes	Yes	Yes	Yes	No	No	Yes	6 – Good
Char et al. – 2007[127]	Yes	Yes	Yes	Yes	Yes	No	No	Yes	6 – Good
Gerencer et al. – 2007[128]	Yes	Yes	Yes	Yes	Yes	Yes	Yes	Yes	8 – Good
Isshiki et al. – 2007[129]	Yes	Yes	Yes	Yes	Yes	Yes	Yes	Yes	8 – Good
Lin et al. – 2007[130]	Yes	Yes	Yes	Yes	Yes	Yes	Yes	Yes	8 – Good
Mani et al. – 2007[131]	Yes	Yes	Yes	Yes	Yes	Yes	No	Yes	7 – Good
Su et al. – 2007[132]	Yes	Yes	Yes	Yes	Yes	Yes	Yes	Yes	8 – Good
Talwar et al. – 2007[133]	Yes	Yes	Yes	Yes	Yes	No	No	Yes	6 – Good
Uluocak et al. – 2007[135]	Yes	Yes	Yes	Yes	Yes	Yes	Yes	Yes	8 – Good
Alsuhaibani et al. – 2008[136]	Yes	Yes	Yes	Yes	Yes	Yes	Yes	Yes	8 – Good
Hatton et al. – 2008[137]	Yes	Yes	Yes	Yes	Yes	Yes	Yes	Yes	8 – Good
Henning et al. – 2008[138]	Yes	Yes	Yes	Yes	Yes	Yes	Yes	Yes	8 – Good
Hirunwiwatkul et al. – 2008[139]	Yes	Yes	Yes	Yes	Yes	Yes	Yes	Yes	8 – Good
Kiratli et al. – 2008[140]	Yes	Yes	Yes	Yes	Yes	Yes	No	Yes	7 – Good
Kuo et al. – 2008[141]	Yes	Yes	Yes	Yes	Yes	Yes	No	Yes	7 – Good
Milman et al. – 2008[142]	Yes	Yes	Yes	Yes	Yes	Yes	Yes	Yes	8 – Good
Mudiyanselage et al. – 2008[143]	Yes	Yes	Yes	Yes	Yes	No	No	Yes	6 – Good

Peng et al. – 2008[144]	Yes	Yes	Yes	Yes	Yes	Yes	Yes	Yes	8 – Good
Polito et al. – 2008[146]	Yes	Yes	Yes	Yes	Yes	Yes	Yes	Yes	8 – Good
Preechawai et al. – 2008[147]	Yes	Yes	Yes	Yes	Yes	No	Yes	Yes	7 – Good
Rocha Filho et al. – 2008[148]	Yes	Yes	Yes	Yes	Yes	No	Yes	Yes	7 – Good
Seiff et al. – 2008[149]	Yes	Yes	Yes	Yes	Yes	No	No	Yes	6 – Good
Souayah et al. – 2008[150]	Yes	Yes	Yes	Yes	Yes	No	Yes	Yes	7 – Good
Surace et al. – 2008[151]	Yes	Yes	Yes	Yes	Yes	No	Yes	Yes	7 – Good
Gosslee et al. – 2009[152]	Yes	Yes	Yes	Yes	Yes	No	Yes	Yes	7 – Good
Nirmala et al. – 2009[153]	Yes	Yes	Yes	Yes	Yes	No	Yes	Yes	7 – Good
Slagle et al. – 2009[154]	Yes	Yes	Yes	Yes	Yes	Yes	Yes	Yes	8 – Good
Barahimi et al. – 2010[156]	Yes	Yes	Yes	Yes	Yes	Yes	Yes	Yes	8 – Good
Foo et al. – 2010[157]	Yes	Yes	Yes	Yes	Yes	Yes	Yes	Yes	8 – Good
Francone et al. – 2010[158]	Yes	Yes	Yes	Yes	Yes	No	Yes	Yes	7 – Good
Hassane et al. – 2010[159]	Yes	Yes	Yes	Yes	Yes	No	Yes	Yes	7 – Good
Matsuo et al. – 2010[160]	Yes	Yes	Yes	Yes	Yes	No	Yes	Yes	7 – Good
Ng et al. – 2010[161]	Yes	Yes	Yes	Yes	Yes	Yes	Yes	Yes	8 – Good
Patel et al. – 2010[162]	Yes	Yes	Yes	Yes	Yes	Yes	Yes	Yes	8 – Good
Eckardt et al. – 2011[163]	Yes	Yes	Yes	Yes	Yes	No	Yes	Yes	7 – Good
Galm et al. – 2011[164]	Yes	Yes	Yes	Yes	Yes	Yes	Yes	Yes	8 – Good
Guerriero et al. – 2011[165]	Yes	Yes	Yes	Yes	Yes	No	Yes	Yes	7 – Good
Gupta et al. – 2011[166]	Yes	Yes	Yes	Yes	Yes	Yes	Yes	Yes	8 – Good
Janicijevic-Petrovic et al. – 2011[167]	Yes	Yes	Yes	Yes	Yes	No	No	Yes	6 – Good
Konoglou et al. – 2011[168]	Yes	Yes	Yes	Yes	Yes	Yes	Yes	Yes	8 – Good
Li et al. – 2011[169]	Yes	Yes	Yes	Yes	Yes	No	Yes	Yes	7 – Good
Murthy et al. – 2011[170]	Yes	Yes	Yes	Yes	Yes	Yes	Yes	Yes	8 – Good
Mustapha et al. – 2011[171]	Yes	Yes	Yes	Yes	Yes	No	No	Yes	6 – Good
Rosado et al. – 2011[172]	Yes	Yes	Yes	Yes	Yes	Yes	Yes	Yes	8 – Good
Zarogoulidis et al. – 2011[173]	Yes	Yes	Yes	Yes	Yes	No	Yes	Yes	7 – Good
Charles et al. – 2012[174]	Yes	Yes	Yes	Yes	Yes	No	No	Yes	6 – Good
Chen et al. – 2012[175]	Yes	Yes	Yes	Yes	Yes	Yes	Yes	Yes	8 – Good
Dmuchowska et al. – 2012[176]	Yes	Yes	Yes	Yes	Yes	No	Yes	Yes	7 – Good
Jiang et al. – 2012[177]	Yes	Yes	Yes	Yes	Yes	No	Yes	Yes	7 – Good
Jung et al. – 2012[4178]	Yes	Yes	Yes	Yes	Yes	Yes	Yes	Yes	8 – Good
Kim et al. – 2012[179]	Yes	Yes	Yes	Yes	Yes	No	Yes	Yes	7 – Good
Pecen et al. – 2012[180]	Yes	Yes	Yes	Yes	Yes	No	Yes	Yes	7 – Good
SooHoo et al. – 2012[181]	Yes	Yes	Yes	Yes	Yes	No	Yes	Yes	7 – Good
Tomizawa et al. – 2012[182]	Yes	Yes	Yes	Yes	Yes	No	Yes	Yes	7 – Good
Wladis et al. – 2012[183]	Yes	Yes	Yes	Yes	Yes	Yes	Yes	Yes	8 – Good
Woo et al. – 2012[184]	Yes	Yes	Yes	Yes	Yes	Yes	No	Yes	7 – Good
Dobson et al. – 2013[185]	Yes	Yes	Yes	Yes	Yes	Yes	Yes	Yes	8 – Good
Fini et al. – 2013[186]	Yes	Yes	Yes	Yes	Yes	Yes	No	Yes	7 – Good
Fujimoto et al. – 2013[187]	Yes	Yes	Yes	Yes	Yes	Yes	Yes	Yes	8 – Good
Patel et al. – 2013[188]	Yes	Yes	Yes	Yes	Yes	Yes	Yes	Yes	8 – Good
Patel et al. – 2013[189]	Yes	Yes	Yes	Yes	Yes	No	No	Yes	6 – Good
Coloma-Gonzalez et al. – 2014[190]	Yes	Yes	Yes	Yes	Yes	Yes	Yes	Yes	8 – Good
Kapur et al. – 2014[193]	Yes	Yes	Yes	Yes	Yes	Yes	Yes	Yes	8 – Good

Ludmir et al. – 2014[194]	Yes	Yes	Yes	Yes	Yes	Yes	Yes	Yes	8 – Good
Saffra et al. – 2014[195]	Yes	Yes	Yes	Yes	Yes	Yes	Yes	Yes	8 – Good
Gupta et al. – 2015[196]	Yes	Yes	Yes	Yes	Yes	Yes	Yes	Yes	8 – Good
Pagsisihan et al. – 2015[199]	Yes	Yes	Yes	Yes	Yes	Yes	Yes	Yes	8 – Good
Pearlman et al. – 2015[200]	Yes	Yes	Yes	Yes	Yes	Yes	No	Yes	7 – Good
Rajabi et al. – 2015[201]	Yes	Yes	Yes	Yes	Yes	Yes	No	Yes	7 – Good
Arthur et al. – 2016[202]	Yes	Yes	Yes	Yes	Yes	Yes	No	Yes	7 – Good
Foster et al. – 2016[203]	Yes	Yes	Yes	Yes	Yes	Yes	No	Yes	7 – Good
MacLean et al. – 2016[204]	Yes	Yes	Yes	Yes	Yes	Yes	Yes	Yes	8 – Good
Pokharel et al. – 2016[205]	Yes	Yes	Yes	Yes	Yes	No	No	Yes	6 – Good
Tabai et al. – 2016[206]	Yes	Yes	Yes	Yes	Yes	Yes	No	Yes	7 – Good
Tun et al. – 2016[207]	Yes	Yes	Yes	Yes	Yes	No	No	Yes	6 – Good
AufderHeide et al. – 2017[208]	Yes	Yes	Yes	Yes	Yes	No	No	Yes	6 – Good
Huang et al. – 2017[210]	Yes	Yes	Yes	Yes	Yes	No	Yes	Yes	7 – Good
Mărginean et al. – 2017[211]	Yes	Yes	Yes	Yes	Yes	No	No	Yes	6 – Good
Nabeel et al. – 2017[212]	Yes	Yes	Yes	Yes	Yes	Yes	Yes	Yes	8 – Good
Nash et al. – 2017[213]	Yes	Yes	Yes	Yes	Yes	Yes	Yes	Yes	8 – Good
Rakul Nambiar et al. – 2017[214]	Yes	Yes	Yes	Yes	Yes	Yes	Yes	Yes	8 – Good
Rasool et al. – 2017[215]	Yes	Yes	Yes	Yes	Yes	Yes	Yes	Yes	8 – Good
Wang et al. – 2017[216]	Yes	Yes	Yes	Yes	Yes	Yes	Yes	Yes	8 – Good
Coutinho et al. – 2018[217]	Yes	Yes	Yes	Yes	Yes	Yes	Yes	Yes	8 – Good
Dutton et al. – 2018[219]	Yes	Yes	Yes	Yes	Yes	Yes	Yes	Yes	8 – Good
Gulmez-Sevim et al. – 2018[220]	Yes	Yes	Yes	Yes	Yes	No	No	Yes	6 – Good
Nifosì et al. – 2018[221]	Yes	Yes	Yes	Yes	Yes	Yes	Yes	Yes	8 – Good
Pinto-Proença et al. – 2018[222]	Yes	Yes	Yes	Yes	Yes	Yes	Yes	Yes	8 – Good
Ramsey et al. – 2018[223]	Yes	Yes	Yes	Yes	Yes	Yes	Yes	Yes	8 – Good
Roelofs et al. – 2018[224]	Yes	Yes	Yes	Yes	Yes	Yes	No	Yes	7 – Good
Rong et al. – 2018[225]	Yes	Yes	Yes	Yes	Yes	Yes	No	Yes	7 – Good
Shaikh et al. – 2018[226]	Yes	Yes	Yes	Yes	Yes	No	No	Yes	6 – Good
Chumdermpadetsuk et al. – 2019[227]	Yes	Yes	Yes	Yes	Yes	Yes	Yes	Yes	8 – Good
Crisostomo et al. – 2019[228]	Yes	Yes	Yes	Yes	Yes	Yes	No	Yes	7 – Good
Framarino-dei-Malatesta et al. – 2019[231]	Yes	Yes	Yes	Yes	Yes	Yes	Yes	Yes	8 – Good
Hernandez Pardines et al. – 2019[232]	Yes	Yes	Yes	Yes	Yes	Yes	No	Yes	7 – Good
Kagusa et al. – 2019[233]	Yes	Yes	Yes	Yes	Yes	Yes	Yes	Yes	8 – Good
Kiziloğlu et al. – 2019[234]	Yes	Yes	Yes	Yes	Yes	Yes	Yes	Yes	8 – Good
Kluska et al. – 2019[235]	Yes	Yes	Yes	Yes	Yes	Yes	No	Yes	7 – Good
Kozubowska et al. – 2019[236]	Yes	Yes	Yes	Yes	Yes	Yes	No	No	6 – Good
Sochat et al. – 2019[238]	Yes	Yes	Yes	Yes	Yes	Yes	Yes	Yes	8 – Good
Tao et al. – 2019[239]	Yes	Yes	Yes	Yes	Yes	Yes	Yes	Yes	8 – Good
Yadav et al. – 2019[240]	Yes	Yes	Yes	Yes	Yes	Yes	No	Yes	7 – Good
Yu et al. – 2019[241]	Yes	Yes	Yes	Yes	Yes	Yes	No	Yes	7 – Good
Bacorn et al. – 2020[242]	Yes	Yes	Yes	Yes	Yes	Yes	No	Yes	7 – Good
El Bakraoui et al. – 2020[244]	Yes	Yes	Yes	Yes	Yes	Yes	Yes	Yes	8 – Good
Halliday et al. – 2020[245]	Yes	Yes	Yes	Yes	Yes	Yes	Yes	Yes	8 – Good
Hoenck et al. – 2020[246]	Yes	Yes	Yes	Yes	Yes	Yes	No	Yes	7 – Good
Long et al. – 2020[247]	Yes	Yes	Yes	Yes	Yes	Yes	Yes	Yes	8 – Good

Madabhavi et al. – 2020[248]	Yes	Yes	Yes	Yes	Yes	Yes	Yes	Yes	8 – Good
Mahuvakar et al. – 2020[249]	Yes	Yes	Yes	Yes	Yes	Yes	Yes	Yes	8 – Good
Marotta et al. – 2020[250]	Yes	Yes	Yes	Yes	Yes	Yes	Yes	Yes	8 – Good
Mian et al. – 2020[251]	Yes	Yes	Yes	Yes	Yes	Yes	Yes	No	7 – Good
Narayanan et al. – 2020[253]	Yes	Yes	Yes	Yes	Yes	Yes	No	Yes	7 – Good
Pastore et al. – 2020[254]	Yes	Yes	Yes	Yes	Yes	Yes	Yes	Yes	8 – Good
Protopapa et al. – 2020[255]	Yes	Yes	Yes	Yes	Yes	Yes	No	Yes	7 – Good
Tsuruta et al. – 2020[257]	Yes	Yes	Yes	Yes	Yes	Yes	No	Yes	7 – Good
Wada et al. – 2020[258]	Yes	Yes	Yes	Yes	Yes	Yes	Yes	Yes	8 – Good
Mendia et al. – 2021[260]	Yes	Yes	Yes	Yes	Yes	Yes	Yes	Yes	8 – Good
Oprean et al. – 2021[261]	Yes	Yes	Yes	Yes	Yes	Yes	Yes	Yes	8 – Good
Razem et al. – 2021[262]	Yes	Yes	Yes	Yes	Yes	Yes	No	Yes	7 – Good

References found in Supplementary File 2.

**Joanna Briggs Institute Checklist for Case Series – Criteria**
1. Were there clear criteria for inclusion in the case series?
2. Was the condition measured in a standard, reliable way for all participants included in the case series?
3. Were valid methods used for identification of the condition for all participants included in the case series?
4. Did the case series have consecutive inclusion of participants?
5. Did the case series have complete inclusion of participants?
6. Was there clear reporting of the demographics of the participants in the study?
7. Was there clear reporting of clinical information of the participants?
8. Were the outcomes or follow up results of cases clearly reported?
9. Was there clear reporting of the presenting site(s)/clinic(s) demographic information?
10. Was statistical analysis appropriate?
**Responses Options**: Yes, No, Unclear, Not Applicable (NA)
**Quality Rating**: Poor 0 – 3; Fair 4 – 7; Good 8 – 10

**Study (Case Series)**	**1**	**2**	**3**	**4**	**5**	**6**	**7**	**8**	**9**	**10**	**Appraisal**
Mortada et al. – 1968[3]	Yes	Yes	Yes	Yes	Yes	Yes	Yes	Yes	No	NA	8 – Good
Ashton et al. – 1974[5]	Yes	Yes	Yes	Yes	Yes	Yes	Yes	Yes	No	NA	8 – Good
Huh et al. – 1974[6]	Yes	Yes	Yes	Yes	No	Yes	Yes	Yes	Yes	NA	8 – Good
Riddle et al. – 1982[21]	Yes	Yes	Yes	Yes	Yes	Yes	Yes	Yes	No	NA	8 – Good
Tertzakian et al. – 1982[22]	Yes	Yes	Yes	Yes	Yes	Yes	Yes	Yes	No	NA	8 – Good
Mortada et al. – 1984[24]	Yes	Yes	Yes	Yes	Yes	Yes	Yes	Yes	No	NA	8 – Good
Bond et al. – 1986[27]	Yes	Yes	Yes	Yes	Yes	Yes	Yes	Yes	No	NA	8 – Good
Stefanyszyn et al. – 1987[36]	Yes	Yes	Yes	Yes	Yes	Yes	Yes	Yes	No	NA	8 – Good
Boldt et al. – 1988[37]	Yes	Yes	Yes	Yes	Yes	Yes	Yes	Yes	Yes	NA	9 – Good
Orcutt et al. – 1988[38]	Yes	Yes	Yes	Yes	Yes	Yes	Yes	Yes	No	NA	8 – Good
Jacobs et al. – 1988[39]	Yes	Yes	Yes	Yes	Yes	No	Yes	Yes	Yes	NA	8 – Good
Shields et al. – 1988[40]	Yes	Yes	Yes	Yes	Yes	Yes	Yes	Yes	No	NA	8 – Good
Capone et al. – 1990[44]	Yes	Yes	Yes	Yes	Yes	Yes	Yes	Yes	No	NA	8 – Good
Goldberg et al. – 1990[46]	Yes	Yes	Yes	Yes	Yes	Yes	Yes	Yes	Yes	NA	9 – Good
Shetlar et al. – 1990[47]	Yes	Yes	Yes	Yes	Yes	Yes	Yes	Yes	No	NA	8 – Good
Tijl et al. – 1992[56]	Yes	Yes	Yes	Yes	Yes	Yes	Yes	Yes	Yes	NA	9 – Good
Fan et al. – 1995[66]	Yes	Yes	Yes	Yes	Yes	Yes	Yes	Yes	No	NA	8 – Good
Char et al. – 1997[71]	Yes	Yes	Yes	Yes	Yes	No	Yes	Yes	Yes	Yes	9 – Good
Shields et al. – 2001[91]	Yes	Yes	Yes	Yes	Yes	No	Yes	Yes	Yes	NA	8 – Good
Baroody et al. – 2003[96]	Yes	Yes	Yes	Yes	Yes	No	Yes	Yes	Yes	NA	8 – Good
Holland et al. – 2003[98]	Yes	Yes	Yes	Yes	Yes	No	Yes	Yes	Yes	NA	8 – Good
Zografos et al. – 2003[103]	Yes	Yes	Yes	Yes	Yes	Yes	Yes	Yes	No	NA	8 – Good
Mehta et al. – 2006[119]	Yes	Yes	Yes	Yes	Yes	No	Yes	Yes	Yes	Yes	9 – Good
Schick et al. – 2006[121]	Yes	Yes	Yes	Yes	Yes	Yes	Yes	Yes	No	NA	8 – Good

Torres et al. – 2007[134]	Yes	Yes	Yes	Yes	Yes	Yes	Yes	Yes	No	NA	8 – Good
Pitts et al. – 2008[145]	Yes	Yes	Yes	Yes	Yes	Yes	Yes	Yes	No	NA	8 – Good
Valenzuela et al. – 2009[155]	Yes	Yes	Yes	Yes	Yes	No	Yes	Yes	No	Yes	8 – Good
Eldesouky et al. – 2014[191]	Yes	Yes	Yes	Yes	Yes	Yes	Yes	Yes	No	NA	8 – Good
Greene et al. – 2014[192]	Yes	Yes	Yes	Yes	Yes	Yes	Yes	Yes	Yes	NA	9 – Good
Magliozzi et al. – 2015[197]	Yes	Yes	Yes	Yes	Yes	No	Yes	Yes	Yes	Yes	9 – Good
Magrath et al. – 2015[198]	Yes	Yes	Yes	Yes	Yes	Yes	Yes	Yes	No	NA	8 – Good
Geske et al. – 2017[209]	Yes	Yes	Yes	Yes	Yes	Yes	Yes	Yes	No	NA	8 – Good
Das et al. – 2018[218]	Yes	Yes	Yes	Yes	Yes	Yes	Yes	Yes	Yes	NA	9 – Good
Espinoza-Barberi et al. – 2019[231]	Yes	Yes	Yes	Yes	Yes	Yes	Yes	Yes	No	NA	8 – Good
Rider et al. – 2019[237]	Yes	Yes	Yes	Yes	Yes	Yes	Yes	Yes	No	NA	8 – Good
Blohmer et al. – 2020[243]	Yes	Yes	Yes	Yes	No	No	Yes	Yes	Yes	Yes	8 – Good
Montejano-Milner et al. – 2020[252]	Yes	Yes	Yes	Yes	Yes	Yes	Yes	Yes	No	Yes	9 – Good
Sindoni et al. – 2020[256]	Yes	Yes	Yes	Yes	Yes	No	Yes	Yes	Yes	Yes	9 – Good
El-Khazen Dupuis et al. – 2021[259]	Yes	Yes	Yes	Yes	Yes	Yes	Yes	Yes	No	NA	8 - Good

References found in Supplementary File 2.
